# Supplementary material for: Study protocol: an open-label individually randomised controlled trial to assess the efficacy of artemether-lumefantrine prophylaxis for malaria among forest goers in Cambodia
Source: BMJ Open. 2021 Jul 7;11(7):e045900. doi: 10.1136/bmjopen-2020-045900 (PMC8264911; doi:10.1136/bmjopen-2020-045900)
Supplement: Supplementary data [file bmjopen-2020-045900supp001.pdf]

BMJ Publishing Group Limited (BMJ) disclaims all liability and responsibility arising from any reliance placed on this supplemental material which has been supplied by the author(s)

Schedule of Assessments

TEST/APPLICATION	SCREENING	D0	D1	D2	CCM*	D28-35	CCM*	D56-63	CCM*	D84-91	CCM* up to D112
Informed consent	X										
Demographics		X									
Risk history		X				X		X		X	
Travel history		X				X		X		X	
Medical and drug history		X									
Symptoms questionnaire		X				X		X		X	
Temperature		X									
Weight		X									
Randomisation and assign study ID		X									
AL/multivitamin doses to be given		X	X	X							
Plasmodium PCR		X				X		X		X	
Blood for storage (serology)		X									
Plasmodium genetic analysis (DBS)					X*		X*		X*		X*
*CCM = episode of confirmed clinical malaria between enrolment and follow-up											

Dosing Schedules

Artemether-lumefantrine

Artemether-lumefantrine dosing schedule										
One tablet AL contains 20 mg artemether and 120 mg lumefantrine (Coartem)										
	No. of tablets recommended at approximate timing of dosing									
	Day 1		Day 2		Day 3		Day 8		Weekly	
Weight: Kilogram	0h	8h	24h	36h	48h	60h	168h	176h	336h …	344h …
15 - <25	2	2	2	2	2	2	2	2	2	2
25 - <35	3	3	3	3	3	3	3	3	3	3
≥35	4	4	4	4	4	4	4	4	4	4
Alternative preparation: one tablet AL contains 80 mg artemether and 480 mg lumefantrine (Artefan)										
	No. of tablets recommended at approximate timing of dosing									
	Day 1		Day 2		Day 3		Day 8		Weekly	
Weight: Kilogram	0h	8h	24h	36h	48h	60h	168h	176h	336h …	344h …
≥35	1	1	1	1	1	1	1	1	1	1

Multivitamin

Multivitamin dosing schedule					
One tablet contains Vitamin-A : 5000 USP units<br>Vitamin D: 400 USP Units<br>Ascorbic acid: 75 mg<br>Thiamine Mononitrate: 2 mg<br>Riboflavin: 3 mg<br>Niacin amide: 20 mg<br>Or suitable equivalent alternative					
	No. of tablets recommended at approximate timing of dosing				
	Day 1	Day 2	Day 3	Day 8	Weekly
Weight: Kilogram	0h	24h	48h	168h	336h …
≥15	1	1	1	1	1
