# Supplementary material for: Study protocol: an open-label individually randomised controlled trial to assess the efficacy of artemether-lumefantrine prophylaxis for malaria among forest goers in Cambodia
Source: BMJ Open. 2021 Jul 7;11(7):e045900. doi: 10.1136/bmjopen-2020-045900 (PMC8264911; doi:10.1136/bmjopen-2020-045900)
Supplement: Supplementary data [file bmjopen-2020-045900supp002.pdf]

Participant Information Sheet

In this information sheet, we will give you information about the study to help you decide whether or not you agree to take part in this study. If you have any questions or concerns, please ask the study staff to help explain until you fully understand. You may ask to take this document home to discuss with relatives, siblings, close friends, or your doctor to assist you in making a decision to take part in this study.

**Lay Title**	Study to assess efficacy of antimalarial drugs to prevent forest goers from getting malaria in Cambodia
**Study Title (official)**	An open-label individually randomised controlled trial to assess the efficacy of artemether-lumefantrine prophylaxis for malaria among forest goers in Cambodia
**Principal Investigator**	Professor R.J. Maude
**Sponsor**	University of Oxford

If you are reading this to consider enrolling your child, please know that whenever it refers to 'you' it can be taken to mean 'your child'.

What is the purpose of this research?

In Cambodia, the number of malaria cases for forest goers is still high, due to the mobile nature of their work and their habit of sleeping overnight in the forests, often without mosquito nets. Therefore, we would like to do research on how to prevent malaria for forest goers. This study plans to recruit up to 4,400 participants from 50 villages in Stung Treng and Pursat Provinces who are planning to travel to the forest within the next 72 hours.

What will happen to you if you participate in the study?

We would like to invite you to join the study because you are 16 and 65 years of age and planning to visit the forest within 72 hours and stay overnight. The study staff will explain to you about details of the study and answer any questions you may have. If you are interested in joining the study, you will be asked to sign an informed consent form and then follow study procedures. You will go through study visits as below;

Screening and baseline assessment visit

During this visit, study staff will ask you questions about your health status and medication that you used and are currently using, risk factors for malaria, recent travel history and where you live and will go through a brief physical examination. If you pass all of the study selection criteria, you can take part in this study.

All the study participants will received either artemether-lumefantrine or multivitamin given by mouth over 3 days. The decision on which drug will be decided by random chance like a flip of a coin. This means that you will have the same chance of receiving artemether-lumefantrine or multivitamin. The artemether-lumefantrine should be taken with a small amount of fatty food or drink, e.g. a small carton of milk, so it is better absorbed into the body.

Participant Information Sheet

Before giving you the study drugs (artemether-lumefantrine or multivitamin), the study team will collect about 3.2 milliliters (around two thirds of a teaspoon) of your blood for malaria tests, and to measure the amount of lumefantrine present and some of this will be stored for testing later for other causes of infection.

The study team will observe when you take study drug on the first day. Where possible, study team will observe when you take study drug on days 2 and 3. If not possible, the study team will contact you by telephone or in person to ensure that you take the second and third doses of study drug and to ensure you follow below procedure in case of vomiting.

If you vomit within half an hour after taking the study drug, the full dose will be repeated. If vomiting occurs between half and one hour, half of the dose will be repeated. If vomiting occurs more than one hour after taking study drug, no repeat dosing will be done. If vomiting within 1 hour occurs more than one time, no repeat dosing is allowed.

In some participants, we would like to collect GPS coordinates of where you live and work and places where the malaria infection may occur, such as forests, farms or plantations. This may include being asked to carry a device to record your location over time which can choose not to carry if you are not comfortable doing so. We will collect all available local malaria treatment records to describe how the study participants compare to the overall population who receive treatment for malaria and this will allow us to better understand local malaria epidemiology and transmission patterns. All personal information will be anonymised so that no individual can be identified from their treatment records, through interviews, or from mapping data.

Follow-up visit every 28-35 days after starting prophylaxis

You will be asked to return for a follow-up visit any time from 28 to 35 days after starting prophylaxis. During this visit, we will ask you about time you spent in the forest, where you went, why and who you travelled with and about any risk factors of getting malaria infection, and any illnesses or malaria infection occurred from the last visit. In addition, we will collect around 1.6 milliliters (around a third of a teaspoon) of your blood for malaria tests and to measure the amount of lumefantrine present. We also will collect around 2 milliliters (around half a teaspoon) of your blood to test later for other causes of infection.

If you are planning to return to the forest again within the following 28 days after this visit, you will be asked to continue your weekly prophylaxis of study drugs the same as what you received before. You will then be asked to return for a second follow-up visit 28 to 35 days later when the above procedure will be repeated. This will be repeated for a possible third visit giving a maximum total of three periods of 28 to 35 days. You will receive the same study drug throughout.

If you then plan to visit the forest again following an interval of at least 28 days after the last follow-up visit, you may be enrolled again into the study when you would receive either artemether-lumefantrine or multivitamin as described above. The choice of artemether-lumefantrine or multivitamin may be different. This may be repeated up to 1 more time thus giving a maximum of 3 courses of study drug.

At each follow-up visit, if you are not planning to return to the forest in the next 28 days, you will be asked to take 4 weeks of prophylaxis from the time you were last in the forest.

Participant Information Sheet

If you are not well at any time during the study participation, such as high fever, you will be assessed and treated by the healthcare workers in the local healthcare system as per routine clinical practice. We will collect your blood for malaria tests.

Are there any risks or disadvantages to me for taking part?

Risk of blood withdrawal from the arm

The risks of blood withdrawal from the arm include discomfort, occasional bleeding or bruising of the skin at the site of needle puncture, and rarely infection. If an infection of the skin occurs, you will be taken care according to standard of care until you recover without any expenses.

Risk of Artemether-lumefantrine

Artemether-lumefantrine is registered for the treatment of malaria. It is usually very well tolerated. The most common side-effects are nausea, vomiting, abdominal pain and diarrhoea. Some people experience headache and dizziness.

Risks of multivitamin

The main side-effects of multivitamin are upset stomach, unpleasant taste or headache which are mild to moderate in nature. Very rarely, there may cause an allergic reaction.

There may be any side effects from study drugs that have not been noticed before. You should inform the study staff right away if you have any problems. If you have any side effects or any unexpected problems during participation in the study, we will treat these problems fully and with no charge to you.

The study drugs may not be safe for an unborn child. If you are female with child bearing potential, it is essential that you do not become pregnant during the study participation. This means not having sex or using a proven and effective method of birth control such as birth control pills, intrauterine contraceptive devices, vaginal contraceptive devices or hormonal implants. If you become pregnant, you will be followed to determine the outcome of the pregnancy. Please tell us as quickly as possible when you become pregnant during the study.

If any new information about the safety of the study drugs becomes available, we will tell you as soon as possible.

Protection against malaria

It is not known how effective artemether-lumefantrine is to prevent malaria and that the multivitamin will provide no protection against malaria. You should therefore assume that you are not protected from catching malaria by taking these medicines

What are the benefit of taking part in this study?

The results of the study will help us to understanding if the Artemether-lumefantrine could protect forest workers from malaria infection. This also may help to predict its ability in reducing malaria transmission.

What will happen if you choose not to take part in the study, or if you change your mind after you agree?

Your participation in this study is entirely voluntary. After you join the study, you are also free to withdraw from the study at any time, without penalty or any effects on your medical treatment both now and in the future.

Maude RJ, *et al. BMJ Open* 2021; 11:e045900. doi: 10.1136/bmjopen-2020-045900

Participant Information Sheet

In addition, the study staff and the study sponsor have rights to withdraw you from this study if it's considered that it is in your best interest.

What will the blood sample be used for?

If you have confirmed malaria infection, your blood sample will be processed to analyse the DNA of the parasite. To do this test, we have to ship your blood sample to Molecular Tropical Medicine Laboratory, Bangkok, Thailand and/or Wellcome Trust Sanger Institute in Hinxton, UK or other suitable laboratory.

Total volume of blood collection will be approximately 9.6 milliliters (around two teaspoons) for the maximum of 84 days of follow up if you return for follow-up on 3 consecutive occasions and had all blood samples taken. This blood volume does not include any repeat blood test for which an additional 9.6 milliliters (around two teaspoons) may be needed if necessary.

If you consent, we would like to keep some of your blood samples for not longer than 10 years. Your blood samples will be labeled with a unique number but not with your name. Any additional testing apart from what indicated in this study will be tested only after permission by the ethics committee.

Will there be any financial cost or compensation for participating the study?

It should not cost you any money to join the study. You will be compensated for time lost from work as a result of study activities plus the cost of local transport to attend each study visit and the costs of meals on those days. *(The amounts in monetary terms will be determined by CNM according to local norms).*

Confidentiality

Your name will not be in any report or on any blood sample being collected during the study. The information we collected from you and from analysing of your blood samples will be kept confidential by the study team.

No one other than the study team, authorised personnel from the study sponsor, monitor, ethics committee and regulatory authorities are allowed direct access to personally identified study records.

If you allow all data collected from you including results from blood analyses that is stored in our database may be shared with other researchers to use in the future. The other researchers will not be given any information that could identify you.

Who can I contact if I have questions?

If you have any questions or concerns after reading this information sheet, you can discuss them with our study team. We will also available throughout the study to answer any questions or address any concerns that you may have later on.

If you have any questions, you can contact the following doctors/study staffs by telephone:

Name: *Telephone number:*

or

Name: *Telephone number:*

Maude RJ, *et al. BMJ Open* 2021; 11:e045900. doi: 10.1136/bmjopen-2020-045900

Participant Information Sheet

If you haven't been treated as specified in this information sheet or you wish to know the participant's rights, contact the secretariat office of the Ethics Committee. *<Ethics committee contact information>.*

Data protection

The University of Oxford is responsible for ensuring the safe and proper use of any personal information you provide, solely for research purposes.

Maude RJ, *et al. BMJ Open* 2021; 11:e045900. doi: 10.1136/bmjopen-2020-045900
