# Supplementary material for: Study protocol: an open-label individually randomised controlled trial to assess the efficacy of artemether-lumefantrine prophylaxis for malaria among forest goers in Cambodia
Source: BMJ Open. 2021 Jul 7;11(7):e045900. doi: 10.1136/bmjopen-2020-045900 (PMC8264911; doi:10.1136/bmjopen-2020-045900)
Supplement: Supplementary data [file bmjopen-2020-045900supp003.pdf]

Informed Consent Form

I would like to take part in the study, titled: **"**An open-label individually randomised controlled trial to assess the efficacy of artemether-lumefantrine prophylaxis for malaria among forest goers in Cambodia**".**

I have read the participant information sheet and have had the opportunity to ask questions about the study and any questions I have asked have been answered to my satisfaction.

I understand that I can withdraw myself/my child or stop taking part in the research at any time without affecting further services to which I am/my child is entitled in the future. To consent to take part in this study, I allow the study team to use my/my child's personal information obtained from this study.

If I have doubts about the study procedures or I/my child experience(s) any side effect from this study, I will be able to contact study staff at any time.

I fully understand the statements in the participant information sheet and this informed consent form, and consent to participate in this study.

And

I ☐ allow / ☐ do not allow my/my child's blood to be tested for other infectious diseases.

I ☐ allow / ☐ do not allow my/my child's blood to be stored for future studies.

I ☐ allow / ☐ do not allow my/my child's blood to be shipped abroad.

I ☐ allow / ☐ do not allow my/my child's data and results from blood analyses that is stored in the database to be shared with other researchers to use in the future.

I ☐ allow / ☐ do not allow my/my child's location to be recorded with a GPS device.

Signature of participant/parent or guardian (if child) ……..……………………………

Print name of participant/parent or guardian (if child) …………….…………………

Date ..…….…………………………

Signature of person conducting the informed consent ….…………………………

Print name of person conducting the informed consent …………………………..……

Date ……..………………………

THE PARTICIPANT SHOULD NOW BE GIVEN A SIGNED COPY TO KEEP

OxTREC ref: 23-19

PAL_Cambodia Informed Consent Form version 2.0 dated 25th June, 2019 *Page 1 of 2*

Informed Consent Form

For the participant who cannot read or sign in the consent form, the participant can thumb print in the following box.

I cannot read but study staffs have read information in this informed consent form to me and explain until I fully understand the given information. Therefore I provide my thumbprint to voluntary consent for myself taking part in this research study.

And

I ☐ allow / ☐ do not allow my/my child's blood to be tested for other infectious diseases.

I ☐ allow / ☐ do not allow my/my child's blood to be stored for future studies.

I ☐ allow / ☐ do not allow my/my child's blood to be shipped abroad.

I ☐ allow / ☐ do not allow my/my child's data and results from blood analyses that is stored in the database to be shared with other researchers to use in the future.

I ☐ allow / ☐ do not allow my/my child's location to be recorded with a GPS device.

☐

Right thumb print of the participant/parent or guardian (if child)

Signature of person conducting the informed consent ……………………………

Print name of person conducting the informed consent ……………………….……

Date …………….……………

Signature of witness …………………………………………….……

Print name of witness …………………………………………………

Date ……………………………………………...

THE PARTICIPANT SHOULD NOW BE GIVEN A SIGNED COPY TO KEEP

Maude RJ, *et al. BMJ Open* 2021; 11:e045900. doi: 10.1136/bmjopen-2020-045900
