# Supplementary material for: Study protocol: an open-label individually randomised controlled trial to assess the efficacy of artemether-lumefantrine prophylaxis for malaria among forest goers in Cambodia
Source: BMJ Open. 2021 Jul 7;11(7):e045900. doi: 10.1136/bmjopen-2020-045900 (PMC8264911; doi:10.1136/bmjopen-2020-045900)
Supplement: Author's manuscript [file bmjopen-2020-045900.draft_revisions.pdf]

BMJ Open is committed to open peer review. As part of this commitment we make the peer review history of every article we publish publicly available.

When an article is published we post the peer reviewers' comments and the authors' responses online. We also post the versions of the paper that were used during peer review. These are the versions that the peer review comments apply to.

The versions of the paper that follow are the versions that were submitted during the peer review process. They are not the versions of record or the final published versions. They should not be cited or distributed as the published version of this manuscript.

BMJ Open is an open access journal and the full, final, typeset and author-corrected version of record of the manuscript is available on our site with no access controls, subscription charges or pay-per-view fees (http://bmjopen.bmj.com).

If you have any questions on BMJ Open's open peer review process please email info.bmjopen@bmj.com

BMJ Open

An open-label individually randomised controlled trial to assess the efficacy of artemether-lumefantrine prophylaxis for malaria among forest goers in Cambodia

Journal:	*BMJ Open*
Manuscript ID	bmjopen-2020-045900
Article Type:	Protocol
Date Submitted by the Author:	18-Oct-2020
Complete List of Authors:	Maude, Richard; Mahidol University, Mahidol-Oxford Tropical Medicine Research Unit, Faculty of Tropical Medicine; University of Oxford, Centre for Tropical Medicine and Global Health, Nuffield Department of Medicine<br>Tripura, Rupam; Mahidol University, Mahidol-Oxford Tropical Medicine Research Unit, Faculty of Tropical Medicine<br>Ean, Mom; Mahidol University, Mahidol-Oxford Tropical Medicine Research Unit, Faculty of Tropical Medicine<br>Meas, Sokha; Mahidol University, Mahidol-Oxford Tropical Medicine Research Unit, Faculty of Tropical Medicine<br>Peto, Thomas; Mahidol University, Mahidol-Oxford Tropical Medicine Research Unit, Faculty of Tropical Medicine<br>Callery, James; Mahidol University, Mahidol-Oxford Tropical Medicine Research Unit, Faculty of Tropical Medicine<br>Imwong, Mallika; Mahidol University, Mahidol-Oxford Tropical Medicine Research Unit, Faculty of Tropical Medicine; Mahidol University, Department of Molecular Tropical Medicine and Genetics, Faculty of Tropical Medicine<br>Vongpromek, Ranitha; Mahidol University, Mahidol-Oxford Tropical Medicine Research Unit, Faculty of Tropical Medicine; WorldWide Antimalarial Resistance Network, Asia Regional Centre<br>Tarning, Joel; Mahidol University, Mahidol-Oxford Tropical Medicine Research Unit, Faculty of Tropical Medicine; University of Oxford, Centre for Tropical Medicine and Global Health, Nuffield Department of Medicine<br>Mukaka, Mavuto; Mahidol University, Mahidol-Oxford Tropical Medicine Research Unit, Faculty of Tropical Medicine; University of Oxford, Centre for Tropical Medicine and Global Health, Nuffield Department of Medicine<br>Waithira, Naomi; Mahidol University, Mahidol-Oxford Tropical Medicine Research Unit, Faculty of Tropical Medicine; University of Oxford, Centre for Tropical Medicine and Global Health, Nuffield Department of Medicine<br>Soviet, Oung; Provincial Health Department<br>von Seidlein, Lorenz; Mahidol University, Mahidol-Oxford Tropical Medicine Research Unit, Faculty of Tropical Medicine; University of Oxford, Centre for Tropical Medicine and Global Health, Nuffield Department of Medicine<br>Sovannaroth, Siv; National Center for Parasitology, Entomology and Malaria Control
Keywords:	Tropical medicine < INFECTIOUS DISEASES, Public health < INFECTIOUS DISEASES, Epidemiology < INFECTIOUS DISEASES

SCHOLARONE™
Manuscripts

I, the Submitting Author has the right to grant and does grant on behalf of all authors of the Work (as defined in the below author licence), an exclusive licence and/or a non-exclusive licence for contributions from authors who are: i) UK Crown employees; ii) where BMJ has agreed a CC-BY licence shall apply, and/or iii) in accordance with the terms applicable for US Federal Government officers or employees acting as part of their official duties; on a worldwide, perpetual, irrevocable, royalty-free basis to BMJ Publishing Group Ltd ("BMJ") its licensees and where the relevant Journal is co-owned by BMJ to the co-owners of the Journal, to publish the Work in this journal and any other BMJ products and to exploit all rights, as set out in our licence.

The Submitting Author accepts and understands that any supply made under these terms is made by BMJ to the Submitting Author unless you are acting as an employee on behalf of your employer or a postgraduate student of an affiliated institution which is paying any applicable article publishing charge ("APC") for Open Access articles. Where the Submitting Author wishes to make the Work available on an Open Access basis (and intends to pay the relevant APC), the terms of reuse of such Open Access shall be governed by a Creative Commons licence – details of these licences and which Creative Commons licence will apply to this Work are set out in our licence referred to above.

Other than as permitted in any relevant BMJ Author's Self Archiving Policies, I confirm this Work has not been accepted for publication elsewhere, is not being considered for publication elsewhere and does not duplicate material already published. I confirm all authors consent to publication of this Work and authorise the granting of this licence.

For peer review only

Title: An open-label individually randomised controlled trial to assess the efficacy of artemether-lumefantrine prophylaxis for malaria among forest goers in Cambodia

Short title: Study to assess efficacy of artemether-lumefantrine prophylaxis against forest malaria in Cambodia

Authors:
Richard J Maude[1,2,3,4*]
Rupam Tripura[1,2]
Ean Mom[1]
Meas Sohka[1]
Thomas J Peto[1,2]
James J Callery[1,2]
Mallika Imwong[1,5]
Ranitha Vongpromek[1,6]
Joel Tarning[1,2]
Mavuto Mukaka[1,2]
Naomi Waithira[1,2]
Oung Soviet[7]
Lorenz von Seidlein[1,2]
Siv Sovannaroth[7]

1. Mahidol-Oxford Tropical Medicine Research Unit, Faculty of Tropical Medicine, Mahidol University, Bangkok, Thailand
2. Centre for Tropical Medicine and Global Health, Nuffield Department of Medicine, University of Oxford, Oxford, UK
3. Harvard TH Chan School of Public Health, Harvard University, Boston, USA
4. The Open University, Milton Keynes, UK
5. Department of Molecular Tropical Medicine and Genetics, Faculty of Tropical Medicine, Mahidol University, Bangkok, Thailand
6. Worldwide Antimalarial Resistance Network (WWARN), Asia Regional Centre, Bangkok, Thailand
7. Provincial Health Department, Stung Treng, Cambodia
8. National Center for Parasitology, Entomology and Malaria Control, Phnom Penh, Cambodia

*corresponding author: richard@tropmedres.ac

For peer review only

ABSTRACT

Introduction

In the Greater Mekong Subregion (GMS) adults are at highest risk for malaria. The most relevant disease vectors bite during daytime and outdoors which makes forest work a high-risk activity for malaria. The absence of effective vector control strategies and limited periods of exposure during forest visits suggest that chemoprophylaxis could be an appropriate strategy to protect forest workers against malaria.

Methods and Analysis

The protocol describes an open label randomized controlled trial of artemether-lumefantrine (AL) versus multivitamin as prophylaxis against malaria among forest goers aged 16 to 65 years in rural northeast Cambodia. The primary objective is to compare the efficacy of the artemisinin combination therapy (ACT) AL versus a multivitamin preparation as defined by the 28-day PCR parasite positivity rate and incidence of confirmed clinical malaria of any species. The sample size is 2200 patient-episodes of duration 1 month in each arm. The duration of follow-up and prophylaxis for each participant is 1, 2 or 3 consecutive 28 day periods, followed by a further 28 days of post-exposure prophylaxis, depending on whether they continue to visit to the forest. Analysis will be done both by intention-to-treat and per-protocol.

Ethics and dissemination

All participants will provide written, informed consent. Ethical approval was obtained from the Oxford Tropical Research Ethics Committee and the Cambodia National Ethics Committee for Health Research. Results will be disseminated by peer-reviewed open access publication together with open data.

Registration details

https://clinicaltrials.gov/ct2/show/NCT04041973

ARTICLE SUMMARY

Strengths and limitations of the study

1. Malaria is a major health problem for forest workers in Cambodia. Preventing malaria will provide major health as well as socio-economic benefits for participants in the first instance and, if rolled out, for forest workers more broadly.
2. The trial intervention was designed together with the Cambodian government to be potentially implementable depending on the results.
3. Broad engagement with healthcare workers and communities in the study area preceded enrolment and this is continuing throughout.
4. Local healthcare workers and forest goers assist with running the trial including identifying potential participants and supporting follow-up
5. The trial is open label so participants can know which study drug they are taking.
6. The outcomes are dependent on the incidence of malaria during the trial follow-up period.

For peer review only

LIST OF ABBREVIATIONS

ACT	Artemisinin-based combination therapy
AE	Adverse event
A/L	Artemether-lumefantrine
AQ	Amodiaquine
CNM	National Center for Parasitology, Entomology and Malaria Control
CRF	Case record form
CTSG	Clinical Trials Support Group (MORU)
DHA	Dihydroartemisinin
DNA	Deoxyribonucleic Acid
EDC	Electronic data capture
EDTA	Ethylene-diamine-tetra-acetic acid
G6PD	Glucose-6-phosphate dehydrogenase
GCP	Good Clinical Practice
GPS	Global Positioning System
Hb	Haemoglobin
Hct	Haematocrit
IRS	Indoor residual spraying
LLIN	Long-lasting insecticide treated bednets
MDR1	Multi-Drug Resistance Gene 1
MQ	Mefloquine
MORU	Mahidol-Oxford Research Unit
NMCP	National Malaria Control Programme
PA	Pyronaridine-artesunate
PAL	Prophylaxis with artemether-lumefantrine study
PCR	Polymerase Chain Reaction
QA	Quality Assurance
QC	Quality Control
SAE	Serious Adverse Event
SNP	Single-nucleotide polymorphism
SOP	Standard Operating Procedure
WHO	World Health Organisation
WWARN	Worldwide Antimalarial Resistance Network

For peer review only

INTRODUCTION

In the Greater Mekong Subregion (GMS) adults are at highest risk for malaria. The most relevant disease vectors bite during daytime and outdoors which makes forest work a high-risk activity for malaria. The absence of effective vector control strategies and limited periods of exposure during forest visits suggest that chemoprophylaxis could be an appropriate strategy to protect forest workers against malaria.

In the Greater Mekong Subregion (GMS) a large proportion of malaria transmission occurs in forested areas, which serve as perpetual sources of transmission [1-5]. Studies have demonstrated increased risk of malaria among forest goers, particularly in men of working age [6, 7] although these have largely been restricted to small geographical areas. Protecting forest goers from Plasmodium infections would not only benefit them directly but also people residing around their home. Malaria elimination efforts which do not consider the reinfection risk from forest workers are unlikely to succeed. However, preventing infections in forest workers is a major challenge. The biting rhythm and resting behaviour of *Anopheles dirus* reduces the impact of the two most commonly employed control measures, long-lasting insecticide treated bednets (LLIN) and indoor residual spraying (IRS). Several studies have also demonstrated poor use of personal protection measures against malaria transmission [8-10]. Two factors that increase malaria risk among forest workers are the basic character of overnight forest accommodation [9] and exposure to the Anopheles vectors (e.g. *An. dirus*), which tend to bite outdoors in daytime. LLIN have a high protective efficacy against nocturnal, indoor malaria transmission [11] but are less protective against daytime, outdoor-biting vectors like An. dirus. The improvised housing of forest workers is frequently poorly suited to hanging bed nets.[12] Imaginative interventions such as supplying forest workers with insecticide treated hammocks do not address the biting rhythm and resting behaviour of the vectors and have a disappointing uptake in field studies.[10, 12] In the absence of simple, effective, and affordable vector control interventions, providing forest goers with effective antimalarial prophylaxis seems a promising alternative approach to protect them against malaria.[13]

We propose to evaluate the feasibility and protective efficacy of antimalarial prophylaxis during forest work. It has been demonstrated in sub-Saharan Africa that chemoprophylaxis (SMC) of children, the highest risk group for malaria in tropical Africa, can reduce malaria cases by 75%, is cost effective and safe and can be given by community health workers [14, 15]. We propose to provide chemoprophylaxis to forest workers, the population group with the highest malaria risk in the GMS. In the proposed study we compare chemoprophylaxis with an antimalarial drug, artemether-lumefantrine (AL) to a control agent, multivitamins. A recent mass drug administration in Cambodia demonstrated that DHA/piperaquine remains effective to clear low-density, subclinical *P. falciparum* infections, but there are increasing treatment failures of clinical malaria cases [16] and markers of resistance to piperaquine in Cambodia are increasing. Although artesunate-pyronaridine has recently been introduced for treatment in parts of Cambodia, there remain some unresolved concerns about potential liver toxicity[17]. Evidence to date suggests that efficacy of artemether-lumefantrine remains high in Cambodia and is very well tolerated with an excellent toxicity profile and is thus the preferred potential option for prophylaxis by the National Malaria Control Programme. However, it must be taken with fat to maximize absorption. Previously it has been difficult or impossible to detect very low-density Plasmodium infections. The availability of more sensitive PCR methods allows us to detect Plasmodium infections with much lower densities [18, 19]. By use of PCR, we will be able to detect a difference in the prevalence of low density, subclinical *P. falciparum* infections between the two study arms in a relatively small sample of study participants.

Chemoprophylaxis of forest workers could protect this high-risk group and could reduce or even interrupt transmission in villages. The highly encouraging results of seasonal malaria chemoprophylaxis (SMC) in selected regions of sub-Saharan Africa provide hope that targeting another high-risk group, forest workers, could reduce malaria transmission in Cambodia and the wider GMS. In sub-Saharan Africa, children remain the main risk group for Plasmodium infections. In SE Asia the main risk group are adults working and sleeping outdoors hence we propose to provide chemoprophylaxis for these adults. A major challenge for this strategy is the choice of an appropriate chemoprophylactic regimen in the GMS. The chemoprophylactic regimen of choice in Africa is sulfadoxine/pyrimethamine (S/P) plus amodiaquine despite high level resistance against the S/P component of the regimen. Similarly, we propose the use of AL, a drug whose efficacy

For peer review only - http://bmjopen.bmj.com/site/about/guidelines.xhtml

remains high in the GMS, unlike, for example DHA/piperaquine [20]. The proposed study will help to assess the efficacy and feasibility of prophylaxis to prevent malaria in forest workers, help to identify the optimal regimen, and predict its efficacy in reducing overall transmission. The proposed study is a critical step for future use of chemoprophylaxis to protect forest workers in the GMS against malaria.

Proposed activities

Artemether-lumefantrine prophylaxis trial

The study of AL versus a multivitamin preparation will be a two-arm randomised open label comparative study. Laboratory assessments of malaria infection at baseline and day 28 post forest will be performed blind to treatment allocation and incidence of clinical cases during follow-up will be recorded.

Activities/outcomes

The main activity proposed is an *in vivo* clinical assessment of prophylaxis to prevent malaria in 4400 participant episodes in 50 villages in Stung Treng Province, Cambodia. The subjects will be randomized in a one-to-one ratio between the ACT AL and a multivitamin preparation with no antimalarial activity.

The study site has been chosen based on current information on incidence of malaria, known predominance of malaria among forest goers, presence of an established clinical research programme and feasibility to perform the proposed research activities.

Efficacy of AL ACT will be assessed through follow up visits 28 days (+/-7 days) after returning from the forest upon completing each course of prophylaxis when temperature, symptom questionnaires, brief physical examinations, and malaria parasite PCR, and, in selected individuals, parasite genetics will be performed. Episodes of confirmed clinical malaria among study participants at any time point between enrolment and follow-up will also be recorded.

All the organisations in this collaboration will work closely with local counterparts including the National Malaria Control Programmes (NMCPs), non-governmental and other relevant organisations. Training is an integral part of this collaborative working relationship, and the building of local research capacity is an essential component of all research plans.

All research-related activities, from study design, planning, implementation through to analysis and writing of reports will be performed jointly with local counterparts. Both on-the-job training and formal training will be provided when needed, in particular for Good Clinical Practice (GCP) skills.

The close interaction between WHO and its regional offices will ensure that new knowledge is disseminated efficiently and effectively throughout the region.

METHODS AND ANALYSIS

Objectives

Primary Objective

To compare the efficacy of the ACT artemether-lumefantrine versus a multivitamin preparation as defined by the 28-day PCR parasite positivity rate and incidence of confirmed clinical malaria of any species.

Secondary Objectives

1. To compare the efficacy of the ACT artemether-lumefantrine versus a multivitamin preparation as defined by the 28-day, 56-day and 84-day PCR parasite positivity rate and incidence of confirmed clinical malaria for each species.
2. To quantify the impact of the ACT artemether-lumefantrine as prophylaxis for forest goers on overall malaria transmission using mathematical modelling.
3. To assess the impact of artemether-lumefantrine prophylaxis on the spread of genetic markers of artemisinin (such as *Kelch13* mutations) and partner drug resistance.

For peer review only

4. To obtain data on the place of residence, work, recent travel history and risk behaviours of forest goers in order to improve the understanding of high risk groups, locations of malaria transmission and possible routes spread of malaria and artemisinin resistance.
5. To explore the duration, location and purpose of individual forest visits.
6. To obtain detailed data and GPS mapping on a subset of participants and their peers relating to the behaviours and risk factors associated with malaria infection in order to improve understanding of local malaria transmission among forest goers.
7. To determine the prevalence of asymptomatic Plasmodium infections in high risk populations at varying seasonal time points.
8. To determine the prevalence of other infectious diseases that affect the study population.

Trial Design

Study sites

The study will take place at up to 50 villages in selected malaria endemic districts in Stung Treng Province, Cambodia. As the malaria situation in this area is dynamic, the villages will be identified prior to the start of the trial from analysis of up to date malaria incidence from passive surveillance collected by the Cambodia National Center for Parasitology Entomology and Malaria Control. The rationale for choosing these areas include high forest cover and ongoing malaria transmission among forest goers.

Summary of trial design

An open-label randomised trial among forest goers comparing the ACT AL with a multivitamin with no antimalarial activity to evaluate the efficacy of prophylaxis, and to better understand high risk groups and locations of malaria transmission.

Study duration

The recruitment phase of the study is expected to last 12 months. Training and community sensitization will precede study execution for 3 months. Data management and analysis, sample analysis (PCR, parasite genetics), mathematical modelling and report writing are expected to take about 5 months. The total time to complete the study will be about 20 months.

Primary and secondary endpoints

Co-primary Endpoints

1. 28-day PCR positivity rate of Plasmodium infections of any species.
2. Proportion of participants with confirmed clinical malaria of any species reported between day 0 and day 28

Secondary Endpoints

1. 28-day, 56-day and 84-day PCR Plasmodium positivity rate for each Plasmodium species
2. Proportion of participants with confirmed malaria reported between day 0 and day 28 for each species
3. Description of epidemiological situation of malaria in the study areas from passive surveillance data.
4. Prevalence of *Kelch13* mutations and other genetic markers of antimalarial drug resistance of known functional significance.

For peer review only - http://bmjopen.bmj.com/site/about/guidelines.xhtml

For peer review only

5. Incidence of adverse events and serious adverse events by study arms during the course of prophylaxis.

6. Data on the place of residence, work, recent travel history and mobile phone use.

7. Detailed data and GPS mapping on a subset of participants and their peers relating to the behaviours and risk factors associated with malaria infection.

8. Overall prevalence of Plasmodium at baseline, stratified by season and risk factors.

9. Day 0, 28, 56 and 84 capillary blood levels of lumefantrine.

10. Prevalence of serological diagnostic markers of other infectious diseases.

Trial Participants

Overall Description of Trial Participants

Male and non-pregnant female participants aged between 16 years and 65 years planning to visit the forest within 72 hours are the target study population. All study participants must meet the applicable inclusion and exclusion criteria.

Inclusion criteria

1. Male or female, adults aged between 16 and 65 years.
2. Planning to travel to the forest within the next 72 hours and stay overnight.
3. Written informed consent.
4. Willingness and ability of the participants to comply with the study protocol for the duration of the study.

Exclusion criteria

1. For females: known pregnancy or breast feeding
2. Participants who have received artemisinin or a derivative or an artemisinin-containing combination therapy (ACT) within the previous 7 days.
3. History of allergy or known contraindication to artemisinins, lumefantrine or multivitamins
4. Documented or claimed history of cardiac conduction problems
5. Severe vomiting or diarrhoea
6. Signs/symptoms of clinical malaria (febrile or history of fever in the previous 24 hours) confirmed by RDT.

Procedures

Study procedures will be performed according to the schedule of assessments (Appendix 1). This will require that participants are followed up every 28 days for up to 3 periods upon completion of a course of prophylaxis.

Informed Consent

Prior to the start of enrollment we will conduct community mobilisation and sensitisation activities in each village community where the trial will recruit participants. During the trial, the participant (or witness if illiterate) must personally sign and date the latest approved version of the informed consent form before any study specific procedures are performed. Written and verbal versions of the participant information and informed consent in the local language will be presented to the participants detailing no less than: the exact nature of the study; the implications and constraints of the protocol; the known side effects and any risks involved in taking part. It will be clearly stated that participation is voluntary and that the participant is free to withdraw from the study at any time for any reason without prejudice to future care, and with no obligation to give the reason for withdrawal.

For peer review only

The participant will be allowed as much time as possible to consider the information and take the opportunity to question the Investigator, or other independent parties to decide whether they will (or allow his/her charge to) participate in the study. Written informed consent will then be obtained by means of participant dated signature or thumb print (if unable to write) and dated signature of the person who presented and obtained the informed consent.

A copy of the signed informed consent document(s) will be given to the participants.

Children aged 16 to <18 years will be required to sign the latest approved version of the written informed assent form in addition to their parent or guardian signing a consent form.

Screening, Eligibility and Baseline Assessments

Participants who present at the participating sites will be screened to assess eligibility. Full consent will be obtained before any enrolment procedures are conducted. It will be made clear from the outset that refusal to participate will not jeopardise subsequent antimalarial treatment (if applicable). A screening log will be kept.

Demographics and Medical History

Basic demographic and epidemiological data (e.g. sex, age, weight, address, bed net use, malaria risk factors, travel history, prior malaria episodes, prior treatment and previous participation in this or previous studies), and a full medical history will be recorded by the study staff.

Physical Examination and Vital Signs

Brief physical examination and vital sign will be conducted by a qualified study team member. Weight and temperature will be documented. A symptom questionnaire will be performed.

Drug history

All prescribed or over-the-counter and traditional antimalarial medications used within the last 7 days will be recorded. Any drug allergies will be recorded.

Clinical malaria

Participants who are screened and are found to be febrile or have a current history of fever will not be enrolled (as per exclusion criteria) but will be tested for malaria and, if positive, given antimalarial treatment by the village malaria worker or local clinic. All this will be done in accordance with the current national malaria treatment guidelines in Cambodia. Individuals treated for malaria in this way will not be enrolled in the study as per the exclusion criteria.

Randomisation and blinding

Participants who fulfil all the inclusion criteria and have none of the exclusion criteria will be randomised 1:1 to one of the two treatment arms according to a randomisation schedule. Randomisation will be in blocks of size that will be determined by the trial statistician and the block size will not be revealed to the investigating team. Allocation will be done by drawing the next sequential numbered opaque envelope (or other equally reliable randomisation administration procedure), which contains the study number and treatment allocation.

The participants will be assigned a study arm through a computer-generated randomisation schedule. Individual, sealed and sequentially numbered envelopes will be provided for each trial site with one envelope per participant, indicating the treatment allocation.

This is an open-label study so the blinding of investigators and participants is not applicable. However, the randomisation procedure allows for adequate drug allocation concealment before envelopes are opened. All laboratory investigations will be performed without knowledge of the treatment allocation.

Blood sampling on study enrolment

On study enrolment, immediately before drug administration, blood will be collected for the following:

- Parasite PCR (up to 1 ml).

For peer review only - http://bmjopen.bmj.com/site/about/guidelines.xhtml

- Storage for later identification of other causes of fever (2ml).

In case of difficulties with venipuncture on enrolment (e.g. due to dehydration, suitably qualified staff not available in the village) or loss of cold chain during transport from remote villages, 3 dried blood spots will be collected on enrolment for PCR and the other sample collected at follow-up.

Study drug administration

Overview PAL drug regimens	
ACT arm	Multivitamin arm
Artemether-lumefantrine x 3 days followed by 1 day per week	Multivitamin x 3 days followed by 1 day per week

Participants will be treated with weight-based doses according to the schedule in Appendix 2.

The study drugs will be administered by trained study staff.

If the participant vomits within half an hour after intake of the antimalarial drugs, the dose will be repeated. If vomiting occurs between half and one hour, half of the dose will be repeated. If vomiting occurs more than one hour after drug administration, no repeat dosing will be done. Repeat doses will be recorded on the CRF. If vomiting within 1 hour occurs more than one time, no repeat dosing is allowed. The participant will then be treated at the discretion of the investigator.

The prophylaxis will start with a 3-day course of twice daily AL. This will be followed by 2 doses 8 hours apart on one day per week during the time that the person is travelling in the forest and for 4 weeks after leaving the forest.

Follow-up

Participants will be asked to return for a follow-up assessment any time from 28 to 35 days after commencing prophylaxis. This will be regardless of the duration of their visit to the forest or the number of times they visited it in that period. At this assessment, they will be interviewed about how long they spent in the forest, where they went, why, who they travelled with and about risk factors for infection. Brief physical examinations, vital sign and symptom questionnaire will be performed. They will also be asked to report any diagnostic tests and/or treatment for malaria during the preceding 28-35 days.

Blood sampling at follow-up

At each follow-up visit, the following blood will taken:

<u>All individuals</u>:

- Parasite PCR (up to 1 ml).

In those from whom sufficient blood could not be collected at baseline:

- Storage for later identification of other causes of fever (2ml).

From minimum 100 individuals:

- Lumefantrine level (0.2 ml)

<u>In those with confirmed clinical malaria at any time point between enrolment and follow-up</u>:

- Dry blood blots (0.4 ml, 3 spots) collected on filter papers for:
    - Parasite DNA genotyping for genetic markers of antimalarial resistance.
    - Parasite whole genome sequencing and barcoding to identify geographical origin of parasites and compare genotypes to identify persistent infections.

For peer review only - http://bmjopen.bmj.com/site/about/guidelines.xhtml

In individuals who are planning to return again to the forest within the following 28 days after the follow up visit, they will be asked to continue their weekly prophylaxis according to the original treatment allocation on enrolment. They will then be asked to return for a second follow-up visit a further 28 to 35 days later when the above procedure will be repeated. This will be repeated one more time. If the person cannot be followed up within the scheduled period, e.g. because they do not return from the forest in time, then they will be followed up at the first opportunity and this will be recorded in the CRF.

Thus individuals may take prophylaxis continuously for a maximum of 3 periods of 28-35 days in the forest plus 4 weeks after returning totaling 112 days. The choice of study medication for each individual will follow the initial assignment on enrolment throughout the follow-up period.

In those who do not declare an intention to return to the forest within 28 days at any follow-up visit, no further follow-up visits will be offered at that time but they will be asked to complete 4 weeks of prophylaxis following their last day in the forest as post-exposure prophylaxis.

Individuals who have been enrolled in the study may be enrolled into the study up to two more times during the 12 months study period only if a minimum period of 28 days (4 weeks) has elapsed following their last dose of prophylaxis. Thus they can be enrolled in the study up to three times. If an individual is enrolled again in this way, they will be re-randomised following the same procedure as enrolment.

For peer review only

Time windows

The time-window for the follow-up visits is 28-35 days. If a participant does not attend, the study team will try to locate the participant and conduct the necessary examinations and tests.

Additional visits

Participants presenting to the village malaria worker, mobile malaria worker or clinic with a fever or other symptoms at any time after enrolment that is not a scheduled study follow-up visit will be assessed and treated by the healthcare workers in the local healthcare system as per routine clinical practice in Cambodia.

On enrolment, participants will be encouraged to attend a village malaria worker or government clinic for the assessment of fever or other symptoms and to report this to the study team as soon as possible. Information on these healthcare encounters including malaria test result and treatment will be recorded in the study CRF.

Clinical Malaria during Follow-up

Participants who have an episode of confirmed clinical malaria at any time after enrolment up to the last follow-up visit and for one month afterwards will have blood taken for parasite genetic analysis.

Blood volumes

The blood volumes for the protocol mandated tests are as follows:

1. PCR: up to 1ml
2. Lumefantrine level: 0.2ml
3. Dried blood spots for parasite genetics: 0.4ml
4. Storage for serology at baseline 2ml

Maximum blood volumes are presented below for adults for the maximum of three periods (84 days) of follow up. The maximum blood volume is the total amount taken if the participants returns for follow-up on 3 consecutive occasions and had all blood samples taken. The maximum blood volume will be approximately 10.2 ml (less than 10% of total blood volume taken over 8 weeks as recommended by WHO- *Bulletin of the World Health Organization 2011:89:46-53)*.

Allowing for the possibility that we may need to repeat blood tests, we may add 10.2 ml to these estimated maximum blood volumes.

Blood samples collected from this study will be stored no longer than 10 years using codes assigned by the study team or their designee(s). Access to research samples will be limited using either a locked room or a locked freezer.

Analysis of blood samples

Parasite PCR

This is required for the primary study objective. Blood samples will be analysed in the Molecular Tropical Medicine Laboratory, Bangkok, Thailand using PCR to identify which individuals have malaria parasites of any species. It is anticipated that results will be available around 3 to 6 months after collecting each sample, thus they will not be used to guide antimalarial treatment at the time of testing. The study teams will be informed which samples were positive for malaria and they will follow-up positive participants to conduct a brief clinical assessment. Any individuals who are symptomatic will be referred to the village malaria worker or clinic for testing and treatment.

Parasite genetic analysis

Blood samples (dried blood spots) for parasite genetic analysis will be obtained and stored from all subjects recruited with subject's consent. In individuals in whom parasites are found by PCR, samples will be processed for parasite genetic analysis. Genetic samples (in the form of dried blood spots or extracted DNA) will be stored (for a maximum of 10 years) at the Molecular Tropical Medicine Laboratory, Bangkok, Thailand. In those with confirmed clinical malaria, parasite genotyping will be performed at the Wellcome Trust Sanger Institute in Hinxton, UK or other suitable laboratory using a set of informative single nucleotide polymorphisms selected from whole genome sequencing. The subject will be asked for consent for this transfer during the initial informed consent process. A material transfer agreement will be in place if required before any samples are shipped. The results of the parasite genotyping will not be reported back to the subjects. This analysis will only be done for those with confirmed clinical malaria as it is anticipated that there will be insufficient genetic material in samples taken from those with asymptomatic infection due to the low parasite burden in these individuals.

Lumefantrine level

Blood samples or lumefantrine level will be taken at follow-up visits from a minimum of 100 randomly selected participants, where logistically possible, to assess adherence with the study drugs. These will be analysed in the Pharmacology Laboratory at MORU in Bangkok, Thailand.

Serology

Among those who specify by written consent, the serology samples will be analysed for diagnostic markers of other infectious diseases.

Study Drug

Artemether-Lumefantrine

Currently available as standard tablets containing 20 mg artemether and 120 mg of lumefantrine, in a fixed-dose combination formulation. It is included in this formulation on the WHO Model List of Essential Medicines [21].

Target dose/range:

The dose of artemether-lumefantrine is administered as a twice daily dose for 3 days for a total of 6 doses (an initial dose, second dose after 8 hours and then twice daily – morning and evening – for the following two days) followed by twice daily once a week according to the treatment schedule in Appendix 2.

Multivitamin

The multivitamin preparation will be HEXA CMP (Chemephand Medical Co., Ltd.) or suitable equivalent alternative administered as a once daily dose using the treatment schedule in Appendix 2. This multivitamin does not contain any compound with antimalarial activity. Its components are: Vitamin-A: 5000 USP units, Vitamin D: 400 USP Units, Ascorbic acid: 75 mg, Thiamine Mononitrate: 2 mg, Riboflavin: 3 mg and Niacin amide: 20 mg.

For peer review only

Storage of Study Drugs

All efforts will be made to store the study drugs in accordance with the manufacturers' recommendations in a secure area. This may be difficult at some sites where air-conditioned storage rooms are not available. The ACT should be stored between 15°C to 30°C (59°F to 86°F).

Where this is not possible and monitored storage conditions do not meet the recommendations, the artemisinin-derivatives and partner drug content of batches of ACT will be retested at the end of the study.

Compliance with Study Drugs

Study drugs will be administered as Directly-Observed-Therapy (DOT) on the first day. Where possible, study drugs will also be administered as DOT on days 2 and 3. Where DOTs is not possible, the participant will be contacted by the study team by telephone or in person to ensure they take the second and third doses of medication and to ensure they follow the correct procedure in case of vomiting. If the participant vomits, and is re-dosed; this will be recorded in the CRF. If vomiting within 1 hour occurs again after retreatment, no repeat dosing is allowed. All drug doses will be recorded in the CRF. To maximise adherence to the study medication, the study will be preceded by a period of community sensitisation and engagement including information sessions on the importance of taking all three doses of medication. The participants will be requested to take each dose with food to maximize absorption of the lumefantrine.

Accountability of the Study Treatment

All movements of study medication will be recorded. Both study medication of individual participant and overall drug accountability records will be kept up to date by the study staff.

Concomitant Medication

Throughout the study, investigators may prescribe concomitant medications or treatments deemed necessary (e.g. antipyretics or anti-emetics) to provide adequate supportive care except for antibiotics with antimalarial activity unless unavoidable (e.g. doxycycline, azithromycin). If these are required, the participants will be kept in the study and this will be noted as a protocol deviation. Anti-emetics should not be prescribed as a prophylaxis if no nausea or vomiting is present.

Antimalarials for symptomatic, confirmed malaria infections will be prescribed as described above. Any medication, other than the study medication taken during the study will be recorded in the CRF.

Epidemiological data on place of residence, work, travel history and malaria risk

In order to have a greater understanding of the possible sites of malaria transmission, and to relate genetic diversity to geographical location, participants will be asked a short set of questions on their place of residence, place of work and their history of travel plus possible risk factors for malaria. This is to obtain a detailed understanding of the behaviours and risk factors for malaria infection. We will collect GPS coordinates of the places of residence of all participants. In a subset of participants, GPS coordinates will be collected for their travel patterns during follow-up including place of work, forests, forest camps, farms or plantations to identify places where their infection may have occurred. The size of this subset will be determined by the availability of GPS devices with the number being limited to 50 participants at any one time. The GPS devices will be offered to unselected trial participants whenever they are available. We will collect all available local malaria treatment records to describe how the study population compares to the overall population who receive treatment for malaria and this will allow us to better understand local malaria epidemiology and transmission patterns. All personal information will be anonymised so that no individual can be identified from their treatment records, through interviews, or from mapping data.

Malaria incidence data

Passive surveillance data from all available sources for the study province collected by the Cambodia National Center for Parasitology Entomology and Malaria Control will be analysed to identify any changes in malaria incidence rate in study villages before, during and after the study where PA prophylaxis was administered compared to non-study villages.

For peer review only - http://bmjopen.bmj.com/site/about/guidelines.xhtml

Enrolled participants who experience an episode of confirmed clinical malaria during follow-up will be linked back to their individual case records to quantify the incidence of clinical malaria in each study arm.

Analysis

PCR for parasites

PCR will be used to identify which individuals have parasites at enrolment (prior to taking the study medicine) and at each follow-up visit and is required for the primary study objective.

Parasite genetics

Parasite DNA will be used for genomic studies including but not limited to parasite species confirmation, microsatellite typing to identify parasite clones and single nucleotide polymorphisms (SNP) typing/whole genome sequencing to generate data for studies of the geographic origins of the parasites.

Lumefantrine levels

Lumefantrine levels will be used to assess adherence in a random sample of study participants.

Serology

The serology sample will be used for anonymized investigation of the prevalence, incidence, association with fever, and risk factors for other common infectious diseases affecting the study population. Samples will be stored for later analysis.

Discontinuation/ Withdrawal of Participants from the Study

Each participant has the right to discontinue the study drug or the study at any time. Data accrued up until the time of discontinuation will be used in the analysis.

In general, the investigators will be required to make every effort to perform the study procedures until completion of follow-up (maximum 3 visits over 84 days), including in the following situations:

- Significant non-compliance with treatment regimen or study requirements
- An adverse event which requires discontinuation of the study medication or results in inability to continue to comply with study procedures
- Disease which requires discontinuation of the study medication or results in inability to continue to comply with study procedures
- Loss to follow up (every attempt should be made to re-contact the participant)

However, the investigator may discontinue participation in the study of a participant if he or she considers it necessary.

In addition, the participants always have the right to withdraw consent in writing or verbally.

The reason for withdrawal or discontinuation, if available, will be recorded in the CRF. If the study drug or participation in the study is discontinued due to an adverse event, the investigator will arrange for follow-up visits at least until the adverse event has resolved or stabilised.

If a participant does become pregnant during participation in the study, they will be withdrawn from the study immediately upon it being reported to the study team. Any pregnancy must be reported to the Principal Investigator within one working day of awareness. The PI must take all reasonable efforts to discover the outcome of the pregnancy and fill out the pregnancy form. If there is a congenital abnormality or a still born baby, this needs to be reported as a serious adverse event.

Source Data

Source documents are original documents, data, and records from which participants' CRF data are obtained. These include, but are not limited to, village malaria and clinic records (from which medical history and previous and concomitant medication may be summarised into the CRF), clinical and office charts, laboratory and pharmacy records, diaries, microfiches, radiographs, and CRFs.

For peer review only - http://bmjopen.bmj.com/site/about/guidelines.xhtml

For peer review only

CRF entries will be considered source data if the CRF is the site of the original recording (e.g., there is no other written or electronic record of data). In this study, the CRF will be used as the source document for most of the data points.

All documents will be stored safely in confidential conditions.

Safety Reporting

This trial will use drugs that have either been registered or evaluated extensively. To add to the evidence base for safety of AL as prophylaxis, we will record and review all Adverse Events (AEs) and Serious Adverse Events (SAEs) that are reported to occur in the study.

A symptom questionnaire will be performed on enrolment and at each subsequent follow-up visit to the health care center, to aid in the identification of adverse events. In addition, enrolled individuals will be encouraged to promptly report any unexpected symptoms or illnesses between follow-up visits to the study team.

The investigator is responsible for the detection and documentation of events meeting the criteria and definition of an adverse event (AE) or serious adverse event (SAE), as provided in this protocol.

All SAEs and AEs will be promptly documented from the moment of drug administration in the study to discontinuation of the participant from study participation. Any events occurring between screening and drug administration will be considered as baseline, preexisting conditions.

All adverse events must be recorded in the AE/SAE CRF. To avoid colloquial expressions, the adverse event should be reported in standard medical terminology. Whenever possible, the adverse event should be evaluated and reported as a diagnosis rather than as individual signs or symptoms. If a definitive diagnosis is not possible, the individual symptoms and signs should be recorded. Whenever possible, the aetiology of the abnormal findings will be documented on the CRF. Any additional relevant laboratory results obtained by the Investigator during the course of this study will be recorded on the CRF.

If the event meets the criteria for “serious”, the SAE must be reported to the PAL-Cambodia safety team within 24 hours from the time that the event was identified. If further data are required, additional documentation can be submitted. All SAEs must be followed until resolution, or until the SAE is deemed permanent or leads to death.

Samples will be shipped for PCR to a molecular laboratory where they will be analysed in batches. Following quality control results will be available approximately 3-6 months from the time of collection. The list of positive tests will be returned to the field sites. If a participant is found to have a plasmodium infection, and has not already received antimalarial treatment subsequent to the sample being collected, then these individuals will be contacted by a local health worker, and if a participant reports fever or illness they will offered appropriate diagnosis and treatment.

Definitions

Adverse Event (AE)

An AE is any undesirable event or clinical deterioration that occurs to a study participant during the course of the study; that is, from the time of administration of study drugs until study ends (i.e., until the follow up visit) whether or not that event is considered related to the study drugs, or to a concomitant drug or procedure: e.g.

- any unfavourable and unintended symptom
- physical sign
- abnormal laboratory result
- an illness

Any new clinical sign or clinical deterioration that occurs between signing the consent form and the administration of study drugs is not an AE. This information will be recorded in the medical records, as a pre-existing condition.

For peer review only

Serious Adverse Event (SAE)

A serious adverse event is an AE that:

- results in death
- is life-threatening i.e. the participant was at risk of death at the time of the AE
- requires in participant hospitalisation or prolongation of existing hospitalisation
- results in persistent or significant disability/incapacity
- is a congenital anomaly/birth defect
- Any other significant medical condition

All of the above criteria apply to the case as a whole and should not be confused with the outcomes of individual reactions/events. More than one of the above criteria can be applicable to the one event. Important medical events that may not be immediately life-threatening or result in death or hospitalisation may be considered an SAE when, based upon appropriate medical judgment, they may jeopardise the participant or require medical or surgical intervention to prevent one of the outcomes listed in the definition above. Examples of such medical events include allergic bronchospasm requiring intensive treatment in an emergency room or at home, blood dyscrasias or convulsions that do not result in hospitalisation, or development of drug dependency or drug abuse.

Reporting Procedures for Serious Adverse Events

All SAEs must be reported by the site investigator to the Study PI and PAL-Cambodia safety and medical monitor, within one day of his or her awareness of the SAE. The SAE report, should be emailed to the email paltrial@tropmedres.ac.

Further reports should be submitted, if required, until the SAE is resolved.

The site investigator must also report the SAEs to the local ethics committee in accordance with local requirements.

Evaluating Adverse Events and Serious Adverse Events

Assessment of Intensity

Each adverse event will be graded according to the Common Terminology Criteria for Adverse Events (CTCAE) Version 5.0 November 2017.

If an adverse event is not listed in the CTCAE table, the Investigator will assess the severity using the following guidelines:

1 = Mild; asymptomatic or mild symptoms; clinical or diagnostic observations only; intervention not indicated.

2 = Moderate; minimal, local or noninvasive intervention indicated; limiting age appropriate instrumental ADL*

3 = Severe or medically significant but not immediately life-threatening; hospitalization or prolongation of hospitalization indicated; disabling; limiting self care ADL**

4 = Life-Threatening consequences; urgent intervention indicated

5 = Death related to AE

Activities of Daily Living (ADL)

*Instrumental ADL refer to preparing meals, shopping for groceries or clothes, using the telephone, managing money, etc.

**Self care ADL refer to bathing, dressing and undressing, feeding self, using the toilet, taking medications, and not bedridden.

Clarification of the difference in meaning between ‘severe’ and ‘serious’

The term “severe” is often used to describe the intensity (severity) of a specific event (as in mild, moderate, or severe myocardial infarction); the event itself, however, may be of relatively minor

For peer review only - http://bmjopen.bmj.com/site/about/guidelines.xhtml

medical significance (such as severe headache). This is not the same as "serious", which is based on the outcome or criteria defined under the serious adverse event definition. An event can be considered serious without being severe if it conforms to the seriousness criteria, similarly severe events that do not conform to the criteria are not necessarily serious. Seriousness (not severity) serves as a guide for defining regulatory reporting obligations.

Assessment of relatedness

The investigator is obligated to assess the relationship between study drug and the occurrence of each AE/SAE using the following categories of relatedness:

1. Definite: clear-cut temporal association
2. Probable: clear-cut temporal association, with improvement upon drug withdrawal, and not reasonably explained by the participant's known clinical state or other aetiology.
3. Possible: less clear temporal association; other aetiologies are possible. (Other possible aetiologies should be recorded on the CRF).
4. Not related: no temporal association with the study drug; assessed as related to other aetiologies such as concomitant medications or conditions, or participant's known clinical state.

The investigator will provide the assessment of causality as per the AE/SAE data collection tool.

Outcome

The investigator will follow-up the AE and SAE until resolution or until no further medically relevant information can be expected. AE and SAE outcome will be classified as follows:

1. Continuing/ongoing
2. Resolved
3. Resolved with sequelae
4. Permanent
5. Fatal

Statistical Considerations

Sample size justification

The target population for this study will be adult Cambodians who work and sleep in the forest (farmers, collect forest goods, hunting, etc.). 2,200 study participant episodes are required in each arm to have sufficient power to detect a statistically significant difference between the treatment arm and a control arm. The estimate of the required sample size is complicated by the scarce data on *P. falciparum* incidence in forest workers. We estimate the required sample size based on our study in forest rangers in Vietnam in 2016 (13) combined with an estimate of the time spent in the forest from ongoing studies in northeast Cambodia. In Viet Nam, we found that 1 forest ranger became infected per 500 person-nights spent in the forest. We estimate conservatively that during each episode, each participant will spend at least 10 days in a high-risk zone. We will need 20 infectious bites per study-arm: 2x20=40 (i.e. irrespective of protection afforded per arm). Risk days/nights needed: 40x500= 20,000. Each participant episode contributes 10 risk days/nights: 20,000/10 = 2,000 episodes required. The assumed loss to follow-up could be as high as 10% =200. Therefore, the total sample required is 2,000+200=2,200 study participant episodes.

Formally, we anticipate that the risk of being Pf positive without receiving prophylaxis will be around 5%. A total of 1,605 participant-episodes per arm are enough to detect a difference of at least 40% in the proportion of episodes with a Pf positive result as defined by the 28-day PCR parasite positivity rate i.e. from 5% positivity in participants without receiving antimalarial prophylaxis (i.e. multivitamin) to 3% positivity in participants receiving artemether-lumefantrine prophylaxis. This has been estimated with 80% power and 5% significance level. However, we also anticipate that we will likely

For peer review only - http://bmjopen.bmj.com/site/about/guidelines.xhtml

observe multiple episodes being recruited into the study that can reduce power of the study if not accounted for. To compensate for the multiple episodes and any losses to follow up, we plan to recruit approximately 600 (i.e. 595) additional episodes in each group on top of the required 1605 single episodes. This gives an additional 27% episodes to account for the multiple episodes and losses to follow up. Thus, the overall sample size will be 4,400 episodes (i.e. 2,200 episodes in the treatment arm and 2,200 episodes in the control arm). The sample size calculations have been performed in Stata version 15.

Statistical Analyses

Analysis of other endpoints will be described in a Statistical Analysis Plan. A brief overview is given below.

Proportions

These will be compared using chi squared or Fisher's exact test, as appropriate. Crude proportions will be calculated with the exact 95% confidence intervals (CI), where relevant.

Continuous data

These will be summarised by medians (IQR, ranges) and means (standard deviations, 95% CIs), as appropriate, and will include the parasite counts and laboratory parameters. Comparisons of continuous data will be assessed using the paired/unpaired t tests or the sign rank/Mann Whitney U tests, as appropriate.

Safety analysis

Safety analyses will be based on the whole population that get administered the study drug. Safety and tolerability of ACT versus multivitamin will be assessed by comparing the frequency (%) of adverse events and serious adverse events, with particular attention to abdominal pain, appetite perturbation, using the Fisher's exact test. Safety data will be presented in tabular and/or graphical format and summarised descriptively. Any clinically relevant abnormalities or values of potential clinically concern will be described. Participants will be analysed according to an intention to treat and a per protocol method where appropriate.

Adverse events

Adverse events will be graded according to Common Terminology Criteria for Adverse Events (CTCAE) Version 5.0 November 2017.

All adverse events that are newly started or increased in intensity after the study drug administration will be reported. AE reports will be generated for all AEs that occurred after study drug administration, until the end of the study.

Direct Access to Source Documents/Data

Direct access will be granted to authorised representatives from the sponsor and host institution, the regulatory authorities, and ethical committee (if applicable), to permit trial-related monitoring and inspections.

Quality Control and Quality Assurance Procedures

The study will be conducted in accordance with the current approved protocol, ICH GCP, any national regulations that may apply to this study and standard operating procedures. The WWARN will be engaged in assuring QA/QC of study execution in collaboration with the MORU Clinical Trials Support Group (CTSG). Their role will include but not be limited to monitoring adherence to SOPs for collection of laboratory specimens and quality checks (curation) of laboratory data according to standard methodologies.

For peer review only

Monitoring

Study sites may have in place a system for internal monitoring. In addition, regular external monitoring of all sites will be performed by the MORU CTSG according to ICH GCP and a Monitoring Plan. Data will be evaluated for compliance with the protocol and accuracy in relation to source documents. The monitors will check whether the clinical trial is conducted and data are generated, documented and reported in compliance with the protocol, GCP and the applicable regulatory requirements. Evaluation of on-site monitoring schemes, such as a reciprocal monitoring scheme, may be undertaken at selected sites by CTSG.

Patient and Public Involvement

During 2018, extensive consultations were held with local authorities, patients, and study communities regarding the design and organization of the trial. This included the district health authorities and Governor's office. The Siem Pang field station conducted malaria treatment studies and as part of these studies interviews and questionnaires were done with patients to better understand the local risk factors for malaria, travel histories, and the nature of forest work.[22] Specific questions on the time spent in forests was asked[23] and the use of medicines in forests, and the willingness to take antimalarial prophylaxis [Tripura et al in preparation]. A review of forest acquired malaria was prepared at the site in collaboration with local partners.[24] The potential importance of antimalarial prophylaxis was identified through understanding of the high risk of malaria infection in local forests and the willingness of participants to take medicine to prevent this. Through conversations with malaria patients treated at the health centre and from monthly meetings with village malaria health workers the design of the study was informed. This supported decisions about: time spent in forests, follow up scheduling, type of sample collection, monitoring of treatment compliance, suitable locations an communities where patients could be enrolled, concerns and questions surrounding adverse events, defining the messaging and rationale in local languages. Recruitment takes place in villages and community leaders and local health workers including village malaria workers are part of the study team. Patients previously enrolled in studies often serve as guides and assistants as they trust the study team and know the local community and as they are often forest workers themselves they know other forest workers like them who may be willing to participate. Conversations were held with patients and local stakeholders regarding feasibility and to ensure that participation in research would be acceptable and not burdensome or interfering with regular activities. From these discussions we adopted an outreach strategy so that follow up can take place without participants needing to travel long distances or to the health centres. Dissemination will take place on several levels: in villages, at district level, at provincial level, and at a national and international level. As part of an ongoing research platform in the district we will communicate results back to study communities at the end of the trial by public meetings. A series of public engagement activities, including dissemination activities, has run alongside the CNM-MORU malaria field studies since 2013.[25]

For peer review only

ETHICS AND DISSEMINATION

Declaration of Helsinki

The Investigator will ensure that this study is conducted in compliance with the current revision of the Declaration of Helsinki (Fortaleza 2013).

ICH Guidelines for Good Clinical Practice

The Investigators will ensure that this study is conducted according to any National Regulations and that it will follow the principles of the ICH Guidelines for Good Clinical Practice.

Approvals

The study protocol and its associated documents will be submitted to the Oxford Tropical Research Ethics Committee (OxTREC) and the appropriate local ethics committees for written approval.

The Investigator will submit and, where necessary, obtain approval from the above parties for all substantial amendments to the original approved documents.

Risks

This study will use drugs that have been studied thoroughly and their toxicities are well described. In general, they are all well tolerated. In the event of any serious or severe adverse event participants will be referred to the local Referral Hospital where best available care will be provided.

Risks of artemether-lumefantrine

The safety of artemether and lumfantrine for treatment of malaria has been evaluated in clinical trials and, post licensing, widespread use for treating malaria in hundreds of millions of patients per year. Reported AL side effects have generally been mild. Reported adverse reactions in clinical trials have been similar or lower in frequency and magnitude to other ACTs. The commonest (>=3%) reported adverse events in clinical studies with AL in adults were headache, anorexia, dizziness and asthenia. AL is not known to cause harmful prolongation of the QTc interval.[26].

Risks of multivitamin

The main side-effects of multivitamin are upset stomach, unpleasant taste or headache which are mild to moderate in nature. Very rarely, these may cause an allergic reaction.

Risk of phlebotomy and finger prick

The primary risks of phlebotomy include local discomfort, occasional bleeding or bruising of the skin at the site of needle puncture, and rarely haematoma or infection. Phlebotomy will be performed by suitably qualified and trained staff using appropriate hygiene measures including gloves and alcohol swabs to clean the skin.

Risk of GPS data

Due to the potential unique nature of the GPS tracking data, it may be possible to identify individuals from their tracks. This will be minimized by the GPS tracking data being kept separately from any personally identifiable information and linked to the data collected on the study CRF only through a unique study code. The GPS tracking data will also be stored anonymously on the tracking device during collection and moved to an encrypted hard drive upon completion of collection.

5.1 Benefits

There are no anticipated direct benefits to the participants in this study. However, knowledge gained from this study is expected to help to assess the efficacy and feasibility of prophylaxis to prevent malaria in forest workers, and to predict its efficacy in reducing overall transmission. The proposed study is a critical step for future use of chemoprophylaxis to protect forest workers in the GMS against malaria.

For peer review only

Alternatives to Study Participation

Participants are able to decline freely participation in this study. If so, they will receive standard care for their malaria (if applicable).

Incentives & Compensation

Study participants will be compensated for time lost from work as a result of trial activities, the cost of local transport to attend for the follow up visits and will receive a per diem to cover the costs of meals on those days. The amounts in monetary terms will be determined by CNM in accordance with local norms.

The study will pay for treatment for drug-related SAEs or other research-related injuries. The study cannot pay for long term care for disability resulting from complications of the illness.

Confidentiality

The trial staff will ensure that the participants' anonymity is maintained. The participants will be identified only by initials and a study number on the CRF and electronic databases. All documents will be stored securely and be accessible to trial staff and authorised personnel only.

Sample Sharing and Storage

Samples collected will be used for the purpose of this study as stated in the protocol and stored for future use no longer than 10 years. Consent will be obtained from participants for sample storage and/or shipment of specific samples to collaborating institutions for investigations that cannot be performed locally. Any proposed plans to use samples other than for those investigations detailed in this protocol will be submitted to the relevant ethics committees prior to any testing. Material transfer agreements will be arranged and signed where appropriate/needed.

Data Handling and Record Keeping

Study data will be recorded on Case Report Forms (CRF) at the study sites and stored in a secure database. Validation checks will be built into the study database to identify missing values, inconsistencies, or invalid data. Additionally, study data will be profiled using statistical software to check for outliers and errors not detected by the database. All tasks related to data management will be carried out in accordance with the study data management plan.

Data sharing

With participant's consent, participant's data and results from blood analyses may be shared with other researchers in the future, in an anonymized form, following the MORU data sharing policy.

Sponsorship and Insurance

The University of Oxford has a specialist insurance policy in place: - Newline Underwriting Management Ltd, at Lloyd's of London – which would operate in the event of any Participant suffering harm as a result of their involvement in the research.

Dissemination Plan

Results will be published in the open access peer-reviewed medical literature. Any data published will protect the identity of the participants. This trial will be registered in a web based protocol registration scheme. All those who have made a substantial contribution will be co-authors on publications. The sites have the right to publish their data individually and to include members of the sponsor's team who have made a significant contribution. There will also publications of pooled data which will be coordinated by the MORU group. All sites will have the opportunity to contribute to these publications.

All the research findings from the programme and from relevant research outside the Programme will be analysed and integrated, and through the WHO Global Malaria Programme will be disseminated to policy makers, National Malaria Control Programmes (NMCPs) and other researchers.

For peer review only

REFERENCES

1. Guerra, C.A., R.W. Snow, and S.I. Hay, *A global assessment of closed forests, deforestation and malaria risk.* Ann Trop Med Parasitol, 2006. **100**(3): p. 189-204.
2. Thanh, P.V., et al., *Epidemiology of forest malaria in Central Vietnam: the hidden parasite reservoir.* Malar J, 2015. **14**: p. 86.
3. Erhart, A., et al., *Epidemiology of forest malaria in central Vietnam: a large scale cross-sectional survey.* Malar J, 2005. **4**: p. 58.
4. Sanh, N.H., et al., *Forest malaria in central Vietnam.* Am J Trop Med Hyg, 2008. **79**(5): p. 652-4.
5. Thang, N.D., et al., *Malaria in central Vietnam: analysis of risk factors by multivariate analysis and classification tree models.* Malar J, 2008. **7**: p. 28.
6. Erhart, A., et al., *Forest malaria in Vietnam: a challenge for control.* Am J Trop Med Hyg, 2004. **70**(2): p. 110-8.
7. Dysoley, L., et al., *Changing patterns of forest malaria among the mobile adult male population in Chumkiri District, Cambodia.* Acta Trop, 2008. **106**(3): p. 207-12.
8. Moore, S.J., et al., *Border malaria in China: knowledge and use of personal protection by minority populations and implications for malaria control: a questionnaire-based survey.* BMC Public Health, 2008. **8**: p. 344.
9. Gryseels, C., et al., *High Mobility and Low Use of Malaria Preventive Measures Among the Jarai Male Youth Along the Cambodia-Vietnam Border.* Am J Trop Med Hyg, 2015. **93**(4): p. 810-8.
10. Grietens, K.P., et al., *Social determinants of long lasting insecticidal hammock use among the Ra-glai ethnic minority in Vietnam: implications for forest malaria control.* PLoS One, 2012. **7**(1): p. e29991.
11. Lengeler, C., *Insecticide-treated bednets and curtains for preventing malaria.* Cochrane Database Syst Rev, 2000(2): p. CD000363.
12. Thang, N.D., et al., *Long-Lasting Insecticidal Hammocks for controlling forest malaria: a community-based trial in a rural area of central Vietnam.* PLoS One, 2009. **4**(10): p. e7369.
13. Son, D.H., et al., *The prevalence, incidence and prevention of Plasmodium falciparum infections in forest rangers in Bu Gia Map National Park, Binh Phuoc province, Vietnam: a pilot study.* Malar J, 2017. **16**(1): p. 444.
14. York, A., *Seasonal malaria chemoprevention in the Sahel.* Lancet Infect Dis, 2017. **17**(6): p. 588.
15. Snow, R.W., *Seasonal Malaria Chemoprevention: An Evolving Research Paradigm.* PLoS Med, 2016. **13**(11): p. e1002176.
16. Tripura, R., et al., *A Controlled Trial of Mass Drug Administration to Interrupt Transmission of Multidrug-Resistant Falciparum Malaria in Cambodian Villages.* Clin Infect Dis, 2018. **67**(6): p. 817-826.
17. Pryce, J. and P. Hine, *Pyronaridine-artesunate for treating uncomplicated Plasmodium falciparum malaria.* Cochrane Database Syst Rev, 2019. **1**: p. CD006404.
18. Imwong, M., et al., *High-throughput ultrasensitive molecular techniques for quantifying low-density malaria parasitemias.* J Clin Microbiol, 2014. **52**(9): p. 3303-9.
19. Imwong, M., et al., *The epidemiology of subclinical malaria infections in South-East Asia: findings from cross-sectional surveys in Thailand-Myanmar border areas, Cambodia, and Vietnam.* Malar J, 2015. **14**: p. 381.
20. Saunders, D.L., et al., *Dihydroartemisinin-piperaquine failure in Cambodia.* N Engl J Med, 2014. **371**(5): p. 484-5.
21. Organization, W.H., *WHO Model List of Essential Medicines*. 2017.
22. Sanann, N., et al., *Forest work and its implications for malaria elimination: a qualitative study.* Malar J, 2019. **18**(1): p. 376.
23. von Seidlein, L., et al., *Novel Approaches to Control Malaria in Forested Areas of Southeast Asia.* Trends Parasitol, 2019. **35**(6): p. 388-398.
24. Nofal, S.D., et al., *How can interventions that target forest-goers be tailored to accelerate malaria elimination in the Greater Mekong Subregion? A systematic review of the qualitative literature.* Malar J, 2019. **18**(1): p. 32.
25. Lim, R., et al., *Drama as a community engagement strategy for malaria in rural Cambodia.* Wellcome Open Res, 2017. **2**: p. 95.

For peer review only - http://bmjopen.bmj.com/site/about/guidelines.xhtml

26. Haeusler, I.L., et al., *The arrhythmogenic cardiotoxicity of the quinoline and structurally related antimalarial drugs: a systematic review.* BMC Med, 2018. **16**(1): p. 200.

For peer review only

AUTHORS' CONTRIBUTIONS

- Richard J Maude, Co-Principal Investigator, designed the study, wrote the protocol.
- Rupam Tripura, contributed to overall study design, edited the protocol.
- Ean Mom, contributed to design of the field study, edited the protocol.
- Meas Sohka, contributed to design of the field study, edited the protocol.
- Thomas J Peto, contributed to overall study design for fieldwork, edited the protocol.
- James J Callery, contributed to overall study design for fieldwork, edited the protocol.
- Mallika Imwong, contributed to study design for PCR analysis, edited the protocol.
- Mehul Dhorda, contributed to study design for sample collection, edited the protocol.
- Joel Tarning, contributed to study design for PK analysis, edited the protocol.
- Mavuto Mukaka, contributed to study design for statistical analysis, edited the protocol.
- Naomia Waithira, designed data management plan, edited the protocol.
- Jaruwan Tubprasert, provided critical review, edited the protocol.
- Oung Soviet, contributed to study design for fieldwork, edited the protcol.
- Lorenz von Seidlein, contributed to overall study design, edited the protocol.
- Siv Sovannaroth, Co-Principal Investigator, designed the study, edited the protocol.

For peer review only

7 FUNDING STATEMENT

This work was supported by The Global Fund to Fight AIDS, Tuberculosis and Malaria grant number B9R03070 and the Wellcome Trust of Great Britain grant number 106698/Z/14/Z.

For peer review only

8 COMPETING INTERESTS STATEMENT

The authors declare no competing interests.

For peer review only

APPENDIX

Schedule of Assessments

TEST/APPLICATION	SCREENING	D0	D1	D2	CCM*	D28-35	CCM*	D56-63	CCM*	D84-91	CCM* up to D112
Informed consent	X										
Demographics		X									
Risk history		X				X		X		X	
Travel history		X				X		X		X	
Medical and drug history		X									
Symptoms questionnaire		X				X		X		X	
Temperature		X									
Weight		X									
Randomisation and assign study ID		X									
AL/multivitamin doses to be given		X	X	X							
Plasmodium PCR		X				X		X		X	
Blood for storage (serology)		X									
Plasmodium genetic analysis (DBS)					X*		X*		X*		X*
*CCM = episode of confirmed clinical malaria between enrolment and follow-up											

For peer review only

Dosing Schedules

Artemether-lumefantrine

Artemether-lumefantrine dosing schedule										
One tablet AL contains 20 mg artemether and 120 mg lumefantrine (Coartem)										
	No. of tablets recommended at approximate timing of dosing									
	Day 1		Day 2		Day 3		Day 8		Weekly	
Weight: Kilogram	0h	8h	24h	36h	48h	60h	168h	176h	336h …	344h …
15 - <25	2	2	2	2	2	2	2	2	2	2
25 - <35	3	3	3	3	3	3	3	3	3	3
≥35	4	4	4	4	4	4	4	4	4	4
Alternative preparation: one tablet AL contains 80 mg artemether and 480 mg lumefantrine (Artefan)										
	No. of tablets recommended at approximate timing of dosing									
	Day 1		Day 2		Day 3		Day 8		Weekly	
Weight: Kilogram	0h	8h	24h	36h	48h	60h	168h	176h	336h …	344h …
≥35	1	1	1	1	1	1	1	1	1	1

7.1.1 Multivitamin

Multivitamin dosing schedule					
One tablet contains Vitamin-A : 5000 USP units<br>Vitamin D: 400 USP Units<br>Ascorbic acid: 75 mg<br>Thiamine Mononitrate: 2 mg<br>Riboflavin: 3 mg<br>Niacin amide: 20 mg<br>Or suitable equivalent alternative					
	No. of tablets recommended at approximate timing of dosing				
	Day 1	Day 2	Day 3	Day 8	Weekly
Weight: Kilogram	0h	24h	48h	168h	336h …
≥15	1	1	1	1	1

BMJ Open

Study Protocol: an open-label individually randomised controlled trial to assess the efficacy of artemether-lumefantrine prophylaxis for malaria among forest goers in Cambodia

Journal:	*BMJ Open*
Manuscript ID	bmjopen-2020-045900.R1
Article Type:	Protocol
Date Submitted by the Author:	10-Mar-2021
Complete List of Authors:	Maude, Richard; Mahidol University, Mahidol-Oxford Tropical Medicine Research Unit, Faculty of Tropical Medicine; University of Oxford, Centre for Tropical Medicine and Global Health, Nuffield Department of Medicine<br>Tripura, Rupam; Mahidol University, Mahidol-Oxford Tropical Medicine Research Unit, Faculty of Tropical Medicine; University of Oxford Centre for Tropical Medicine and Global Health, Nuffield Department of Medicine<br>Ean, Mom; Mahidol University, Mahidol-Oxford Tropical Medicine Research Unit, Faculty of Tropical Medicine<br>Meas, Sokha; Mahidol University, Mahidol-Oxford Tropical Medicine Research Unit, Faculty of Tropical Medicine<br>Peto, Thomas; Mahidol University, Mahidol-Oxford Tropical Medicine Research Unit, Faculty of Tropical Medicine; University of Oxford Centre for Tropical Medicine and Global Health, Nuffield Department of Medicine<br>Callery, James; Mahidol University, Mahidol-Oxford Tropical Medicine Research Unit, Faculty of Tropical Medicine; University of Oxford Centre for Tropical Medicine and Global Health, Nuffield Department of Medicine<br>Imwong, Mallika; Mahidol University, Mahidol-Oxford Tropical Medicine Research Unit, Faculty of Tropical Medicine; Mahidol University, Department of Molecular Tropical Medicine and Genetics, Faculty of Tropical Medicine<br>Vongpromek, Ranitha; Mahidol University, Mahidol-Oxford Tropical Medicine Research Unit, Faculty of Tropical Medicine; WorldWide Antimalarial Resistance Network, Asia Regional Centre<br>Tarning, Joel; Mahidol University, Mahidol-Oxford Tropical Medicine Research Unit, Faculty of Tropical Medicine; University of Oxford, Centre for Tropical Medicine and Global Health, Nuffield Department of Medicine<br>Mukaka, Mavuto; Mahidol University, Mahidol-Oxford Tropical Medicine Research Unit, Faculty of Tropical Medicine; University of Oxford, Centre for Tropical Medicine and Global Health, Nuffield Department of Medicine<br>Waithira, Naomi; Mahidol University, Mahidol-Oxford Tropical Medicine Research Unit, Faculty of Tropical Medicine; University of Oxford, Centre for Tropical Medicine and Global Health, Nuffield Department of Medicine<br>Soviet, Oung; Provincial Health Department<br>von Seidlein, Lorenz; Mahidol University, Mahidol-Oxford Tropical Medicine Research Unit, Faculty of Tropical Medicine; University of Oxford, Centre for Tropical Medicine and Global Health, Nuffield Department of Medicine<br>Sovannaroth, Siv; National Center for Parasitology, Entomology and

	Malaria Control
**Primary Subject Heading**:	Infectious diseases
Secondary Subject Heading:	Global health
Keywords:	Tropical medicine < INFECTIOUS DISEASES, Public health < INFECTIOUS DISEASES, Epidemiology < INFECTIOUS DISEASES

SCHOLARONE™
Manuscripts

I, the Submitting Author has the right to grant and does grant on behalf of all authors of the Work (as defined in the below author licence), an exclusive licence and/or a non-exclusive licence for contributions from authors who are: i) UK Crown employees; ii) where BMJ has agreed a CC-BY licence shall apply, and/or iii) in accordance with the terms applicable for US Federal Government officers or employees acting as part of their official duties; on a worldwide, perpetual, irrevocable, royalty-free basis to BMJ Publishing Group Ltd ("BMJ") its licensees and where the relevant Journal is co-owned by BMJ to the co-owners of the Journal, to publish the Work in this journal and any other BMJ products and to exploit all rights, as set out in our licence.

The Submitting Author accepts and understands that any supply made under these terms is made by BMJ to the Submitting Author unless you are acting as an employee on behalf of your employer or a postgraduate student of an affiliated institution which is paying any applicable article publishing charge ("APC") for Open Access articles. Where the Submitting Author wishes to make the Work available on an Open Access basis (and intends to pay the relevant APC), the terms of reuse of such Open Access shall be governed by a Creative Commons licence – details of these licences and which Creative Commons licence will apply to this Work are set out in our licence referred to above.

Other than as permitted in any relevant BMJ Author's Self Archiving Policies, I confirm this Work has not been accepted for publication elsewhere, is not being considered for publication elsewhere and does not duplicate material already published. I confirm all authors consent to publication of this Work and authorise the granting of this licence.

For peer review only

Title: Study Protocol: an open-label individually randomised controlled trial to assess the efficacy of artemether-lumefantrine prophylaxis for malaria among forest goers in Cambodia

Short title: Study to assess efficacy of artemether-lumefantrine prophylaxis against forest malaria in Cambodia

Authors:

Richard J Maude[1,2,3,4*]

Rupam Tripura[1,2]

Ean Mom[1]

Meas Sohka[1]

Thomas J Peto[1,2]

James J Callery[1,2]

Mallika Imwong[1,5]

Ranitha Vongpromek[1,6]

Joel Tarning[1,2]

Mavuto Mukaka[1,2]

Naomi Waithira[1,2]

Oung Soviet[7]

Lorenz von Seidlein[1,2]

Siv Sovannaroth[8]

1. Mahidol-Oxford Tropical Medicine Research Unit, Faculty of Tropical Medicine, Mahidol University, Bangkok, Thailand
2. Centre for Tropical Medicine and Global Health, Nuffield Department of Medicine, University of Oxford, Oxford, UK
3. Harvard TH Chan School of Public Health, Harvard University, Boston, USA
4. The Open University, Milton Keynes, UK
5. Department of Molecular Tropical Medicine and Genetics, Faculty of Tropical Medicine, Mahidol University, Bangkok, Thailand
6. Worldwide Antimalarial Resistance Network (WWARN), Asia Regional Centre, Bangkok, Thailand
7. Provincial Health Department, Stung Treng, Cambodia
8. National Center for Parasitology, Entomology and Malaria Control, Phnom Penh, Cambodia

*corresponding author: richard@tropmedres.ac

For peer review only

ABSTRACT

Introduction

In the Greater Mekong Subregion (GMS) adults are at highest risk for malaria. The most relevant disease vectors bite during daytime and outdoors which makes forest work a high-risk activity for malaria. The absence of effective vector control strategies and limited periods of exposure during forest visits suggest that chemoprophylaxis could be an appropriate strategy to protect forest workers against malaria.

Methods and Analysis

The protocol describes an open label randomized controlled trial of artemether-lumefantrine (AL) versus multivitamin as prophylaxis against malaria among forest goers aged 16 to 65 years in rural northeast Cambodia. The primary objective is to compare the efficacy of the artemisinin combination therapy (ACT) AL versus a multivitamin preparation as defined by the 28-day PCR parasite positivity rate and incidence of confirmed clinical malaria of any species. The sample size is 2200 patient-episodes of duration 1 month in each arm. The duration of follow-up and prophylaxis for each participant is 1, 2 or 3 consecutive 28 day periods, followed by a further 28 days of post-exposure prophylaxis, depending on whether they continue to visit to the forest. Analysis will be done both by intention-to-treat and per-protocol.

Ethics and dissemination

All participants will provide written, informed consent. Ethical approval was obtained from the Oxford Tropical Research Ethics Committee and the Cambodia National Ethics Committee for Health Research. Results will be disseminated by peer-reviewed open access publication together with open data.

Registration details

https://clinicaltrials.gov/ct2/show/NCT04041973

ARTICLE SUMMARY

Strengths and limitations of the study

1. Malaria is a major health problem for forest workers in Cambodia. Preventing malaria will provide major health as well as socio-economic benefits for participants in the first instance and, if rolled out, for forest workers more broadly.
2. The trial intervention was designed together with the Cambodian government to be potentially implementable depending on the results.
3. Broad engagement with healthcare workers and communities in the study area preceded enrolment and this is continuing throughout.
4. Local healthcare workers and forest goers assist with running the trial including identifying potential participants and supporting follow-up
5. The trial is open label so participants can know which study drug they are taking.
6. The outcomes are dependent on the incidence of malaria during the trial follow-up period.

For peer review only - http://bmjopen.bmj.com/site/about/guidelines.xhtml

LIST OF ABBREVIATIONS

ACT	Artemisinin-based combination therapy
AE	Adverse event
A/L	Artemether-lumefantrine
AQ	Amodiaquine
CNM	National Center for Parasitology, Entomology and Malaria Control
CRF	Case record form
CTSG	Clinical Trials Support Group (MORU)
DHA	Dihydroartemisinin
DNA	Deoxyribonucleic Acid
EDC	Electronic data capture
EDTA	Ethylene-diamine-tetra-acetic acid
G6PD	Glucose-6-phosphate dehydrogenase
GCP	Good Clinical Practice
GPS	Global Positioning System
Hb	Haemoglobin
Hct	Haematocrit
IRS	Indoor residual spraying
LLIN	Long-lasting insecticide treated bednets
MDR1	Multi-Drug Resistance Gene 1
MQ	Mefloquine
MORU	Mahidol-Oxford Research Unit
NMCP	National Malaria Control Programme
PA	Pyronaridine-artesunate
PAL	Prophylaxis with artemether-lumefantrine study
PCR	Polymerase Chain Reaction
QA	Quality Assurance
QC	Quality Control
SAE	Serious Adverse Event
SNP	Single-nucleotide polymorphism
SOP	Standard Operating Procedure
WHO	World Health Organisation
WWARN	Worldwide Antimalarial Resistance Network

For peer review only

INTRODUCTION

In the Greater Mekong Subregion (GMS) adults are at highest risk for malaria. The most relevant disease vectors bite during daytime and outdoors which makes forest work a high-risk activity for malaria. The absence of effective vector control strategies and limited periods of exposure during forest visits suggest that chemoprophylaxis could be an appropriate strategy to protect forest workers against malaria.

In the Greater Mekong Subregion (GMS) a large proportion of malaria transmission occurs in forested areas, which serve as perpetual sources of transmission [1-5]. Studies have demonstrated increased risk of malaria among forest goers, particularly in men of working age [6, 7] although these have largely been restricted to small geographical areas. Protecting forest goers from Plasmodium infections would not only benefit them directly but also people residing around their home. Malaria elimination efforts which do not consider the reinfection risk from forest workers are unlikely to succeed. However, preventing infections in forest workers is a major challenge. The biting rhythm and resting behaviour of *Anopheles dirus* reduces the impact of the two most commonly employed control measures, long-lasting insecticide treated bednets (LLIN) and indoor residual spraying (IRS). Several studies have also demonstrated poor use of personal protection measures against malaria transmission [8-10]. Two factors that increase malaria risk among forest workers are the basic character of overnight forest accommodation [9] and exposure to the Anopheles vectors (e.g. *An. dirus*), which tend to bite outdoors in daytime. LLIN have a high protective efficacy against nocturnal, indoor malaria transmission [11] but are less protective against daytime, outdoor-biting vectors like An. dirus. The improvised housing of forest workers is frequently poorly suited to hanging bed nets.[12] Imaginative interventions such as supplying forest workers with insecticide treated hammocks do not address the biting rhythm and resting behaviour of the vectors and have a disappointing uptake in field studies for a variety of reasons including incompatibility with traditional sleep arrangements at home or in the forest.[10, 12] In the absence of simple, effective, and affordable vector control interventions, providing forest goers with effective antimalarial prophylaxis seems a promising alternative approach to protect them against malaria provided people can be persuaded to take it.[13]

We propose to evaluate the feasibility and protective efficacy of antimalarial prophylaxis during forest work. It has been demonstrated in sub-Saharan Africa that chemoprophylaxis (SMC) of children, the highest risk group for malaria in tropical Africa, can reduce malaria cases by 75%, is cost effective and safe and can be given by community health workers [14, 15]. We propose to provide chemoprophylaxis to forest workers, the population group with the highest malaria risk in the GMS. In the proposed study we compare chemoprophylaxis with an antimalarial drug, artemether-lumefantrine (AL) to a control agent, multivitamins. A recent mass drug administration in Cambodia demonstrated that DHA/piperaquine remains effective to clear low-density, subclinical *P. falciparum* infections, but there are increasing treatment failures of clinical malaria cases [16] and markers of resistance to piperaquine in Cambodia are increasing. Although artesunate-pyronaridine has recently been introduced for treatment in parts of Cambodia, there remain some unresolved concerns about potential liver toxicity[17]. Evidence to date suggests that efficacy of artemether-lumefantrine remains high in Cambodia and is very well tolerated with an excellent toxicity profile and is thus the preferred potential option for prophylaxis by the National Malaria Control Programme. However, it must be taken with fat to maximize absorption. Previously it has been difficult or impossible to detect very low-density Plasmodium infections. It is important to do so as low density and asymptomatic infections are an important source of malaria transmission in Southeast Asia.[18] The availability of more sensitive PCR methods allows us to detect Plasmodium infections with much lower densities [19, 20]. By use of PCR, we will be able to detect a difference in the prevalence of low density, subclinical *Plasmodium* infections between the two study arms in a relatively small sample of study participants and will seek to identify all species of human malaria including falciparum, vivax and knowlesi.

For peer review only - http://bmjopen.bmj.com/site/about/guidelines.xhtml

Chemoprophylaxis of forest workers could protect this high-risk group and could reduce or even interrupt transmission in villages. The highly encouraging results of seasonal malaria chemoprophylaxis (SMC) in selected regions of sub-Saharan Africa provide hope that targeting another high-risk group, forest workers, could reduce malaria transmission in Cambodia and the wider GMS. In sub-Saharan Africa, children remain the main risk group for Plasmodium infections. In SE Asia the main risk group are adults working and sleeping outdoors hence we propose to provide chemoprophylaxis for these adults. A major challenge for this strategy is the choice of an appropriate chemoprophylactic regimen in the GMS. The chemoprophylactic regimen of choice in Africa is sulfadoxine/pyrimethamine (S/P) plus amodiaquine despite high level resistance against the S/P component of the regimen. Similarly, we propose the use of AL, a drug whose efficacy remains high in the GMS, unlike, for example DHA/piperaquine [21]. The proposed study will help to assess the efficacy and feasibility of prophylaxis to prevent malaria in forest workers, help to identify the optimal regimen, and predict its efficacy in reducing overall transmission. The proposed study is a critical step for future use of chemoprophylaxis to protect forest workers in the GMS against malaria.

Proposed activities

Artemether-lumefantrine prophylaxis trial

The study of AL versus a multivitamin preparation will be a two-arm randomised open label comparative study. Laboratory assessments of malaria infection at baseline and day 28 post forest will be performed blind to treatment allocation and incidence of clinical cases during follow-up will be recorded.

Activities/outcomes

The main activity proposed is an *in vivo* clinical assessment of prophylaxis to prevent malaria in 4400 participant episodes in 50 villages in Stung Treng Province, Cambodia. The subjects will be randomized in a one-to-one ratio between the ACT AL and a multivitamin preparation with no antimalarial activity.

The study site has been chosen based on current information on incidence of malaria, known predominance of malaria among forest goers, presence of an established clinical research programme and feasibility to perform the proposed research activities.

Efficacy of AL ACT will be assessed through follow up visits 28 days (+/-7 days) after returning from the forest upon completing each course of prophylaxis when temperature, symptom questionnaires, brief physical examinations, and malaria parasite PCR, and, in selected individuals, parasite genetics will be performed. Episodes of confirmed clinical malaria among study participants at any time point between enrolment and follow-up will also be recorded.

All the organisations in this collaboration will work closely with local counterparts including the National Malaria Control Programmes (NMCPs), non-governmental and other relevant organisations. Training is an integral part of this collaborative working relationship, and the building of local research capacity is an essential component of all research plans.

All research-related activities, from study design, planning, implementation through to analysis and writing of reports will be performed jointly with local counterparts. Both on-the-job training and formal training will be provided when needed, in particular for Good Clinical Practice (GCP) skills.

The close interaction between WHO and its regional offices will ensure that new knowledge is disseminated efficiently and effectively throughout the region.

METHODS AND ANALYSIS

For peer review only - http://bmjopen.bmj.com/site/about/guidelines.xhtml

Objectives

Primary Objective

To compare the efficacy of the ACT artemether-lumefantrine versus a multivitamin preparation as defined by the 28-day PCR parasite positivity rate and incidence of confirmed clinical malaria of any species.

Secondary Objectives

1. To compare the efficacy of the ACT artemether-lumefantrine versus a multivitamin preparation as defined by the 28-day, 56-day and 84-day PCR parasite positivity rate and incidence of confirmed clinical malaria for each species.
2. To quantify the impact of the ACT artemether-lumefantrine as prophylaxis for forest goers on overall malaria transmission using mathematical modelling.
3. To assess the impact of artemether-lumefantrine prophylaxis on the spread of genetic markers of artemisinin (such as *Kelch13* mutations) and partner drug resistance.
4. To obtain data on the place of residence, work, recent travel history and risk behaviours of forest goers in order to improve the understanding of high risk groups, locations of malaria transmission and possible routes spread of malaria and artemisinin resistance.
5. To explore the duration, location and purpose of individual forest visits.
6. To obtain detailed data and GPS mapping on a subset of participants and their peers relating to the behaviours and risk factors associated with malaria infection in order to improve understanding of local malaria transmission among forest goers.
7. To determine the prevalence of asymptomatic Plasmodium infections in high risk populations at varying seasonal time points.
8. To determine the prevalence of other infectious diseases that affect the study population.

Trial Design

Study sites

The study will take place at up to 50 villages in selected malaria endemic districts in Stung Treng Province, Cambodia. As the malaria situation in this area is dynamic, the villages will be identified prior to the start of the trial from analysis of up to date malaria incidence from passive surveillance collected by the Cambodia National Center for Parasitology Entomology and Malaria Control. The rationale for choosing these areas include high forest cover and ongoing malaria transmission among forest goers. Malaria transmission in this area is generally low but varying over time.

Summary of trial design

An open-label randomised trial among forest goers comparing the ACT AL with a multivitamin with no antimalarial activity to evaluate the efficacy of prophylaxis, and to better understand high risk groups and locations of malaria transmission. Follow-up will be for 1-3 consecutive periods of 28 days depending on whether the participant continues to visit the forest.

Study duration

The recruitment phase of the study is expected to last 12 months. Training and community sensitization will precede study execution for 3 months. Data management and analysis, sample analysis (PCR, parasite genetics), mathematical modelling and report writing are expected to take about 5 months. The total time to complete the study will be about 20 months.

For peer review only

Primary and secondary endpoints

Co-primary Endpoints

1. 28-day PCR positivity rate* of Plasmodium infections of any species.
2. Proportion of participants with confirmed clinical malaria of any species reported between day 0 and day 28

Secondary Endpoints

1. 28-day, 56-day and 84-day PCR Plasmodium positivity rate for each Plasmodium species
2. Proportion of participants with confirmed malaria reported between day 0 and day 28 for each species
3. Description of epidemiological situation of malaria in the study areas from passive surveillance data**
4. Prevalence of *Kelch13* mutations and other genetic markers of antimalarial drug resistance of known functional significance.
5. Incidence of adverse events and serious adverse events by study arms during the course of prophylaxis.
6. Data on the place of residence, work, recent travel history and mobile phone use.
7. Detailed data and GPS mapping on a subset of participants and their peers relating to the behaviours and risk factors associated with malaria infection.
8. Overall prevalence of Plasmodium at baseline, stratified by season and risk factors.
9. Day 0, 28, 56 and 84 capillary blood levels of lumefantrine.
10. Prevalence of serological diagnostic markers of other infectious diseases.

*PCR positivity rate as determined from the proportion of blood samples that were PCR positive.

**This will include the number of cases per village and demographics of those cases.

Trial Participants

Overall Description of Trial Participants

Male and non-pregnant female participants aged between 16 years and 65 years planning to visit the forest within 72 hours are the target study population. The upper age limit was chosen as people over 65 years in the study area rarely travel to the forest and are at low risk of malaria. All pregnant women will be excluded as a conservative measure to minimize risk because of insufficient evidence for safety of artemether-lumefantrine in the first trimester together with frequent uncertainty about the stage of pregnancy as well as lack of consensus about the required dose in pregnancy. All study participants must meet the applicable inclusion and exclusion criteria.

Inclusion criteria

1. Male or female, adults aged between 16 and 65 years.
2. Planning to travel to the forest within the next 72 hours and stay overnight.
3. Written informed consent.
4. Willingness and ability of the participants to comply with the study protocol for the duration of the study.

Exclusion criteria

1. For females: known pregnancy or breast feeding

For peer review only - http://bmjopen.bmj.com/site/about/guidelines.xhtml

2. Participants who have received artemisinin or a derivative or an artemisinin-containing combination therapy (ACT) within the previous 7 days.
3. History of allergy or known contraindication to artemisinins, lumefantrine or multivitamins
4. Documented or claimed history of cardiac conduction problems
5. Severe vomiting or diarrhea on the day of screening
6. Signs/symptoms of clinical malaria (febrile or history of fever in the previous 24 hours) confirmed by RDT.

Procedures

Study procedures will be performed according to the schedule of assessments (Appendix 1). This will require that participants are followed up every 28 days for up to 3 periods upon completion of a course of prophylaxis.

Informed Consent

Prior to the start of enrollment we will conduct community mobilisation and sensitisation activities in each village community where the trial will recruit participants. During the trial, the participant (or witness if illiterate) must personally sign and date the latest approved version of the informed consent form before any study specific procedures are performed. Written and verbal versions of the participant information and informed consent in the local language will be presented to the participants detailing no less than: the exact nature of the study; the implications and constraints of the protocol; the known side effects and any risks involved in taking part. It will be clearly stated that participation is voluntary and that the participant is free to withdraw from the study at any time for any reason without prejudice to future care, and with no obligation to give the reason for withdrawal.

The participant will be allowed as much time as possible to consider the information and take the opportunity to question the Investigator, or other independent parties to decide whether they will (or allow his/her charge to) participate in the study. Written informed consent will then be obtained by means of participant dated signature or thumb print (if unable to write) and dated signature of the person who presented and obtained the informed consent.

A copy of the signed informed consent document(s) will be given to the participants.

Children aged 16 to <18 years will be required to sign the latest approved version of the written informed assent form in addition to their parent or guardian signing a consent form.

Screening, Eligibility and Baseline Assessments

Participants who present at the participating sites will be screened to assess eligibility. Full consent will be obtained before any enrolment procedures are conducted. It will be made clear from the outset that refusal to participate will not jeopardise subsequent antimalarial treatment (if applicable). A screening log will be kept. As detailed below, participants may be enrolled a maximum of 3 times during the study period.

Demographics and Medical History

Basic demographic and epidemiological data (e.g. sex, age, weight, address, bed net use, malaria risk factors, travel history, prior malaria episodes, prior treatment and previous participation in this or previous studies), and a full medical history will be recorded by the study staff.

Physical Examination and Vital Signs

Brief physical examination and vital sign will be conducted by a qualified study team member. Weight and temperature will be documented. A symptom questionnaire will be performed.

For peer review only

Drug history

All prescribed or over-the-counter and traditional antimalarial medications used within the last 7 days will be recorded. Any drug allergies will be recorded.

Clinical malaria

Participants who are screened and are found to be febrile or have a current history of fever will not be enrolled (as per exclusion criteria) but will be tested for malaria and, if positive, given antimalarial treatment by the village malaria worker or local clinic. All this will be done in accordance with the current national malaria treatment guidelines in Cambodia. Individuals treated for malaria in this way will not be enrolled in the study as per the exclusion criteria. Such individuals may be enrolled later following recovery provided they meet the inclusion and exclusion criteria.

Randomisation, allocation and blinding

Participants who fulfil all the inclusion criteria and have none of the exclusion criteria will be randomised 1:1 to one of the two treatment arms according to a randomisation schedule. Randomisation will be in permuted blocks of size that will be determined by the trial statistician and the block size will not be revealed to the investigating team. Randomization will be stratified by village and villages combined for the analysis. Allocation will be done by drawing the next sequential numbered opaque envelope (or other equally reliable randomisation administration procedure), which contains the study number and treatment allocation.

The participants will be assigned a study arm through a computer-generated randomisation schedule. Individual, sealed and sequentially numbered envelopes will be provided for each trial site with one envelope per participant, indicating the treatment allocation.

This is an open-label study so the blinding of investigators and participants is not applicable. However, the randomisation procedure allows for adequate drug allocation concealment before envelopes are opened. All laboratory investigations will be performed without knowledge of the treatment allocation.

Blood sampling on study enrolment

On study enrolment, immediately before drug administration, blood will be collected for the following:

- Parasite PCR (up to 1 ml).
- Storage for later identification of other causes of fever (2ml).

In case of difficulties with venipuncture on enrolment (e.g. due to dehydration, suitably qualified staff not available in the village) or loss of cold chain during transport from remote villages, 3 dried blood spots will be collected on enrolment for PCR and the other sample collected at follow-up.

Study drug administration

Overview PAL drug regimens	
ACT arm	Multivitamin arm
Artemether-lumefantrine x 3 days followed by 1 day per week	Multivitamin x 3 days followed by 1 day per week

For peer review only

Participants will be treated with weight-based doses according to the schedule in Appendix 2.

The study drugs will be administered by trained study staff.

If the participant vomits within half an hour after intake of the antimalarial drugs, the dose will be repeated. If vomiting occurs between half and one hour, half of the dose will be repeated. If vomiting occurs more than one hour after drug administration, no repeat dosing will be done. Repeat doses will be recorded on the CRF. If vomiting within 1 hour occurs more than one time, no repeat dosing is allowed. The participant will then be treated at the discretion of the investigator.

The prophylaxis will start with a 3-day course of twice daily AL. This will be followed by 2 doses 8 hours apart on one day per week during the time that the person is travelling in the forest and for 4 weeks after leaving the forest.

Follow-up

Participants will be asked to return for a follow-up assessment any time from 28 to 35 days after commencing prophylaxis. 28 days was chosen as the upper limit of the time from infection to detectable parasites in the blood. This will be regardless of the duration of their visit to the forest or the number of times they visited it in that period. At this assessment, they will be interviewed about how long they spent in the forest, where they went, why, who they travelled with and about risk factors for infection. Brief physical examinations, vital sign and symptom questionnaire will be performed. They will also be asked to report any diagnostic tests and/or treatment for malaria during the preceding 28-35 days.

Blood sampling at follow-up

At each follow-up visit, the following blood will taken:

All individuals:

- Parasite PCR (up to 1 ml).

In those from whom sufficient blood could not be collected at baseline:

- Storage for later identification of other causes of fever (2ml).

From minimum 100 randomly selected individuals:

- Lumefantrine level (0.2 ml)

In those with confirmed clinical malaria at any time point between enrolment and follow-up:

- Dry blood blots (0.4 ml, 3 spots) collected on filter papers for:
    - Parasite PCR and DNA genotyping for genetic markers of antimalarial resistance.
    - Parasite whole genome sequencing and barcoding to identify geographical origin of parasites and compare genotypes to identify persistent infections.

In individuals who are planning to return again to the forest within the following 28 days after the follow up visit, they will be asked to continue their weekly prophylaxis according to the original treatment allocation on enrolment. They will then be asked to return for a second follow-up visit a further 28 to 35 days later when the above procedure will be repeated. This will be repeated one more time. If the person cannot be followed up within the scheduled period, e.g. because they do not return from the forest in time, then they will be followed up at the first opportunity and this will be recorded in the CRF.

For peer review only

Thus individuals may take prophylaxis continuously for a maximum of 3 periods of 28-35 days in the forest plus 4 weeks after returning totaling 112 days. The choice of study medication for each individual will follow the initial assignment on enrolment throughout the follow-up period.

In those who do not declare an intention to return to the forest within 28 days at any follow-up visit, no further follow-up visits will be offered at that time but they will be asked to complete 4 weeks of prophylaxis following their last day in the forest as post-exposure prophylaxis.

Individuals who have been enrolled in the study may be enrolled into the study up to two more times during the 12 months study period only if a minimum period of 28 days (4 weeks) has elapsed following their last dose of prophylaxis. Thus they can be enrolled in the study up to three times. If an individual is enrolled again in this way, they will be re-randomised following the same procedure as enrolment.

Time windows

The time-window for the follow-up visits is 28-35 days. If a participant does not attend, the study team will try to locate the participant and conduct the necessary examinations and tests.

Additional visits

Participants presenting to the village malaria worker, mobile malaria worker or clinic with a fever or other symptoms at any time after enrolment that is not a scheduled study follow-up visit will be assessed and treated by the healthcare workers in the local healthcare system as per routine clinical practice in Cambodia.

On enrolment, participants will be encouraged to attend a village malaria worker or government clinic for the assessment of fever or other symptoms and to report this to the study team as soon as possible. Information on these healthcare encounters including malaria test result and treatment will be recorded in the study CRF.

Clinical Malaria during Follow-up

Participants who have an episode of confirmed clinical malaria at any time after enrolment up to the last follow-up visit and for one month afterwards will have blood taken for parasite genetic analysis. As clinical malaria at follow-up is one of the co-primary outcomes, and the participants and field staff will not be blinded as to study arm, there is potential for bias if, for example, people in the AL arm choose not to attend for a malaria test. However, extensive efforts will made through community engagement and individual counselling to advise participants against this.

Blood volumes

The blood volumes for the protocol mandated tests are as follows:

1. PCR: up to 1ml
2. Lumefantrine level: 0.2ml
3. Dried blood spots for parasite genetics: 0.4ml
4. Storage for serology at baseline 2ml

Maximum blood volumes are presented below for adults for the maximum of three periods (84 days) of follow up. The maximum blood volume is the total amount taken if the participants returns for follow-up on 3 consecutive occasions and had all blood samples taken. The maximum blood volume will be approximately 10.2 ml (less than 10% of total blood volume taken over 8 weeks as recommended by WHO- *Bulletin of the World Health Organization 2011:89:46-53)*.

Allowing for the possibility that we may need to repeat blood tests, we may add 10.2 ml to these estimated maximum blood volumes.

For peer review only

Blood samples collected from this study will be stored no longer than 10 years using codes assigned by the study team or their designee(s). Access to research samples will be limited using either a locked room or a locked freezer.

Analysis of blood samples

Parasite PCR

This is required for the primary study objective. Blood samples will be analysed in the Molecular Tropical Medicine Laboratory, Bangkok, Thailand using PCR to identify which individuals have malaria parasites of any species. It is anticipated that results will be available around 3 to 6 months after collecting each sample, thus they will not be used to guide antimalarial treatment at the time of testing. The study teams will be informed which samples were positive for malaria and they will follow-up positive participants to conduct a brief clinical assessment. Any individuals who are symptomatic will be referred to the village malaria worker or clinic for testing and treatment. The laboratory will be blinded to the study arm of the patient.

Parasite genetic analysis

Blood samples (dried blood spots) for parasite genetic analysis will be obtained and stored from all subjects recruited with subject's consent. In individuals in whom parasites are found by PCR, samples will be processed for parasite genetic analysis. Genetic samples (in the form of dried blood spots or extracted DNA) will be stored (for a maximum of 10 years) at the Molecular Tropical Medicine Laboratory, Bangkok, Thailand. In those with confirmed clinical malaria, parasite genotyping will be performed at the Wellcome Trust Sanger Institute in Hinxton, UK or other suitable laboratory using a set of informative single nucleotide polymorphisms selected from whole genome sequencing. The subject will be asked for consent for this transfer during the initial informed consent process. A material transfer agreement will be in place if required before any samples are shipped. The results of the parasite genotyping will not be reported back to the subjects. This analysis will only be done for those with confirmed clinical malaria as it is anticipated that there will be insufficient genetic material in samples taken from those with asymptomatic infection due to the low parasite burden in these individuals.

Lumefantrine level

Blood samples for lumefantrine level will be taken at follow-up visits from a minimum of 100 randomly selected participants, where logistically possible, to assess adherence with the study drugs. These will be analysed in the Pharmacology Laboratory at MORU in Bangkok, Thailand.

Serology

Among those who specify by written consent, the serology samples will be analysed for diagnostic markers of other infectious diseases.

Study Drug

Artemether-Lumefantrine

Currently available as standard tablets containing 20 mg artemether and 120 mg of lumefantrine, in a fixed-dose combination formulation. It is included in this formulation on the WHO Model List of Essential Medicines [22].

Target dose/range:

The dose of artemether-lumefantrine is administered as a twice daily dose for 3 days for a total of 6 doses (an initial dose, second dose after 8 hours and then twice daily – morning and evening – for the following two days) followed by twice daily once a week according to the treatment schedule in Appendix 2.

For peer review only

Multivitamin

The multivitamin preparation will be HEXA CMP (Chemephand Medical Co., Ltd.) or suitable equivalent alternative administered as a once daily dose using the treatment schedule in Appendix 2. This multivitamin does not contain any compound with antimalarial activity. Its components are: Vitamin-A: 5000 USP units, Vitamin D: 400 USP Units, Ascorbic acid: 75 mg, Thiamine Mononitrate: 2 mg, Riboflavin: 3 mg and Niacin amide: 20 mg. A multivitamin was chosen because a placebo was not available from the manufacturer for this trial. The multivitamin has no effect on malaria is safe, acceptable to the community and is easily available at the study site. Providing a medication to all participants makes it easier to explain the study in a way that is socially acceptable, and has potential to discourage the sharing of study drugs by participants in the two study arms.

Storage of Study Drugs

All efforts will be made to store the study drugs in accordance with the manufacturers' recommendations in a secure area. This may be difficult at some sites where air-conditioned storage rooms are not available. The ACT should be stored between 15°C to 30°C (59°F to 86°F).

Where this is not possible and monitored storage conditions do not meet the recommendations, the artemisinin-derivatives and partner drug content of batches of ACT will be retested at the end of the study.

Compliance with Study Drugs

Study drugs will be administered as Directly-Observed-Therapy (DOT) on the first day. Where possible, study drugs will also be administered as DOT on days 2 and 3. Where DOTs is not possible, the participant will be contacted by the study team by telephone or in person to ensure they take the second and third doses of medication and to ensure they follow the correct procedure in case of vomiting. If the participant vomits, and is re-dosed; this will be recorded in the CRF. If vomiting within 1 hour occurs again after retreatment, no repeat dosing is allowed. All drug doses will be recorded in the CRF. To maximise adherence to the study medication, the study will be preceded by a period of community sensitisation and engagement including information sessions on the importance of taking all three doses of medication. The participants will be requested to take each dose with food to maximize absorption of the lumefantrine.

Accountability of the Study Treatment

All movements of study medication will be recorded. Both study medication of individual participant and overall drug accountability records will be kept up to date by the study staff.

Concomitant Medication

Throughout the study, investigators may prescribe concomitant medications or treatments deemed necessary (e.g. antipyretics or anti-emetics) to provide adequate supportive care except for antibiotics with antimalarial activity unless unavoidable (e.g. doxycycline, azithromycin). If these are required, the participants will be kept in the study and this will be noted as a protocol deviation. Anti-emetics should not be prescribed as a prophylaxis if no nausea or vomiting is present.

Antimalarials for symptomatic, confirmed malaria infections will be prescribed as described above. Any medication, other than the study medication taken during the study will be recorded in the CRF.

Epidemiological data on place of residence, work, travel history and malaria risk

In order to have a greater understanding of the possible sites of malaria transmission, and to relate genetic diversity to geographical location, participants will be asked a short set of questions on their place of residence, place of work and their history of travel plus possible risk factors for malaria. This is to obtain a detailed understanding of the behaviours and risk factors for malaria infection. We will collect GPS coordinates of the places of residence of all participants. In a subset of participants, GPS coordinates will be collected for their travel patterns during follow-up including

For peer review only - http://bmjopen.bmj.com/site/about/guidelines.xhtml

place of work, forests, forest camps, farms or plantations to identify places where their infection may have occurred. The size of this subset will be determined by the availability of GPS devices with the number being limited to 50 participants at any one time. The GPS devices will be offered to unselected consecutive trial participants whenever they are available, being returned upon completion of follow-up for that individual. We will collect all available local malaria treatment records to describe how the study population compares to the overall population who receive treatment for malaria and this will allow us to better understand local malaria epidemiology and transmission patterns. All personal information will be anonymised so that no individual can be identified from their treatment records, through interviews, or from mapping data.

Malaria incidence data

Passive surveillance data from all available sources for the study province collected by the Cambodia National Center for Parasitology Entomology and Malaria Control will be analysed to identify any changes in malaria incidence rate in study villages before, during and after the study where PA prophylaxis was administered compared to non-study villages.

Enrolled participants who experience an episode of confirmed clinical malaria during follow-up will be linked back to their individual case records to quantify the incidence of clinical malaria in each study arm.

Analysis

PCR for parasites

PCR will be used to identify which individuals have parasites at enrolment (prior to taking the study medicine) and at each follow-up visit and is required for the primary study objective.

Parasite genetics

Parasite DNA will be used for genomic studies including but not limited to parasite species confirmation, microsatellite typing to identify parasite clones and single nucleotide polymorphisms (SNP) typing/whole genome sequencing to generate data for studies of the geographic origins of the parasites.

Lumefantrine levels

Lumefantrine levels will be used to assess adherence in a random sample of study participants.

Serology

The serology sample will be used for anonymized investigation of the prevalence, incidence, association with fever, and risk factors for other common infectious diseases affecting the study population. Samples will be stored for later analysis.

Retention, Discontinuation/Withdrawal of Participants from the Study

All efforts will be made to retain as much data as possible. The main strategy that is being re-enforced for data retention includes study staff reminding participants of the upcoming data collection. This was emphasized during management training. However, each participant has the right to discontinue the study drug or the study at any time. Data accrued up until the time of discontinuation will be used in the analysis.

In general, the investigators will be required to make every effort to perform the study procedures until completion of follow-up (maximum 3 visits over 84 days), including in the following situations:

- Significant non-compliance with treatment regimen or study requirements
- An adverse event which requires discontinuation of the study medication or results in inability to continue to comply with study procedures

For peer review only

- Disease which requires discontinuation of the study medication or results in inability to continue to comply with study procedures
- Loss to follow up (every attempt should be made to re-contact the participant)

However, the investigator may discontinue participation in the study of a participant if he or she considers it necessary.

In addition, the participants always have the right to withdraw consent in writing or verbally.

The reason for withdrawal or discontinuation, if available, will be recorded in the CRF. If the study drug or participation in the study is discontinued due to an adverse event, the investigator will arrange for follow-up visits at least until the adverse event has resolved or stabilised.

If a participant does become pregnant during participation in the study, they will be withdrawn from the study immediately upon it being reported to the study team. Any pregnancy must be reported to the Principal Investigator within one working day of awareness. The PI must take all reasonable efforts to discover the outcome of the pregnancy and fill out the pregnancy form. If there is a congenital abnormality or a still born baby, this needs to be reported as a serious adverse event.

Source Data

Source documents are original documents, data, and records from which participants' CRF data are obtained. These include, but are not limited to, village malaria and clinic records (from which medical history and previous and concomitant medication may be summarised into the CRF), clinical and office charts, laboratory and pharmacy records, diaries, microfiches, radiographs, and CRFs.

CRF entries will be considered source data if the CRF is the site of the original recording (e.g., there is no other written or electronic record of data). In this study, the CRF will be used as the source document for most of the data points.

All documents will be stored safely in confidential conditions.

Safety Reporting

This trial will use drugs that have either been registered or evaluated extensively. To add to the evidence base for safety of AL as prophylaxis, we will record and review all Adverse Events (AEs) and Serious Adverse Events (SAEs) that are reported to occur in the study.

A symptom questionnaire will be performed on enrolment and at each subsequent follow-up visit to the health care center, to aid in the identification of adverse events. In addition, enrolled individuals will be encouraged to promptly report any unexpected symptoms or illnesses between follow-up visits to the study team.

The investigator is responsible for the detection and documentation of events meeting the criteria and definition of an adverse event (AE) or serious adverse event (SAE), as provided in this protocol.

All SAEs and AEs will be promptly documented from the moment of drug administration in the study to discontinuation of the participant from study participation. Any events occurring between screening and drug administration will be considered as baseline, preexisting conditions.

All adverse events must be recorded in the AE/SAE CRF. To avoid colloquial expressions, the adverse event should be reported in standard medical terminology. Whenever possible, the adverse event should be evaluated and reported as a diagnosis rather than as individual signs or symptoms. If a definitive diagnosis is not possible, the individual symptoms and signs should be recorded. Whenever possible, the aetiology of the abnormal findings will be documented on the CRF. Any additional relevant laboratory results obtained by the Investigator during the course of this study will be recorded on the CRF.

For peer review only

If the event meets the criteria for "serious", the SAE must be reported to the PAL-Cambodia safety team within 24 hours from the time that the event was identified. If further data are required, additional documentation can be submitted. All SAEs must be followed until resolution, or until the SAE is deemed permanent or leads to death.

Samples will be shipped for PCR to a molecular laboratory where they will be analysed in batches. Following quality control results will be available approximately 3-6 months from the time of collection. The list of positive tests will be returned to the field sites. If a participant is found to have a plasmodium infection, and has not already received antimalarial treatment subsequent to the sample being collected, then these individuals will be contacted by a local health worker, and if a participant reports fever or illness they will offered appropriate diagnosis and treatment.

Definitions

Adverse Event (AE)

An AE is any undesirable event or clinical deterioration that occurs to a study participant during the course of the study; that is, from the time of administration of study drugs until study ends (i.e., until the follow up visit) whether or not that event is considered related to the study drugs, or to a concomitant drug or procedure: e.g.

- any unfavourable and unintended symptom
- physical sign
- abnormal laboratory result
- an illness

Any new clinical sign or clinical deterioration that occurs between signing the consent form and the administration of study drugs is not an AE. This information will be recorded in the medical records, as a pre-existing condition.

Serious Adverse Event (SAE)

A serious adverse event is an AE that:

- results in death
- is life-threatening i.e. the participant was at risk of death at the time of the AE
- requires in participant hospitalisation or prolongation of existing hospitalisation
- results in persistent or significant disability/incapacity
- is a congenital anomaly/birth defect
- Any other significant medical condition

All of the above criteria apply to the case as a whole and should not be confused with the outcomes of individual reactions/events. More than one of the above criteria can be applicable to the one event. Important medical events that may not be immediately life-threatening or result in death or hospitalisation may be considered an SAE when, based upon appropriate medical judgment, they may jeopardise the participant or require medical or surgical intervention to prevent one of the outcomes listed in the definition above. Examples of such medical events include allergic bronchospasm requiring intensive treatment in an emergency room or at home, blood dyscrasias or convulsions that do not result in hospitalisation, or development of drug dependency or drug abuse.

Reporting Procedures for Serious Adverse Events

All SAEs must be reported by the site investigator to the Study PI and PAL-Cambodia safety and medical monitor, within one day of his or her awareness of the SAE. The SAE report, should be emailed to the email paltrial@tropmedres.ac.

For peer review only

Further reports should be submitted, if required, until the SAE is resolved.

The site investigator must also report the SAEs to the local ethics committee in accordance with local requirements.

Evaluating Adverse Events and Serious Adverse Events

Assessment of Intensity

Each adverse event will be graded according to the Common Terminology Criteria for Adverse Events (CTCAE) Version 5.0 November 2017.

If an adverse event is not listed in the CTCAE table, the Investigator will assess the severity using the following guidelines:

1 = Mild; asymptomatic or mild symptoms; clinical or diagnostic observations only; intervention not indicated.

2 = Moderate; minimal, local or noninvasive intervention indicated; limiting age appropriate instrumental ADL*

3 = Severe or medically significant but not immediately life-threatening; hospitalization or prolongation of hospitalization indicated; disabling; limiting self care ADL**

4 = Life-Threatening consequences; urgent intervention indicated

5 = Death related to AE

Activities of Daily Living (ADL)

*Instrumental ADL refer to preparing meals, shopping for groceries or clothes, using the telephone, managing money, etc.

**Self care ADL refer to bathing, dressing and undressing, feeding self, using the toilet, taking

medications, and not bedridden.

Clarification of the difference in meaning between 'severe' and 'serious'

The term "severe" is often used to describe the intensity (severity) of a specific event (as in mild, moderate, or severe myocardial infarction); the event itself, however, may be of relatively minor medical significance (such as severe headache). This is not the same as "serious", which is based on the outcome or criteria defined under the serious adverse event definition. An event can be considered serious without being severe if it conforms to the seriousness criteria, similarly severe events that do not conform to the criteria are not necessarily serious. Seriousness (not severity) serves as a guide for defining regulatory reporting obligations.

Assessment of relatedness

The investigator is obligated to assess the relationship between study drug and the occurrence of each AE/SAE using the following categories of relatedness:

1. Definite: clear-cut temporal association
2. Probable: clear-cut temporal association, with improvement upon drug withdrawal, and not reasonably explained by the participant's known clinical state or other aetiology.
3. Possible: less clear temporal association; other aetiologies are possible. (Other possible aetiologies should be recorded on the CRF).
4. Not related: no temporal association with the study drug; assessed as related to other aetiologies such as concomitant medications or conditions, or participant's known clinical state.

The investigator will provide the assessment of causality as per the AE/SAE data collection tool.

For peer review only

Outcome

The investigator will follow-up the AE and SAE until resolution or until no further medically relevant information can be expected. AE and SAE outcome will be classified as follows:

1. Continuing/ongoing
2. Resolved
3. Resolved with sequelae
4. Permanent
5. Fatal

Statistical Considerations

Sample size justification

The target population for this study will be adult Cambodians who work and sleep in the forest (farmers, collect forest goods, hunting, etc.). 2,200 study participant episodes are required in each arm to have sufficient power to detect a statistically significant difference between the treatment arm and a control arm. An episode is defined as a follow-up period of 28 days with each enrolled individual contributing 1, 2 or 3 episodes. The estimate of the required sample size is complicated by the scarce data on *P. falciparum* incidence in forest workers.

Formally, we anticipate that the risk of being Pf positive without receiving prophylaxis will be around 5%. A total of 1,605 participant-episodes per arm are enough to detect a difference of at least 40% in the proportion of episodes with a Pf positive result as defined by the 28-day PCR parasite positivity rate i.e. from 5% positivity in participants without receiving antimalarial prophylaxis (i.e. multivitamin) to 3% positivity in participants receiving artemether-lumefantrine prophylaxis. This has been estimated with 80% power and 5% significance level. However, we also anticipate that we will likely observe multiple episodes being recruited into the study that can reduce power of the study if not accounted for. To compensate for the multiple episodes and any losses to follow up, we plan to recruit approximately 600 (i.e. 595) additional episodes in each group on top of the required 1605 single episodes. This gives an additional 27% episodes to account for the multiple episodes and losses to follow up. Thus, the overall sample size will be 4,400 episodes (i.e. 2,200 episodes in the treatment arm and 2,200 episodes in the control arm). The sample size calculations have been performed in Stata version 15.

Statistical Analyses

The main analysis strategy for the primary outcome will be the intention-to-treat (ITT) principle followed by the per protocol (PP) analysis. Thus, we will first analyse the ITT population in which all participants recruited in the trial will be included in the analysis according to the randomisation arm irrespective of what they actually got. These ITT analyses will be followed by the analysis of the per protocol (PP) population in which participants who did not adhere to the protocol will be excluded from analysis.

The co-primary endpoints will be analysed as a composite endpoint. For 28-day PCR Plasmodium positivity and parasite positive clinical episode rate analysis, each arm will be summarised using crude proportions and binomial exact 95% confidence intervals. The risk differences in Plasmodium positivity between AL versus Multivitamin will be reported along with the corresponding 95% confidence intervals. Robust standard errors will be used to handle multiple episodes. These analyses will be complemented by the use of the crude Kaplan-Meier estimates of cumulative PCR Plasmodium positivity and parasite positive clinical episode probabilities as recommended by WHO. The incidence of confirmed clinical malaria between day 0 to day 28 analysis will be modeled using the mixed effects Poisson regression model to obtain incidence rate ratios (IRR) comparing AL versus Multivitamin arms. The mixed effects models will take into account the correlation of multiple

For peer review only - http://bmjopen.bmj.com/site/about/guidelines.xhtml

episodes from the same participant. Tests of significance will be performed at 5% significance level. Analysis of all endpoints will be described in detail in a Statistical Analysis Plan finalised prior to locking the database. A brief overview is given below.

Proportions

These will be compared using chi squared or Fisher's exact test, as appropriate. Crude proportions will be calculated with the exact 95% confidence intervals (CI), where relevant.

Continuous data

These will be summarised by medians (IQR, ranges) and means (standard deviations, 95% CIs), as appropriate, and will include the parasite counts and laboratory parameters. Comparisons of continuous data will be assessed using the paired/unpaired t tests or the sign rank/Mann Whitney U tests, as appropriate.

Safety analysis

Safety analyses will be based on the whole population that get administered the study drug. Safety and tolerability of ACT versus multivitamin will be assessed by comparing the frequency (%) of adverse events and serious adverse events, with particular attention to abdominal pain, appetite perturbation, using the Fisher's exact test. Safety data will be presented in tabular and/or graphical format and summarised descriptively. Any clinically relevant abnormalities or values of potential clinically concern will be described. Participants will be analysed according to an intention to treat and a per protocol method where appropriate.

Handling of missing data

For analyses of proportions, missing outcomes will be imputed using plausible values. For example, worst-case scenario may be deemed appropriate and in that case sensitivity analysis will be performed with the best-case scenario. In the ITT Kaplan-Meier/survival analysis, participants who are lost to follow up, or who have Plasmodium reinfections or inconclusive PCR correction, will be censored from the moment of occurrence of one of these events. This survival analysis approach is the best way of handling missing data because participants with partial information are included in the analysis up to the time when they are lost/withdraw from the study.

Adverse events

Adverse events will be graded according to Common Terminology Criteria for Adverse Events (CTCAE) Version 5.0 November 2017.

All adverse events that are newly started or increased in intensity after the study drug administration will be reported. AE reports will be generated for all AEs that occurred after study drug administration, until the end of the study.

Mathematical modelling

The impact of the ACT artemether-lumefantrine as prophylaxis for forest goers on overall malaria transmission will be quantified using mathematical modelling. For this we will develop a population dynamic village level individual-based model of malaria transmission and treatment parameterised with published data, results from the analysis of data from the trial and fitted to surveillance data from the study area.

Direct Access to Source Documents/Data

Direct access will be granted to authorised representatives from the sponsor and host institution, the regulatory authorities, and ethical committee (if applicable), to permit trial-related monitoring and inspections.

For peer review only

Quality Control and Quality Assurance Procedures

The study will be conducted in accordance with the current approved protocol, ICH GCP, any national regulations that may apply to this study and standard operating procedures. The WWARN will be engaged in assuring QA/QC of study execution in collaboration with the MORU Clinical Trials Support Group (CTSG). Their role will include but not be limited to monitoring adherence to SOPs for collection of laboratory specimens and quality checks (curation) of laboratory data according to standard methodologies.

Monitoring

Study sites may have in place a system for internal monitoring. In addition, regular external monitoring of all sites will be performed by the MORU CTSG according to ICH GCP and a Monitoring Plan. Data will be evaluated for compliance with the protocol and accuracy in relation to source documents. The monitors will check whether the clinical trial is conducted and data are generated, documented and reported in compliance with the protocol, GCP and the applicable regulatory requirements. Evaluation of on-site monitoring schemes, such as a reciprocal monitoring scheme, may be undertaken at selected sites by CTSG.

Patient and Public Involvement

During 2018, extensive consultations were held with local authorities, patients, and study communities regarding the design and organization of the trial. This included the district health authorities and Governor's office. The Siem Pang field station conducted malaria treatment studies and as part of these studies interviews and questionnaires were done with patients to better understand the local risk factors for malaria, travel histories, and the nature of forest work.[23] Specific questions on the time spent in forests was asked[24] and the use of medicines in forests, and the willingness to take antimalarial prophylaxis [Tripura et al in preparation]. A review of forest acquired malaria was prepared at the site in collaboration with local partners.[25] The potential importance of antimalarial prophylaxis was identified through understanding of the high risk of malaria infection in local forests and the willingness of participants to take medicine to prevent this. Through conversations with malaria patients treated at the health centre and from monthly meetings with village malaria health workers the design of the study was informed. This supported decisions about: time spent in forests, follow up scheduling, type of sample collection, monitoring of treatment compliance, suitable locations an communities where patients could be enrolled, concerns and questions surrounding adverse events, defining the messaging and rationale in local languages. Recruitment takes place in villages and community leaders and local health workers including village malaria workers are part of the study team. Patients previously enrolled in studies often serve as guides and assistants as they trust the study team and know the local community and as they are often forest workers themselves they know other forest workers like them who may be willing to participate. Conversations were held with patients and local stakeholders regarding feasibility and to ensure that participation in research would be acceptable and not burdensome or interfering with regular activities. From these discussions we adopted an outreach strategy so that follow up can take place without participants needing to travel long distances or to the health centres. Dissemination will take place on several levels: in villages, at district level, at provincial level, and at a national and international level. As part of an ongoing research platform in the district we will communicate results back to study communities at the end of the trial by public meetings. A series of public engagement activities, including dissemination activities, has run alongside the CNM-MORU malaria field studies since 2013.[26]

For peer review only

ETHICS AND DISSEMINATION

Declaration of Helsinki

The Investigator will ensure that this study is conducted in compliance with the current revision of the Declaration of Helsinki (Fortaleza 2013).

ICH Guidelines for Good Clinical Practice

The Investigators will ensure that this study is conducted according to any National Regulations and that it will follow the principles of the ICH Guidelines for Good Clinical Practice.

Approvals

The study protocol and its associated documents will be submitted to the Oxford Tropical Research Ethics Committee (OxTREC) and the appropriate local ethics committees for written approval.

The Investigator will submit and, where necessary, obtain approval from the above parties for all substantial amendments to the original approved documents.

Risks

This study will use drugs that have been studied thoroughly and their toxicities are well described. In general, they are all well tolerated. In the event of any serious or severe adverse event participants will be referred to the local Referral Hospital where best available care will be provided.

Risks of artemether-lumefantrine

The safety of artemether and lumfantrine for treatment of malaria has been evaluated in clinical trials and, post licensing, widespread use for treating malaria in hundreds of millions of patients per year. Reported AL side effects have generally been mild. Reported adverse reactions in clinical trials have been similar or lower in frequency and magnitude to other ACTs. The commonest (>=3%) reported adverse events in clinical studies with AL in adults were headache, anorexia, dizziness and asthenia. AL is not known to cause harmful prolongation of the QTc interval.[27].

Risks of multivitamin

The main side-effects of multivitamin are upset stomach, unpleasant taste or headache which are mild to moderate in nature. Very rarely, these may cause an allergic reaction.

Risk of phlebotomy and finger prick

The primary risks of phlebotomy include local discomfort, occasional bleeding or bruising of the skin at the site of needle puncture, and rarely haematoma or infection. Phlebotomy will be performed by suitably qualified and trained staff using appropriate hygiene measures including gloves and alcohol swabs to clean the skin.

Risk of GPS data

Due to the potential unique nature of the GPS tracking data, it may be possible to identify individuals from their tracks. This will be minimized by the GPS tracking data being kept separately from any personally identifiable information and linked to the data collected on the study CRF only through a unique study code. The GPS tracking data will also be stored anonymously on the tracking device during collection and moved to an encrypted hard drive upon completion of collection.

5.1 Benefits

There are no anticipated direct benefits to the participants in this study. However, knowledge gained from this study is expected to help to assess the efficacy and feasibility of prophylaxis to prevent malaria in forest workers, and to predict its efficacy in reducing overall transmission. The proposed study is a critical step for future use of chemoprophylaxis to protect forest workers in the GMS against malaria.

For peer review only

Alternatives to Study Participation

Participants are able to decline freely participation in this study. If so, they will receive standard care for their malaria (if applicable).

Incentives & Compensation

Study participants will be compensated for time lost from work as a result of trial activities, the cost of local transport to attend for the follow up visits and will receive a per diem to cover the costs of meals on those days. The amounts in monetary terms will be determined by CNM in accordance with local norms.

The study will pay for treatment for drug-related SAEs or other research-related injuries. The study cannot pay for long term care for disability resulting from complications of the illness.

Confidentiality

The trial staff will ensure that the participants' anonymity is maintained. The participants will be identified only by initials and a study number on the CRF and electronic databases. All documents will be stored securely and be accessible to trial staff and authorised personnel only.

Sample Sharing and Storage

Samples collected will be used for the purpose of this study as stated in the protocol and stored for future use no longer than 10 years. Consent will be obtained from participants for sample storage and/or shipment of specific samples to collaborating institutions for investigations that cannot be performed locally. Any proposed plans to use samples other than for those investigations detailed in this protocol will be submitted to the relevant ethics committees prior to any testing. Material transfer agreements will be arranged and signed where appropriate/needed.

Data Handling and Record Keeping

Study data will be recorded on Case Report Forms (CRF) at the study sites and stored in a secure database. Validation checks will be built into the study database to identify missing values, inconsistencies, or invalid data. Additionally, study data will be profiled using statistical software to check for outliers and errors not detected by the database. All tasks related to data management will be carried out in accordance with the study data management plan.

Data sharing

De-identified, individual participant data from this study will be available to researchers whose proposed purpose of use is approved by the data access committee at Mahidol Oxford Tropical Medicine Research Unit. Inquiries or requests for the data may be sent to datasharing@tropmedres.ac. Sponsorship and Insurance

The University of Oxford has a specialist insurance policy in place: - Newline Underwriting Management Ltd, at Lloyd's of London – which would operate in the event of any Participant suffering harm as a result of their involvement in the research.

Dissemination Plan

Results will be published in the open access peer-reviewed medical literature. Any data published will protect the identity of the participants. This trial will be registered in a web based protocol registration scheme. All those who have made a substantial contribution will be co-authors on publications. The sites have the right to publish their data individually and to include members of the sponsor's team who have made a significant contribution. There will also publications of pooled data which will be coordinated by the MORU group. All sites will have the opportunity to contribute to these publications.

For peer review only

All the research findings from the programme and from relevant research outside the Programme will be analysed and integrated, and through the WHO Global Malaria Programme will be disseminated to policy makers, National Malaria Control Programmes (NMCPs) and other researchers.

For peer review only

REFERENCES

1. Guerra, C.A., R.W. Snow, and S.I. Hay, *A global assessment of closed forests, deforestation and malaria risk.* Ann Trop Med Parasitol, 2006. **100**(3): p. 189-204.
2. Thanh, P.V., et al., *Epidemiology of forest malaria in Central Vietnam: the hidden parasite reservoir.* Malar J, 2015. **14**: p. 86.
3. Erhart, A., et al., *Epidemiology of forest malaria in central Vietnam: a large scale cross-sectional survey.* Malar J, 2005. **4**: p. 58.
4. Sanh, N.H., et al., *Forest malaria in central Vietnam.* Am J Trop Med Hyg, 2008. **79**(5): p. 652-4.
5. Thang, N.D., et al., *Malaria in central Vietnam: analysis of risk factors by multivariate analysis and classification tree models.* Malar J, 2008. **7**: p. 28.
6. Erhart, A., et al., *Forest malaria in Vietnam: a challenge for control.* Am J Trop Med Hyg, 2004. **70**(2): p. 110-8.
7. Dysoley, L., et al., *Changing patterns of forest malaria among the mobile adult male population in Chumkiri District, Cambodia.* Acta Trop, 2008. **106**(3): p. 207-12.
8. Moore, S.J., et al., *Border malaria in China: knowledge and use of personal protection by minority populations and implications for malaria control: a questionnaire-based survey.* BMC Public Health, 2008. **8**: p. 344.
9. Gryseels, C., et al., *High Mobility and Low Use of Malaria Preventive Measures Among the Jarai Male Youth Along the Cambodia-Vietnam Border.* Am J Trop Med Hyg, 2015. **93**(4): p. 810-8.
10. Grietens, K.P., et al., *Social determinants of long lasting insecticidal hammock use among the Ra-glai ethnic minority in Vietnam: implications for forest malaria control.* PLoS One, 2012. **7**(1): p. e29991.
11. Lengeler, C., *Insecticide-treated bednets and curtains for preventing malaria.* Cochrane Database Syst Rev, 2000(2): p. CD000363.
12. Thang, N.D., et al., *Long-Lasting Insecticidal Hammocks for controlling forest malaria: a community-based trial in a rural area of central Vietnam.* PLoS One, 2009. **4**(10): p. e7369.
13. Son, D.H., et al., *The prevalence, incidence and prevention of Plasmodium falciparum infections in forest rangers in Bu Gia Map National Park, Binh Phuoc province, Vietnam: a pilot study.* Malar J, 2017. **16**(1): p. 444.
14. York, A., *Seasonal malaria chemoprevention in the Sahel.* Lancet Infect Dis, 2017. **17**(6): p. 588.
15. Snow, R.W., *Seasonal Malaria Chemoprevention: An Evolving Research Paradigm.* PLoS Med, 2016. **13**(11): p. e1002176.
16. Tripura, R., et al., *A Controlled Trial of Mass Drug Administration to Interrupt Transmission of Multidrug-Resistant Falciparum Malaria in Cambodian Villages.* Clin Infect Dis, 2018. **67**(6): p. 817-826.
17. Pryce, J. and P. Hine, *Pyronaridine-artesunate for treating uncomplicated Plasmodium falciparum malaria.* Cochrane Database Syst Rev, 2019. **1**: p. CD006404.
18. Chaumeau, V., et al., *Contribution of Asymptomatic Plasmodium Infections to the Transmission of Malaria in Kayin State, Myanmar.* J Infect Dis, 2019. **219**(9): p. 1499-1509.
19. Imwong, M., et al., *High-throughput ultrasensitive molecular techniques for quantifying low-density malaria parasitemias.* J Clin Microbiol, 2014. **52**(9): p. 3303-9.
20. Imwong, M., et al., *The epidemiology of subclinical malaria infections in South-East Asia: findings from cross-sectional surveys in Thailand-Myanmar border areas, Cambodia, and Vietnam.* Malar J, 2015. **14**: p. 381.
21. Saunders, D.L., et al., *Dihydroartemisinin-piperaquine failure in Cambodia.* N Engl J Med, 2014. **371**(5): p. 484-5.
22. Organization, W.H., *WHO Model List of Essential Medicines*. 2017.
23. Sanann, N., et al., *Forest work and its implications for malaria elimination: a qualitative study.* Malar J, 2019. **18**(1): p. 376.
24. von Seidlein, L., et al., *Novel Approaches to Control Malaria in Forested Areas of Southeast Asia.* Trends Parasitol, 2019. **35**(6): p. 388-398.

For peer review only - http://bmjopen.bmj.com/site/about/guidelines.xhtml

25. Nofal, S.D., et al., *How can interventions that target forest-goers be tailored to accelerate malaria elimination in the Greater Mekong Subregion? A systematic review of the qualitative literature.* Malar J, 2019. **18**(1): p. 32.
26. Lim, R., et al., *Drama as a community engagement strategy for malaria in rural Cambodia.* Wellcome Open Res, 2017. **2**: p. 95.
27. Haeusler, I.L., et al., *The arrhythmogenic cardiotoxicity of the quinoline and structurally related antimalarial drugs: a systematic review.* BMC Med, 2018. **16**(1): p. 200.

For peer review only

AUTHORS' CONTRIBUTIONS

- Richard J Maude, Co-Principal Investigator, designed the study, wrote the protocol.
- Rupam Tripura, contributed to overall study design, edited the protocol.
- Mom Ean, contributed to design of the field data collection, edited the protocol.
- Meas Sohka, contributed to design of the field data collection, edited the protocol.
- Thomas J Peto, contributed to overall study design, edited the protocol.
- James J Callery, contributed to overall study design, edited the protocol.
- Mallika Imwong, contributed to study design for PCR and genetic analysis, edited the protocol.
- Ranitha Vongpromek, contributed to study design for sample collection, edited the protocol.
- Joel Tarning, contributed to study design for the dosing schedule and analysis for lumefantrine levels, edited the protocol.
- Mavuto Mukaka, contributed to study design for randomization, sample size calculation and statistical analysis, edited the protocol.
- Naomia Waithira, designed the data management plan, edited the protocol.
- Oung Soviet, contributed to study design for field data collection, edited the protocol.
- Lorenz von Seidlein, contributed to overall study design, edited the protocol.
- Siv Sovannaroth, Co-Principal Investigator, designed the study, edited the protocol.

For peer review only

7 FUNDING STATEMENT

This work was supported by The Global Fund to Fight AIDS, Tuberculosis and Malaria grant number B9R03070 and the Wellcome Trust of Great Britain grant number 106698/Z/14/Z.

For peer review only

8 COMPETING INTERESTS STATEMENT

The authors declare no competing interests.

For peer review only

APPENDIX 1 Schedule of Assessments

TEST/APPLICATION	SCREENING	D0	D1	D2	CCM*	D28-35	CCM*	D56-63	CCM*	D84-91	CCM* up to D112
Informed consent	X										
Demographics		X									
Risk history		X				X		X		X	
Travel history		X				X		X		X	
Medical and drug history		X									
Symptoms questionnaire		X				X		X		X	
Temperature		X									
Weight		X									
Randomisation and assign study ID		X									
AL/multivitamin doses to be given		X	X	X							
Plasmodium PCR		X				X		X		X	
Blood for storage (serology)		X									
Plasmodium genetic analysis (DBS)					X*		X*		X*		X*

*CCM = episode of confirmed clinical malaria between enrolment and follow-up

For peer review only

APPENDIX 2 Dosing Schedules

Artemether-lumefantrine

Artemether-lumefantrine dosing schedule										
One tablet AL contains 20 mg artemether and 120 mg lumefantrine (Coartem)										
	No. of tablets recommended at approximate timing of dosing									
	Day 1		Day 2		Day 3		Day 8		Weekly	
Weight: Kilogram	0h	8h	24h	36h	48h	60h	168h	176h	336h …	344h …
15 - <25	2	2	2	2	2	2	2	2	2	2
25 - <35	3	3	3	3	3	3	3	3	3	3
≥35	4	4	4	4	4	4	4	4	4	4
Alternative preparation: one tablet AL contains 80 mg artemether and 480 mg lumefantrine (Artefan)										
	No. of tablets recommended at approximate timing of dosing									
	Day 1		Day 2		Day 3		Day 8		Weekly	
Weight: Kilogram	0h	8h	24h	36h	48h	60h	168h	176h	336h …	344h …
≥35	1	1	1	1	1	1	1	1	1	1

7.1.1 Multivitamin

Multivitamin dosing schedule					
One tablet contains Vitamin-A : 5000 USP units<br>Vitamin D: 400 USP Units<br>Ascorbic acid: 75 mg<br>Thiamine Mononitrate: 2 mg<br>Riboflavin: 3 mg<br>Niacin amide: 20 mg<br>Or suitable equivalent alternative					
	No. of tablets recommended at approximate timing of dosing				
	Day 1	Day 2	Day 3	Day 8	Weekly
Weight: Kilogram	0h	24h	48h	168h	336h …
≥15	1	1	1	1	1

view only

BMJ Open

Study Protocol: an open-label individually randomised controlled trial to assess the efficacy of artemether-lumefantrine prophylaxis for malaria among forest goers in Cambodia

Journal:	*BMJ Open*
Manuscript ID	bmjopen-2020-045900.R2
Article Type:	Protocol
Date Submitted by the Author:	27-May-2021

Complete List of Authors:	Maude, Richard; Mahidol University, Mahidol-Oxford Tropical Medicine Research Unit, Faculty of Tropical Medicine; University of Oxford, Centre for Tropical Medicine and Global Health, Nuffield Department of Medicine<br>Tripura, Rupam; Mahidol University, Mahidol-Oxford Tropical Medicine Research Unit, Faculty of Tropical Medicine; University of Oxford Centre for Tropical Medicine and Global Health, Nuffield Department of Medicine<br>Ean, Mom; Mahidol University, Mahidol-Oxford Tropical Medicine Research Unit, Faculty of Tropical Medicine<br>Meas, Sokha; Mahidol University, Mahidol-Oxford Tropical Medicine Research Unit, Faculty of Tropical Medicine<br>Peto, Thomas; Mahidol University, Mahidol-Oxford Tropical Medicine Research Unit, Faculty of Tropical Medicine; University of Oxford Centre for Tropical Medicine and Global Health, Nuffield Department of Medicine<br>Callery, James; Mahidol University, Mahidol-Oxford Tropical Medicine Research Unit, Faculty of Tropical Medicine; University of Oxford Centre for Tropical Medicine and Global Health, Nuffield Department of Medicine<br>Imwong, Mallika; Mahidol University, Mahidol-Oxford Tropical Medicine Research Unit, Faculty of Tropical Medicine; Mahidol University, Department of Molecular Tropical Medicine and Genetics, Faculty of Tropical Medicine<br>Vongpromek, Ranitha; Mahidol University, Mahidol-Oxford Tropical Medicine Research Unit, Faculty of Tropical Medicine; WorldWide Antimalarial Resistance Network, Asia Regional Centre<br>Tarning, Joel; Mahidol University, Mahidol-Oxford Tropical Medicine Research Unit, Faculty of Tropical Medicine; University of Oxford, Centre for Tropical Medicine and Global Health, Nuffield Department of Medicine<br>Mukaka, Mavuto; Mahidol University, Mahidol-Oxford Tropical Medicine Research Unit, Faculty of Tropical Medicine; University of Oxford, Centre for Tropical Medicine and Global Health, Nuffield Department of Medicine<br>Waithira, Naomi; Mahidol University, Mahidol-Oxford Tropical Medicine Research Unit, Faculty of Tropical Medicine; University of Oxford, Centre for Tropical Medicine and Global Health, Nuffield Department of Medicine<br>Soviet, Oung; Provincial Health Department<br>von Seidlein, Lorenz; Mahidol University, Mahidol-Oxford Tropical Medicine Research Unit, Faculty of Tropical Medicine; University of Oxford, Centre for Tropical Medicine and Global Health, Nuffield Department of Medicine<br>Sovannaroth, Siv; National Center for Parasitology, Entomology and

	Malaria Control
**Primary Subject Heading**:	Infectious diseases
Secondary Subject Heading:	Global health
Keywords:	Tropical medicine < INFECTIOUS DISEASES, Public health < INFECTIOUS DISEASES, Epidemiology < INFECTIOUS DISEASES

I, the Submitting Author has the right to grant and does grant on behalf of all authors of the Work (as defined in the below author licence), an exclusive licence and/or a non-exclusive licence for contributions from authors who are: i) UK Crown employees; ii) where BMJ has agreed a CC-BY licence shall apply, and/or iii) in accordance with the terms applicable for US Federal Government officers or employees acting as part of their official duties; on a worldwide, perpetual, irrevocable, royalty-free basis to BMJ Publishing Group Ltd ("BMJ") its licensees and where the relevant Journal is co-owned by BMJ to the co-owners of the Journal, to publish the Work in this journal and any other BMJ products and to exploit all rights, as set out in our licence.

The Submitting Author accepts and understands that any supply made under these terms is made by BMJ to the Submitting Author unless you are acting as an employee on behalf of your employer or a postgraduate student of an affiliated institution which is paying any applicable article publishing charge ("APC") for Open Access articles. Where the Submitting Author wishes to make the Work available on an Open Access basis (and intends to pay the relevant APC), the terms of reuse of such Open Access shall be governed by a Creative Commons licence – details of these licences and which Creative Commons licence will apply to this Work are set out in our licence referred to above.

Other than as permitted in any relevant BMJ Author's Self Archiving Policies, I confirm this Work has not been accepted for publication elsewhere, is not being considered for publication elsewhere and does not duplicate material already published. I confirm all authors consent to publication of this Work and authorise the granting of this licence.

For peer review only

Title: Study Protocol: an open-label individually randomised controlled trial to assess the efficacy of artemether-lumefantrine prophylaxis for malaria among forest goers in Cambodia

Short title: Study to assess efficacy of artemether-lumefantrine prophylaxis against forest malaria in Cambodia

Authors:

Richard J Maude[1,2,3,4*]

Rupam Tripura[1,2]

Ean Mom[1]

Meas Sohka[1]

Thomas J Peto[1,2]

James J Callery[1,2]

Mallika Imwong[1,5]

Ranitha Vongpromek[1,6]

Joel Tarning[1,2]

Mavuto Mukaka[1,2]

Naomi Waithira[1,2]

Oung Soviet[7]

Lorenz von Seidlein[1,2]

Siv Sovannaroth[8]

1. Mahidol-Oxford Tropical Medicine Research Unit, Faculty of Tropical Medicine, Mahidol University, Bangkok, Thailand
2. Centre for Tropical Medicine and Global Health, Nuffield Department of Medicine, University of Oxford, Oxford, UK
3. Harvard TH Chan School of Public Health, Harvard University, Boston, USA
4. The Open University, Milton Keynes, UK
5. Department of Molecular Tropical Medicine and Genetics, Faculty of Tropical Medicine, Mahidol University, Bangkok, Thailand
6. Worldwide Antimalarial Resistance Network (WWARN), Asia Regional Centre, Bangkok, Thailand
7. Provincial Health Department, Stung Treng, Cambodia
8. National Center for Parasitology, Entomology and Malaria Control, Phnom Penh, Cambodia

*corresponding author: richard@tropmedres.ac

For peer review only

ABSTRACT

Introduction

In the Greater Mekong Subregion (GMS) adults are at highest risk for malaria. The most relevant disease vectors bite during daytime and outdoors which makes forest work a high-risk activity for malaria. The absence of effective vector control strategies and limited periods of exposure during forest visits suggest that chemoprophylaxis could be an appropriate strategy to protect forest workers against malaria.

Methods and Analysis

The protocol describes an open label randomized controlled trial of artemether-lumefantrine (AL) versus multivitamin as prophylaxis against malaria among forest goers aged 16 to 65 years in rural northeast Cambodia. The primary objective is to compare the efficacy of the artemisinin combination therapy (ACT) AL versus a multivitamin preparation as defined by the 28-day PCR parasite positivity rate and incidence of confirmed clinical malaria of any species. The sample size is 2200 patient-episodes of duration 1 month in each arm. The duration of follow-up and prophylaxis for each participant is 1, 2 or 3 consecutive 28 day periods, followed by a further 28 days of post-exposure prophylaxis, depending on whether they continue to visit to the forest. Analysis will be done both by intention-to-treat and per-protocol.

Ethics and dissemination

All participants will provide written, informed consent. Ethical approval was obtained from the Oxford Tropical Research Ethics Committee and the Cambodia National Ethics Committee for Health Research. Results will be disseminated by peer-reviewed open access publication together with open data.

Registration details

https://clinicaltrials.gov/ct2/show/NCT04041973 First posted: 1st August 2019.

ARTICLE SUMMARY

Strengths and limitations of the study

1. Malaria is a major health problem for forest workers in Cambodia. Preventing malaria will provide major health as well as socio-economic benefits for participants in the first instance and, if rolled out, for forest workers more broadly.
2. The trial intervention was designed together with the Cambodian government to be potentially implementable depending on the results.
3. Broad engagement with healthcare workers and communities in the study area preceded enrolment and this is continuing throughout with ocal healthcare workers and forest goers assisting with running the trial including identifying potential participants and supporting follow-up
4. The trial is open label so participants can know which study drug they are taking.
5. The outcomes are dependent on the incidence of malaria during the trial follow-up period.

For peer review only - http://bmjopen.bmj.com/site/about/guidelines.xhtml

LIST OF ABBREVIATIONS

ACT	Artemisinin-based combination therapy
AE	Adverse event
A/L	Artemether-lumefantrine
AQ	Amodiaquine
CNM	National Center for Parasitology, Entomology and Malaria Control
CRF	Case record form
CTSG	Clinical Trials Support Group (MORU)
DHA	Dihydroartemisinin
DNA	Deoxyribonucleic Acid
EDC	Electronic data capture
EDTA	Ethylene-diamine-tetra-acetic acid
G6PD	Glucose-6-phosphate dehydrogenase
GCP	Good Clinical Practice
GPS	Global Positioning System
Hb	Haemoglobin
Hct	Haematocrit
IRS	Indoor residual spraying
LLIN	Long-lasting insecticide treated bednets
MDR1	Multi-Drug Resistance Gene 1
MQ	Mefloquine
MORU	Mahidol-Oxford Research Unit
NMCP	National Malaria Control Programme
PA	Pyronaridine-artesunate
PAL	Prophylaxis with artemether-lumefantrine study
PCR	Polymerase Chain Reaction
QA	Quality Assurance
QC	Quality Control
SAE	Serious Adverse Event
SNP	Single-nucleotide polymorphism
SOP	Standard Operating Procedure
WHO	World Health Organisation
WWARN	Worldwide Antimalarial Resistance Network

For peer review only

INTRODUCTION

In the Greater Mekong Subregion (GMS) adults are at highest risk for malaria. The most relevant disease vectors bite during daytime and outdoors which makes forest work a high-risk activity for malaria. The absence of effective vector control strategies and limited periods of exposure during forest visits suggest that chemoprophylaxis could be an appropriate strategy to protect forest workers against malaria.

In the Greater Mekong Subregion (GMS) a large proportion of malaria transmission occurs in forested areas, which serve as perpetual sources of transmission [1-5]. Studies have demonstrated increased risk of malaria among forest goers, particularly in men of working age [6, 7] although these have largely been restricted to small geographical areas. Protecting forest goers from Plasmodium infections would not only benefit them directly but also people residing around their home. Malaria elimination efforts which do not consider the reinfection risk from forest workers are unlikely to succeed. However, preventing infections in forest workers is a major challenge. The biting rhythm and resting behaviour of *Anopheles dirus* reduces the impact of the two most commonly employed control measures, long-lasting insecticide treated bednets (LLIN) and indoor residual spraying (IRS). Several studies have also demonstrated poor use of personal protection measures against malaria transmission [8-10]. Two factors that increase malaria risk among forest workers are the basic character of overnight forest accommodation [9] and exposure to the Anopheles vectors (e.g. *An. dirus*), which tend to bite outdoors in daytime. LLIN have a high protective efficacy against nocturnal, indoor malaria transmission [11] but are less protective against daytime, outdoor-biting vectors like An. dirus. The improvised housing of forest workers is frequently poorly suited to hanging bed nets.[12] Imaginative interventions such as supplying forest workers with insecticide treated hammocks do not address the biting rhythm and resting behaviour of the vectors and have a disappointing uptake in field studies for a variety of reasons including incompatibility with traditional sleep arrangements at home or in the forest.[10, 12] In the absence of simple, effective, and affordable vector control interventions, providing forest goers with effective antimalarial prophylaxis seems a promising alternative approach to protect them against malaria provided people can be persuaded to take it.[13]

We propose to evaluate the feasibility and protective efficacy of antimalarial prophylaxis during forest work. It has been demonstrated in sub-Saharan Africa that chemoprophylaxis (SMC) of children, the highest risk group for malaria in tropical Africa, can reduce malaria cases by 75%, is cost effective and safe and can be given by community health workers [14, 15]. We propose to provide chemoprophylaxis to forest workers, the population group with the highest malaria risk in the GMS. In the proposed study we compare chemoprophylaxis with an antimalarial drug, artemether-lumefantrine (AL) to a control agent, multivitamins. A recent mass drug administration in Cambodia demonstrated that DHA/piperaquine remains effective to clear low-density, subclinical *P. falciparum* infections, but there are increasing treatment failures of clinical malaria cases [16] and markers of resistance to piperaquine in Cambodia are increasing. Although artesunate-pyronaridine has recently been introduced for treatment in parts of Cambodia, there remain some unresolved concerns about potential liver toxicity[17]. Evidence to date suggests that efficacy of artemether-lumefantrine remains high in Cambodia and is very well tolerated with an excellent toxicity profile and is thus the preferred potential option for prophylaxis by the National Malaria Control Programme. However, it must be taken with fat to maximize absorption. Previously it has been difficult or impossible to detect very low-density Plasmodium infections. It is important to do so as low density and asymptomatic infections are an important source of malaria transmission in Southeast Asia.[18] The availability of more sensitive PCR methods allows us to detect Plasmodium infections with much lower densities [19, 20]. By use of PCR, we will be able to detect a difference in the prevalence of low density, subclinical *Plasmodium* infections between the two study arms in a relatively small sample of study participants and will seek to identify all species of human malaria including falciparum, vivax and knowlesi.

For peer review only - http://bmjopen.bmj.com/site/about/guidelines.xhtml

Chemoprophylaxis of forest workers could protect this high-risk group and could reduce or even interrupt transmission in villages. The highly encouraging results of seasonal malaria chemoprophylaxis (SMC) in selected regions of sub-Saharan Africa provide hope that targeting another high-risk group, forest workers, could reduce malaria transmission in Cambodia and the wider GMS. In sub-Saharan Africa, children remain the main risk group for Plasmodium infections. In SE Asia the main risk group are adults working and sleeping outdoors hence we propose to provide chemoprophylaxis for these adults. A major challenge for this strategy is the choice of an appropriate chemoprophylactic regimen in the GMS. The chemoprophylactic regimen of choice in Africa is sulfadoxine/pyrimethamine (S/P) plus amodiaquine despite high level resistance against the S/P component of the regimen. Similarly, we propose the use of AL, a drug whose efficacy remains high in the GMS, unlike, for example DHA/piperaquine [21]. The proposed study will help to assess the efficacy and feasibility of prophylaxis to prevent malaria in forest workers, help to identify the optimal regimen, and predict its efficacy in reducing overall transmission. The proposed study is a critical step for future use of chemoprophylaxis to protect forest workers in the GMS against malaria.

Proposed activities

Artemether-lumefantrine prophylaxis trial

The study of AL versus a multivitamin preparation will be a two-arm randomised open label comparative study. Laboratory assessments of malaria infection at baseline and day 28 post forest will be performed blind to treatment allocation and incidence of clinical cases during follow-up will be recorded.

Activities/outcomes

The main activity proposed is an *in vivo* clinical assessment of prophylaxis to prevent malaria in 4400 participant episodes in 50 villages in Stung Treng Province, Cambodia. The subjects will be randomized in a one-to-one ratio between the ACT AL and a multivitamin preparation with no antimalarial activity.

The study site has been chosen based on current information on incidence of malaria, known predominance of malaria among forest goers, presence of an established clinical research programme and feasibility to perform the proposed research activities.

Efficacy of AL ACT will be assessed through follow up visits 28 days (+/-7 days) after returning from the forest upon completing each course of prophylaxis when temperature, symptom questionnaires, brief physical examinations, and malaria parasite PCR, and, in selected individuals, parasite genetics will be performed. Episodes of confirmed clinical malaria among study participants at any time point between enrolment and follow-up will also be recorded.

All the organisations in this collaboration will work closely with local counterparts including the National Malaria Control Programmes (NMCPs), non-governmental and other relevant organisations. Training is an integral part of this collaborative working relationship, and the building of local research capacity is an essential component of all research plans.

All research-related activities, from study design, planning, implementation through to analysis and writing of reports will be performed jointly with local counterparts. Both on-the-job training and formal training will be provided when needed, in particular for Good Clinical Practice (GCP) skills.

The close interaction between WHO and its regional offices will ensure that new knowledge is disseminated efficiently and effectively throughout the region.

METHODS AND ANALYSIS

For peer review only

Objectives

Primary Objective

To compare the efficacy of the ACT artemether-lumefantrine versus a multivitamin preparation as defined by the 28-day PCR parasite positivity rate and incidence of confirmed clinical malaria of any species.

Secondary Objectives

1. To compare the efficacy of the ACT artemether-lumefantrine versus a multivitamin preparation as defined by the 28-day, 56-day and 84-day PCR parasite positivity rate and incidence of confirmed clinical malaria for each species.
2. To quantify the impact of the ACT artemether-lumefantrine as prophylaxis for forest goers on overall malaria transmission using mathematical modelling.
3. To assess the impact of artemether-lumefantrine prophylaxis on the spread of genetic markers of artemisinin (such as *Kelch13* mutations) and partner drug resistance.
4. To obtain data on the place of residence, work, recent travel history and risk behaviours of forest goers in order to improve the understanding of high risk groups, locations of malaria transmission and possible routes spread of malaria and artemisinin resistance.
5. To explore the duration, location and purpose of individual forest visits.
6. To obtain detailed data and GPS mapping on a subset of participants and their peers relating to the behaviours and risk factors associated with malaria infection in order to improve understanding of local malaria transmission among forest goers.
7. To determine the prevalence of asymptomatic Plasmodium infections in high risk populations at varying seasonal time points.
8. To determine the prevalence of other infectious diseases that affect the study population.

Trial Design

Study sites

The study will take place at up to 50 villages in selected malaria endemic districts in Stung Treng Province, Cambodia. As the malaria situation in this area is dynamic, the villages will be identified prior to the start of the trial from analysis of up to date malaria incidence from passive surveillance collected by the Cambodia National Center for Parasitology Entomology and Malaria Control. The rationale for choosing these areas include high forest cover and ongoing malaria transmission among forest goers. Malaria transmission in this area is generally low but varying over time.

Summary of trial design

An open-label randomised parallel group superiority trial among forest goers comparing the ACT AL with a multivitamin with no antimalarial activity to evaluate the efficacy of prophylaxis, and to better understand high risk groups and locations of malaria transmission. Follow-up will be for 1-3 consecutive periods of 28 days depending on whether the participant continues to visit the forest.

Study duration

The recruitment phase of the study is expected to last 12 months. Training and community sensitization will precede study execution for 3 months. Data management and analysis, sample analysis (PCR, parasite genetics), mathematical modelling and report writing are expected to take about 5 months. The total time to complete the study will be about 20 months.

For peer review only - http://bmjopen.bmj.com/site/about/guidelines.xhtml

Primary and secondary endpoints

Composite Primary Endpoint

1. 28-day PCR positivity rate* of Plasmodium infections of any species and/or
2. Proportion of participants with confirmed clinical malaria of any species reported between day 0 and day 28

Secondary Endpoints

1. 28-day, 56-day and 84-day PCR Plasmodium positivity rate for each Plasmodium species
2. Proportion of participants with confirmed malaria reported between day 0 and day 28 for each species
3. Description of epidemiological situation of malaria in the study areas from passive surveillance data**
4. Prevalence of *Kelch13* mutations and other genetic markers of antimalarial drug resistance of known functional significance.
5. Incidence of adverse events and serious adverse events by study arms during the course of prophylaxis.
6. Data on the place of residence, work, recent travel history and mobile phone use.
7. Detailed data and GPS mapping on a subset of participants and their peers relating to the behaviours and risk factors associated with malaria infection.
8. Overall prevalence of Plasmodium at baseline, stratified by season and risk factors.
9. Day 0, 28, 56 and 84 capillary blood levels of lumefantrine.
10. Prevalence of serological diagnostic markers of other infectious diseases.

*PCR positivity rate as determined from the proportion of blood samples that were PCR positive.

**This will include the number of cases per village and demographics of those cases.

Trial Participants

Overall Description of Trial Participants

Male and non-pregnant female participants aged between 16 years and 65 years planning to visit the forest within 72 hours are the target study population. The upper age limit was chosen as people over 65 years in the study area rarely travel to the forest and are at low risk of malaria. All pregnant women will be excluded as a conservative measure to minimize risk because of insufficient evidence for safety of artemether-lumefantrine in the first trimester together with frequent uncertainty about the stage of pregnancy as well as lack of consensus about the required dose in pregnancy. All study participants must meet the applicable inclusion and exclusion criteria.

Inclusion criteria

1. Male or female, adults aged between 16 and 65 years.
2. Planning to travel to the forest within the next 72 hours and stay overnight.
3. Written informed consent.
4. Willingness and ability of the participants to comply with the study protocol for the duration of the study.

Exclusion criteria

1. For females: known pregnancy or breast feeding

For peer review only

2. Participants who have received artemisinin or a derivative or an artemisinin-containing combination therapy (ACT) within the previous 7 days.
3. History of allergy or known contraindication to artemisinins, lumefantrine or multivitamins
4. Documented or claimed history of cardiac conduction problems
5. Severe vomiting or diarrhoea on the day of screening
6. Signs/symptoms of clinical malaria (febrile or history of fever in the previous 24 hours) confirmed by RDT.

Procedures

Study procedures will be performed according to the schedule of assessments (supplementary file 1). This will require that participants are followed up every 28 days for up to 3 periods upon completion of a course of prophylaxis. Enrollment will be done by trained trial staff.

Informed Consent

Prior to the start of enrollment we will conduct community mobilisation and sensitisation activities in each village community where the trial will recruit participants. During the trial, the participant (or witness if illiterate) must personally sign and date the latest approved version of the informed consent form before any study specific procedures are performed. Written and verbal versions of the participant information and informed consent in the local language will be presented to the participants by trained study staff detailing no less than: the exact nature of the study; the implications and constraints of the protocol; the known side effects and any risks involved in taking part. It will be clearly stated that participation is voluntary and that the participant is free to withdraw from the study at any time for any reason without prejudice to future care, and with no obligation to give the reason for withdrawal.

The participant will be allowed as much time as possible to consider the information and take the opportunity to question the Investigator, or other independent parties to decide whether they will (or allow his/her charge to) participate in the study. Written informed consent will then be obtained by means of participant dated signature or thumb print (if unable to write) and dated signature of the person who presented and obtained the informed consent. Examples of the patient information sheet and consent form for this study are provided in English in supplementary files 2 and 3, respectively.

A copy of the signed informed consent document(s) will be given to the participants.

Children aged 16 to <18 years will be required to sign the latest approved version of the written informed assent form in addition to their parent or guardian signing a consent form.

Screening, Eligibility and Baseline Assessments

Participants who present at the participating sites will be screened to assess eligibility. Full consent will be obtained before any enrolment procedures are conducted. It will be made clear from the outset that refusal to participate will not jeopardise subsequent antimalarial treatment (if applicable). A screening log will be kept. As detailed below, participants may be enrolled a maximum of 3 times during the study period. People that cannot return for follow-up as per the schedule will not be enrolled. Known pregnancy will be identified by self reporting.

Demographics and Medical History

Basic demographic and epidemiological data (e.g. sex, age, weight, address, bed net use, malaria risk factors, travel history, prior malaria episodes, prior treatment and previous participation in this or previous studies), and a full medical history will be recorded by the study staff.

For peer review only - http://bmjopen.bmj.com/site/about/guidelines.xhtml

Physical Examination and Vital Signs

Brief physical examination and vital sign will be conducted by a qualified study team member. Weight and temperature will be documented. A symptom questionnaire will be performed.

Drug history

All prescribed or over-the-counter and traditional antimalarial medications used within the last 7 days will be recorded. Any drug allergies will be recorded.

Clinical malaria

Participants who are screened and are found to be febrile or have a current history of fever will not be enrolled (as per exclusion criteria) but will be tested for malaria and, if positive, given antimalarial treatment by the village malaria worker or local clinic. All this will be done in accordance with the current national malaria treatment guidelines in Cambodia. Individuals treated for malaria in this way will not be enrolled in the study as per the exclusion criteria. Such individuals may be enrolled later following recovery provided they meet the inclusion and exclusion criteria.

Randomisation, allocation and blinding

Participants who fulfil all the inclusion criteria and have none of the exclusion criteria will be randomised 1:1 to one of the two treatment arms according to a randomisation schedule. Randomisation will be in permuted blocks of size that will be determined by the trial statistician and the block size will not be revealed to the investigating team. Randomization will be stratified by village and villages combined for the analysis. Allocation will be done by trained study staff drawing the next sequential numbered opaque envelope (or other equally reliable randomisation administration procedure), which contains the study number and treatment allocation.

The participants will be assigned a study arm through a computer-generated randomisation schedule. Individual, sealed and sequentially numbered envelopes will be provided for each trial site with one envelope per participant, indicating the treatment allocation.

This is an open-label study so the blinding of investigators and participants is not applicable. However, the randomisation procedure allows for adequate drug allocation concealment before envelopes are opened. All laboratory investigations will be performed without knowledge of the treatment allocation.

Blood sampling on study enrolment

On study enrolment, immediately before drug administration, blood will be collected for the following:

- Parasite PCR (up to 1 ml).
- Storage for later identification of other causes of fever (2ml).

In case of difficulties with venipuncture on enrolment (e.g. due to dehydration, suitably qualified staff not available in the village) or loss of cold chain during transport from remote villages, 3 dried blood spots will be collected on enrolment for PCR and the other sample collected at follow-up.

For peer review only

Study drug administration

For peer review only

Overview PAL drug regimens	
ACT arm	Multivitamin arm

For peer review only

Artemether-lumefantrine x 3 days followed by 1 day per week, twice daily	Multivitamin x 3 days followed by 1 day per week, once daily

Participants will be treated with weight-based doses according to the schedule in supplementary file 1.

The study drugs will be administered by trained study staff.

If the participant vomits within half an hour after intake of the antimalarial drugs, the dose will be repeated. If vomiting occurs between half and one hour, half of the dose will be repeated. If vomiting occurs more than one hour after drug administration, no repeat dosing will be done. Repeat doses will be recorded on the CRF. If vomiting within 1 hour occurs more than one time, no repeat dosing is allowed. The participant will then be treated at the discretion of the investigator.

The prophylaxis will start with a 3-day course of twice daily AL. This will be followed by 2 doses 8 hours apart on one day per week during the time that the person is travelling in the forest and for 4 weeks after leaving the forest.

Follow-up

Participants will be asked to return for a follow-up assessment any time from 28 to 35 days after commencing prophylaxis. 28 days was chosen as the upper limit of the time from infection to detectable parasites in the blood. Ongoing studies in the area found the duration of forest visits varied from a day to several weeks, with very few people being away for more than 28 days. This will be regardless of the duration of their visit to the forest or the number of times they visited it in that period. At this assessment, they will be interviewed about how long they spent in the forest, where they went, why, who they travelled with and about risk factors for infection. Brief physical examinations, vital sign and symptom questionnaire will be performed. They will also be asked to report any diagnostic tests and/or treatment for malaria during the preceding 28-35 days.

Blood sampling at follow-up

At each follow-up visit, the following blood will taken:

All individuals:

- Parasite PCR (up to 1 ml).

In those from whom sufficient blood could not be collected at baseline:

- Storage for later identification of other causes of fever (2ml).

From minimum 100 randomly selected individuals:

- Lumefantrine level (0.2 ml)

In those with confirmed clinical malaria at any time point between enrolment and follow-up:

- Dry blood blots (0.4 ml, 3 spots) collected on filter papers for:
  - Parasite PCR and DNA genotyping for genetic markers of antimalarial resistance.
  - Parasite whole genome sequencing and barcoding to identify geographical origin of parasites and compare genotypes to identify persistent infections.

In individuals who are planning to return again to the forest within the following 28 days after the follow up visit, they will be asked to continue their weekly prophylaxis according to the original

For peer review only - http://bmjopen.bmj.com/site/about/guidelines.xhtml

treatment allocation on enrolment. They will then be asked to return for a second follow-up visit a further 28 to 35 days later when the above procedure will be repeated. This will be repeated one more time. If the person cannot be followed up within the scheduled period, e.g. because they do not return from the forest in time, then they will be followed up at the first opportunity and this will be recorded in the CRF.

Thus individuals may take prophylaxis continuously for a maximum of 3 periods of 28-35 days in the forest plus 4 weeks after returning totaling 112 days. The choice of study medication for each individual will follow the initial assignment on enrolment throughout the follow-up period.

In those who do not declare an intention to return to the forest within 28 days at any follow-up visit, no further follow-up visits will be offered at that time but they will be asked to complete 4 weeks of prophylaxis following their last day in the forest as post-exposure prophylaxis.

Individuals who have been enrolled in the study may be enrolled into the study up to two more times during the 12 months study period only if a minimum period of 28 days (4 weeks) has elapsed following their last dose of prophylaxis. Thus they can be enrolled in the study up to three times. If an individual is enrolled again in this way, they will be re-randomised following the same procedure as enrolment. The rationale for this re-enrolment was that malaria transmission and forest travel are seasonal at this location and this allowed detection of malaria positive episodes in people who continue to visit the forest throughout the year whilst minimizing the period of follow-up for the majority of people who visit the forest only during a particular season; in addition, it allowed a wash-out period between episodes of taking prophylaxis.

Time windows

The time-window for the follow-up visits is 28-35 days. If a participant does not attend, the study team will try to locate the participant and conduct the necessary examinations and tests.

Additional visits

Participants presenting to the village malaria worker, mobile malaria worker or clinic with a fever or other symptoms at any time after enrolment that is not a scheduled study follow-up visit will be assessed and treated by the healthcare workers in the local healthcare system as per routine clinical practice in Cambodia.

On enrolment, participants will be encouraged to attend a village malaria worker or government clinic for the assessment of fever or other symptoms and to report this to the study team as soon as possible. Information on these healthcare encounters including malaria test result and treatment will be recorded in the study CRF.

Clinical Malaria during Follow-up

Participants who have an episode of confirmed clinical malaria at any time after enrolment up to the last follow-up visit and for one month afterwards will have blood taken for parasite genetic analysis. As clinical malaria at follow-up is part of the composite primary endpoint, and the participants and field staff will not be blinded as to study arm, there is potential for bias if, for example, people in the AL arm choose not to attend for a malaria test. However, extensive efforts will made through community engagement and individual counselling to advise participants against this.

Blood volumes

The blood volumes for the protocol mandated tests are as follows:

1. PCR: up to 1ml
2. Lumefantrine level: 0.2ml
3. Dried blood spots for parasite genetics: 0.4ml
4. Storage for serology at baseline 2ml

For peer review only - http://bmjopen.bmj.com/site/about/guidelines.xhtml

Maximum blood volumes are presented below for adults for the maximum of three periods (84 days) of follow up. The maximum blood volume is the total amount taken if the participants returns for follow-up on 3 consecutive occasions and had all blood samples taken. The maximum blood volume will be approximately 10.2 ml (less than 10% of total blood volume taken over 8 weeks as recommended by WHO- *Bulletin of the World Health Organization 2011:89:46-53)*.

Allowing for the possibility that we may need to repeat blood tests, we may add 10.2 ml to these estimated maximum blood volumes.

Blood samples collected from this study will be stored no longer than 10 years using codes assigned by the study team or their designee(s). Access to research samples will be limited using either a locked room or a locked freezer.

Analysis of blood samples

Parasite PCR

This is required for the primary study objective. Blood samples will be analysed in the Molecular Tropical Medicine Laboratory, Bangkok, Thailand using PCR to identify which individuals have malaria parasites of any species. It is anticipated that results will be available around 3 to 6 months after collecting each sample, thus they will not be used to guide antimalarial treatment at the time of testing. The study teams will be informed which samples were positive for malaria and they will follow-up positive participants to conduct a brief clinical assessment. Any individuals who are symptomatic will be referred to the village malaria worker or clinic for testing and treatment. The laboratory will be blinded to the study arm of the patient.

Parasite genetic analysis

Blood samples (dried blood spots) for parasite genetic analysis will be obtained and stored from all subjects recruited with subject's consent. In individuals in whom parasites are found by PCR, samples will be processed for parasite genetic analysis. Genetic samples (in the form of dried blood spots or extracted DNA) will be stored (for a maximum of 10 years) at the Molecular Tropical Medicine Laboratory, Bangkok, Thailand. In those with confirmed clinical malaria, parasite genotyping will be performed at the Wellcome Trust Sanger Institute in Hinxton, UK or other suitable laboratory using a set of informative single nucleotide polymorphisms selected from whole genome sequencing. The subject will be asked for consent for this transfer during the initial informed consent process. A material transfer agreement will be in place if required before any samples are shipped. The results of the parasite genotyping will not be reported back to the subjects. This analysis will only be done for those with confirmed clinical malaria as it is anticipated that there will be insufficient genetic material in samples taken from those with asymptomatic infection due to the low parasite burden in these individuals.

Lumefantrine level

Blood samples for lumefantrine level will be taken at follow-up visits from a minimum of 100 randomly selected participants, where logistically possible, to assess adherence with the study drugs. These will be analysed in the Pharmacology Laboratory at MORU in Bangkok, Thailand.

Serology

Among those who specify by written consent, the serology samples will be analysed for diagnostic markers of other infectious diseases.

Study Drug

Artemether-Lumefantrine

Currently available as standard tablets containing 20 mg artemether and 120 mg of lumefantrine, in a fixed-dose combination formulation. It is included in this formulation on the WHO Model List of Essential Medicines [22].

For peer review only

Target dose/range:

The dose of artemether-lumefantrine is administered as a twice daily dose for 3 days for a total of 6 doses (an initial dose, second dose after 8 hours and then twice daily – morning and evening – for the following two days) followed by twice daily once a week according to the treatment schedule in supplementary file 1.

Multivitamin

The multivitamin preparation will be HEXA CMP (Chemephand Medical Co., Ltd.) or suitable equivalent alternative administered as a once daily dose using the treatment schedule in supplementary file 1. This multivitamin does not contain any compound with antimalarial activity. Its components are: Vitamin-A: 5000 USP units, Vitamin D: 400 USP Units, Ascorbic acid: 75 mg, Thiamine Mononitrate: 2 mg, Riboflavin: 3 mg and Niacin amide: 20 mg. A multivitamin was chosen because a placebo was not available from the manufacturer for this trial. The multivitamin has no effect on malaria is safe, acceptable to the community and is easily available at the study site. Providing a medication to all participants makes it easier to explain the study in a way that is socially acceptable, and has potential to discourage the sharing of study drugs by participants in the two study arms.

Storage of Study Drugs

All efforts will be made to store the study drugs in accordance with the manufacturers' recommendations in a secure area. This may be difficult at some sites where air-conditioned storage rooms are not available. The ACT should be stored between 15°C to 30°C (59°F to 86°F).

Where this is not possible and monitored storage conditions do not meet the recommendations, the artemisinin-derivatives and partner drug content of batches of ACT will be retested at the end of the study.

Compliance with Study Drugs

Study drugs will be administered as Directly-Observed-Therapy (DOT) on the first day. Where possible, study drugs will also be administered as DOT on days 2 and 3. Where DOTs is not possible, the participant will be contacted by the study team by telephone or in person to ensure they take the second and third doses of medication and to ensure they follow the correct procedure in case of vomiting. If the participant vomits, and is re-dosed; this will be recorded in the CRF. If vomiting within 1 hour occurs again after retreatment, no repeat dosing is allowed. All drug doses will be recorded in the CRF. To maximise adherence to the study medication, the study will be preceded by a period of community sensitisation and engagement including information sessions on the importance of taking all three doses of medication. The participants will be requested to take each dose with food to maximize absorption of the lumefantrine.

Accountability of the Study Treatment

All movements of study medication will be recorded. Both study medication of individual participant and overall drug accountability records will be kept up to date by the study staff.

Concomitant Medication

Throughout the study, investigators may prescribe concomitant medications or treatments deemed necessary (e.g. antipyretics or anti-emetics) to provide adequate supportive care except for antibiotics with antimalarial activity unless unavoidable (e.g. doxycycline, azithromycin). If these are required, the participants will be kept in the study and this will be noted as a protocol deviation. Anti-emetics should not be prescribed as a prophylaxis if no nausea or vomiting is present.

For peer review only

Antimalarials for symptomatic, confirmed malaria infections will be prescribed as described above. Any medication, other than the study medication taken during the study will be recorded in the CRF.

Epidemiological data on place of residence, work, travel history and malaria risk

In order to have a greater understanding of the possible sites of malaria transmission, and to relate genetic diversity to geographical location, participants will be asked a short set of questions on their place of residence, place of work and their history of travel plus possible risk factors for malaria. This is to obtain a detailed understanding of the behaviours and risk factors for malaria infection. We will collect GPS coordinates of the places of residence of all participants. In a subset of participants, GPS coordinates will be collected for their travel patterns during follow-up including place of work, forests, forest camps, farms or plantations to identify places where their infection may have occurred. The size of this subset will be determined by the availability of GPS devices with the number being limited to 50 participants at any one time. The GPS devices will be offered to unselected consecutive trial participants whenever they are available, being returned upon completion of follow-up for that individual. We will collect all available local malaria treatment records to describe how the study population compares to the overall population who receive treatment for malaria and this will allow us to better understand local malaria epidemiology and transmission patterns. All personal information will be anonymised so that no individual can be identified from their treatment records, through interviews, or from mapping data.

Malaria incidence data

Passive surveillance data from all available sources for the study province collected by the Cambodia National Center for Parasitology Entomology and Malaria Control will be analysed to identify any changes in malaria incidence rate in study villages before, during and after the study where PA prophylaxis was administered compared to non-study villages.

Enrolled participants who experience an episode of confirmed clinical malaria during follow-up will be linked back to their individual case records to quantify the incidence of clinical malaria in each study arm.

Analysis

PCR for parasites

PCR will be used to identify which individuals have parasites at enrolment (prior to taking the study medicine) and at each follow-up visit and is required for the primary study objective.

Parasite genetics

Parasite DNA will be used for genomic studies including but not limited to parasite species confirmation, microsatellite typing to identify parasite clones and single nucleotide polymorphisms (SNP) typing/whole genome sequencing to generate data for studies of the geographic origins of the parasites.

Lumefantrine levels

Lumefantrine levels will be used to assess adherence in a random sample of study participants.

Serology

The serology sample will be used for anonymized investigation of the prevalence, incidence, association with fever, and risk factors for other common infectious diseases affecting the study population. Samples will be stored for later analysis.

Retention, Discontinuation/Withdrawal of Participants from the Study

All efforts will be made to retain as much data as possible. The main strategy that is being re-enforced for data retention includes study staff reminding participants of the upcoming data

For peer review only - http://bmjopen.bmj.com/site/about/guidelines.xhtml

collection. This will be emphasized during management training. However, each participant has the right to discontinue the study drug or the study at any time. Data accrued up until the time of discontinuation will be used in the analysis.

In general, the investigators will be required to make every effort to perform the study procedures until completion of follow-up (maximum 3 visits over 84 days), including in the following situations:

- Significant non-compliance with treatment regimen or study requirements
- An adverse event which requires discontinuation of the study medication or results in inability to continue to comply with study procedures
- Disease which requires discontinuation of the study medication or results in inability to continue to comply with study procedures
- Loss to follow up (every attempt should be made to re-contact the participant)

However, the investigator may discontinue participation in the study of a participant if he or she considers it necessary.

In addition, the participants always have the right to withdraw consent in writing or verbally.

The reason for withdrawal or discontinuation, if available, will be recorded in the CRF. If the study drug or participation in the study is discontinued due to an adverse event, the investigator will arrange for follow-up visits at least until the adverse event has resolved or stabilised.

If a participant does become pregnant during participation in the study, they will be withdrawn from the study immediately upon it being reported to the study team. Any pregnancy must be reported to the Principal Investigator within one working day of awareness. The PI must take all reasonable efforts to discover the outcome of the pregnancy and fill out the pregnancy form. If there is a congenital abnormality or a still born baby, this needs to be reported as a serious adverse event.

Source Data

Source documents are original documents, data, and records from which participants' CRF data are obtained. These include, but are not limited to, village malaria and clinic records (from which medical history and previous and concomitant medication may be summarised into the CRF), clinical and office charts, laboratory and pharmacy records, diaries, microfiches, radiographs, and CRFs.

CRF entries will be considered source data if the CRF is the site of the original recording (e.g., there is no other written or electronic record of data). In this study, the CRF will be used as the source document for most of the data points.

All documents will be stored safely in confidential conditions.

Safety Reporting

This trial will use drugs that have either been registered or evaluated extensively. To add to the evidence base for safety of AL as prophylaxis, we will record and review all Adverse Events (AEs) and Serious Adverse Events (SAEs) that are reported to occur in the study.

A symptom questionnaire will be performed on enrolment and at each subsequent follow-up visit to the health care center, to aid in the identification of adverse events. In addition, enrolled individuals will be encouraged to promptly report any unexpected symptoms or illnesses between follow-up visits to the study team.

The investigator is responsible for the detection and documentation of events meeting the criteria and definition of an adverse event (AE) or serious adverse event (SAE), as provided in this protocol.

All SAEs and AEs will be promptly documented from the moment of drug administration in the study to discontinuation of the participant from study participation. Any events occurring between screening and drug administration will be considered as baseline, preexisting conditions.

For peer review only

All adverse events must be recorded in the AE/SAE CRF. To avoid colloquial expressions, the adverse event should be reported in standard medical terminology. Whenever possible, the adverse event should be evaluated and reported as a diagnosis rather than as individual signs or symptoms. If a definitive diagnosis is not possible, the individual symptoms and signs should be recorded. Whenever possible, the aetiology of the abnormal findings will be documented on the CRF. Any additional relevant laboratory results obtained by the Investigator during the course of this study will be recorded on the CRF.

If the event meets the criteria for "serious", the SAE must be reported to the PAL-Cambodia safety team within 24 hours from the time that the event was identified. If further data are required, additional documentation can be submitted. All SAEs must be followed until resolution, or until the SAE is deemed permanent or leads to death.

Samples will be shipped for PCR to a molecular laboratory where they will be analysed in batches. Following quality control results will be available approximately 3-6 months from the time of collection. The list of positive tests will be returned to the field sites. If a participant is found to have a plasmodium infection, and has not already received antimalarial treatment subsequent to the sample being collected, then these individuals will be contacted by a local health worker, and if a participant reports fever or illness they will offered appropriate diagnosis and treatment.

Definitions

Adverse Event (AE)

An AE is any undesirable event or clinical deterioration that occurs to a study participant during the course of the study; that is, from the time of administration of study drugs until study ends (i.e., until the follow up visit) whether or not that event is considered related to the study drugs, or to a concomitant drug or procedure: e.g.

- any unfavourable and unintended symptom
- physical sign
- abnormal laboratory result
- an illness

Any new clinical sign or clinical deterioration that occurs between signing the consent form and the administration of study drugs is not an AE. This information will be recorded in the medical records, as a pre-existing condition.

Serious Adverse Event (SAE)

A serious adverse event is an AE that:

- results in death
- is life-threatening i.e. the participant was at risk of death at the time of the AE
- requires in participant hospitalisation or prolongation of existing hospitalisation
- results in persistent or significant disability/incapacity
- is a congenital anomaly/birth defect
- Any other significant medical condition

All of the above criteria apply to the case as a whole and should not be confused with the outcomes of individual reactions/events. More than one of the above criteria can be applicable to the one event. Important medical events that may not be immediately life-threatening or result in death or hospitalisation may be considered an SAE when, based upon appropriate medical judgment, they may jeopardise the participant or require medical or surgical intervention to prevent one of the

For peer review only

outcomes listed in the definition above. Examples of such medical events include allergic bronchospasm requiring intensive treatment in an emergency room or at home, blood dyscrasias or convulsions that do not result in hospitalisation, or development of drug dependency or drug abuse.

Reporting Procedures for Serious Adverse Events

All SAEs must be reported by the site investigator to the Study PI and PAL-Cambodia safety and medical monitor, within one day of his or her awareness of the SAE. The SAE report, should be emailed to the email paltrial@tropmedres.ac.

Further reports should be submitted, if required, until the SAE is resolved.

The site investigator must also report the SAEs to the local ethics committee in accordance with local requirements.

Evaluating Adverse Events and Serious Adverse Events

Assessment of Intensity

Each adverse event will be graded according to the Common Terminology Criteria for Adverse Events (CTCAE) Version 5.0 November 2017.

If an adverse event is not listed in the CTCAE table, the Investigator will assess the severity using the following guidelines:

1 = Mild; asymptomatic or mild symptoms; clinical or diagnostic observations only; intervention not indicated.

2 = Moderate; minimal, local or noninvasive intervention indicated; limiting age appropriate instrumental ADL*

3 = Severe or medically significant but not immediately life-threatening; hospitalization or prolongation of hospitalization indicated; disabling; limiting self care ADL**

4 = Life-Threatening consequences; urgent intervention indicated

5 = Death related to AE

Activities of Daily Living (ADL)

*Instrumental ADL refer to preparing meals, shopping for groceries or clothes, using the telephone, managing money, etc.

**Self care ADL refer to bathing, dressing and undressing, feeding self, using the toilet, taking

medications, and not bedridden.

Clarification of the difference in meaning between ‘severe’ and ‘serious’

The term “severe” is often used to describe the intensity (severity) of a specific event (as in mild, moderate, or severe myocardial infarction); the event itself, however, may be of relatively minor medical significance (such as severe headache). This is not the same as “serious”, which is based on the outcome or criteria defined under the serious adverse event definition. An event can be considered serious without being severe if it conforms to the seriousness criteria, similarly severe events that do not conform to the criteria are not necessarily serious. Seriousness (not severity) serves as a guide for defining regulatory reporting obligations.

Assessment of relatedness

The investigator is obligated to assess the relationship between study drug and the occurrence of each AE/SAE using the following categories of relatedness:

1. Definite: clear-cut temporal association

For peer review only

2. Probable: clear-cut temporal association, with improvement upon drug withdrawal, and not reasonably explained by the participant's known clinical state or other aetiology.
3. Possible: less clear temporal association; other aetiologies are possible. (Other possible aetiologies should be recorded on the CRF).
4. Not related: no temporal association with the study drug; assessed as related to other aetiologies such as concomitant medications or conditions, or participant's known clinical state.

The investigator will provide the assessment of causality as per the AE/SAE data collection tool.

Outcome

The investigator will follow-up the AE and SAE until resolution or until no further medically relevant information can be expected. AE and SAE outcome will be classified as follows:

1. Continuing/ongoing
2. Resolved
3. Resolved with sequelae
4. Permanent
5. Fatal

Statistical Considerations

Sample size justification

The target population for this study will be adult Cambodians who work and sleep in the forest (farmers, collect forest goods, hunting, etc.). 2,200 study participant episodes are required in each arm to have sufficient power to detect a statistically significant difference between the treatment arm and a control arm. An episode is defined as a follow-up period of 28 days with each enrolled individual contributing 1, 2 or 3 episodes. The estimate of the required sample size is complicated by the scarce data on *P. falciparum* incidence in forest workers.

Formally, we anticipate that the risk of being Pf positive without receiving prophylaxis will be around 5%. A total of 1,605 participant-episodes per arm are enough to detect a difference of at least 40% in the proportion of episodes with a Pf positive result as defined by the 28-day PCR parasite positivity rate i.e. from 5% positivity in participants without receiving antimalarial prophylaxis (i.e. multivitamin) to 3% positivity in participants receiving artemether-lumefantrine prophylaxis. This has been estimated with 80% power and 5% significance level. However, we also anticipate that we will likely observe multiple episodes being recruited into the study that can reduce power of the study if not accounted for. To compensate for the multiple episodes and any losses to follow up, we plan to recruit approximately 600 (i.e. 595) additional episodes in each group on top of the required 1605 single episodes. This gives an additional 27% episodes to account for the multiple episodes and losses to follow up. Thus, the overall sample size will be 4,400 episodes (i.e. 2,200 episodes in the treatment arm and 2,200 episodes in the control arm). The sample size calculations have been performed in Stata version 15.

Statistical Analyses

The main analysis strategy for the primary outcome will be the intention-to-treat (ITT) principle followed by the per protocol (PP) analysis. Thus, we will first analyse the ITT population in which all participants recruited in the trial will be included in the analysis according to the randomisation arm irrespective of what they actually got. These ITT analyses will be followed by the analysis of the per protocol (PP) population in which participants who did not adhere to the protocol will be excluded from analysis.

For peer review only

The composite primary endpoint will be analysed as follows. For 28-day PCR Plasmodium positivity and parasite positive clinical episode rate analysis, each arm will be summarised using crude proportions and binomial exact 95% confidence intervals. The risk differences in Plasmodium positivity between AL versus Multivitamin will be reported along with the corresponding 95% confidence intervals. Robust standard errors will be used to handle multiple episodes. These analyses will be complemented by the use of the crude Kaplan-Meier estimates of cumulative PCR Plasmodium positivity and parasite positive clinical episode probabilities as recommended by WHO. The incidence of confirmed clinical malaria between day 0 to day 28 analysis will be modeled using the mixed effects Poisson regression model to obtain incidence rate ratios (IRR) comparing AL versus Multivitamin arms. The mixed effects models will take into account the correlation of multiple episodes from the same participant. Tests of significance will be performed at 5% significance level. Analysis of all endpoints will be described in detail in a Statistical Analysis Plan finalised prior to locking the database. A brief overview is given below.

Proportions

These will be compared using chi squared or Fisher's exact test, as appropriate. Crude proportions will be calculated with the exact 95% confidence intervals (CI), where relevant.

Continuous data

These will be summarised by medians (IQR, ranges) and means (standard deviations, 95% CIs), as appropriate, and will include the parasite counts and laboratory parameters. Comparisons of continuous data will be assessed using the paired/unpaired t tests or the sign rank/Mann Whitney U tests, as appropriate.

Safety analysis

Safety analyses will be based on the whole population that get administered the study drug. Safety and tolerability of ACT versus multivitamin will be assessed by comparing the frequency (%) of adverse events and serious adverse events, with particular attention to abdominal pain, appetite perturbation, using the Fisher's exact test. Safety data will be presented in tabular and/or graphical format and summarised descriptively. Any clinically relevant abnormalities or values of potential clinically concern will be described. Participants will be analysed according to an intention to treat and a per protocol method where appropriate.

Handling of missing data

For analyses of proportions, missing outcomes will be imputed using plausible values. For example, worst-case scenario may be deemed appropriate and in that case sensitivity analysis will be performed with the best-case scenario. In the ITT Kaplan-Meier/survival analysis, participants who are lost to follow up, or who have Plasmodium reinfections or inconclusive PCR correction, will be censored from the moment of occurrence of one of these events. This survival analysis approach is the best way of handling missing data because participants with partial information are included in the analysis up to the time when they are lost/withdraw from the study.

Adverse events

Adverse events will be graded according to Common Terminology Criteria for Adverse Events (CTCAE) Version 5.0 November 2017.

All adverse events that are newly started or increased in intensity after the study drug administration will be reported. AE reports will be generated for all AEs that occurred after study drug administration, until the end of the study.

Mathematical modelling

The impact of the ACT artemether-lumefantrine as prophylaxis for forest goers on overall

For peer review only - http://bmjopen.bmj.com/site/about/guidelines.xhtml

malaria transmission will be quantified using mathematical modelling. For this we will develop a population dynamic village level individual-based model of malaria transmission and treatment parameterised with published data, results from the analysis of data from the trial and fitted to surveillance data from the study area.

Direct Access to Source Documents/Data

Direct access will be granted to authorised representatives from the sponsor and host institution, the regulatory authorities, and ethical committee (if applicable), to permit trial-related monitoring and inspections.

Quality Control and Quality Assurance Procedures

The study will be conducted in accordance with the current approved protocol, ICH GCP, any national regulations that may apply to this study and standard operating procedures. The WWARN will be engaged in assuring QA/QC of study execution in collaboration with the MORU Clinical Trials Support Group (CTSG). Their role will include but not be limited to monitoring adherence to SOPs for collection of laboratory specimens and quality checks (curation) of laboratory data according to standard methodologies.

Monitoring

Study sites may have in place a system for internal monitoring. In addition, regular external monitoring of all sites will be performed by the MORU CTSG according to ICH GCP and a Monitoring Plan. Data will be evaluated for compliance with the protocol and accuracy in relation to source documents. The monitors will check whether the clinical trial is conducted and data are generated, documented and reported in compliance with the protocol, GCP and the applicable regulatory requirements. Evaluation of on-site monitoring schemes, such as a reciprocal monitoring scheme, may be undertaken at selected sites by CTSG.

Patient and Public Involvement

During 2018, extensive consultations were held with local authorities, patients, and study communities regarding the design and organization of the trial. This included the district health authorities and Governor's office. The Siem Pang field station conducted malaria treatment studies and as part of these studies interviews and questionnaires were done with patients to better understand the local risk factors for malaria, travel histories, and the nature of forest work.[23] Specific questions on the time spent in forests was asked[24] and the use of medicines in forests, and the willingness to take antimalarial prophylaxis [Tripura et al in preparation]. A review of forest acquired malaria was prepared at the site in collaboration with local partners.[25] The potential importance of antimalarial prophylaxis was identified through understanding of the high risk of malaria infection in local forests and the willingness of participants to take medicine to prevent this. Through conversations with malaria patients treated at the health centre and from monthly meetings with village malaria health workers the design of the study was informed. This supported decisions about: time spent in forests, follow up scheduling, type of sample collection, monitoring of treatment compliance, suitable locations an communities where patients could be enrolled, concerns and questions surrounding adverse events, defining the messaging and rationale in local languages. Recruitment takes place in villages and community leaders and local health workers including village malaria workers are part of the study team. Patients previously enrolled in studies often serve as guides and assistants as they trust the study team and know the local community and as they are often forest workers themselves they know other forest workers like them who may be willing to participate. Conversations were held with patients and local stakeholders regarding feasibility and to ensure that participation in research would be acceptable and not burdensome or interfering with regular activities. From these discussions we adopted an outreach strategy so that follow up can take place without participants needing to travel long distances or to

For peer review only - http://bmjopen.bmj.com/site/about/guidelines.xhtml

the health centres. Dissemination will take place on several levels: in villages, at district level, at provincial level, and at a national and international level. As part of an ongoing research platform in the district we will communicate results back to study communities at the end of the trial by public meetings. A series of public engagement activities, including dissemination activities, has run alongside the CNM-MORU malaria field studies since 2013.[26]

For peer review only

ETHICS AND DISSEMINATION

Declaration of Helsinki

The Investigator will ensure that this study is conducted in compliance with the current revision of the Declaration of Helsinki (Fortaleza 2013).

ICH Guidelines for Good Clinical Practice

The Investigators will ensure that this study is conducted according to any National Regulations and that it will follow the principles of the ICH Guidelines for Good Clinical Practice.

Approvals

The study protocol and its associated documents will be submitted to the Oxford Tropical Research Ethics Committee (OxTREC) and the appropriate local ethics committees for written approval.

The Investigator will submit and, where necessary, obtain approval from the above parties for all substantial amendments to the original approved documents.

Risks

This study will use drugs that have been studied thoroughly and their toxicities are well described. In general, they are all well tolerated. In the event of any serious or severe adverse event participants will be referred to the local Referral Hospital where best available care will be provided.

Risks of artemether-lumefantrine

The safety of artemether and lumfantrine for treatment of malaria has been evaluated in clinical trials and, post licensing, widespread use for treating malaria in hundreds of millions of patients per year. Reported AL side effects have generally been mild. Reported adverse reactions in clinical trials have been similar or lower in frequency and magnitude to other ACTs. The commonest (>=3%) reported adverse events in clinical studies with AL in adults were headache, anorexia, dizziness and asthenia. AL is not known to cause harmful prolongation of the QTc interval.[27].

Risks of multivitamin

The main side-effects of multivitamin are upset stomach, unpleasant taste or headache which are mild to moderate in nature. Very rarely, these may cause an allergic reaction.

Risk of phlebotomy and finger prick

The primary risks of phlebotomy include local discomfort, occasional bleeding or bruising of the skin at the site of needle puncture, and rarely haematoma or infection. Phlebotomy will be performed by suitably qualified and trained staff using appropriate hygiene measures including gloves and alcohol swabs to clean the skin.

Risk of GPS data

Due to the potential unique nature of the GPS tracking data, it may be possible to identify individuals from their tracks. This will be minimized by the GPS tracking data being kept separately from any personally identifiable information and linked to the data collected on the study CRF only through a unique study code. The GPS tracking data will also be stored anonymously on the tracking device during collection and moved to an encrypted hard drive upon completion of collection.

5.1 Benefits

There are no anticipated direct benefits to the participants in this study. However, knowledge gained from this study is expected to help to assess the efficacy and feasibility of prophylaxis to prevent malaria in forest workers, and to predict its efficacy in reducing overall transmission. The proposed study is a critical step for future use of chemoprophylaxis to protect forest workers in the GMS against malaria.

For peer review only

Alternatives to Study Participation

Participants are able to decline freely participation in this study. If so, they will receive standard care for their malaria (if applicable).

Incentives & Compensation

Study participants will be compensated for time lost from work as a result of trial activities, the cost of local transport to attend for the follow up visits and will receive a per diem to cover the costs of meals on those days. The amounts in monetary terms will be determined by CNM in accordance with local norms.

The study will pay for treatment for drug-related SAEs or other research-related injuries. The study cannot pay for long term care for disability resulting from complications of the illness.

aality

The trial staff will ensure that the participants' anonymity is maintained. The participants will be identified only by initials and a study number on the CRF and electronic databases. All documents will be stored securely and be accessible to trial staff and authorised personnel only.

Sample Sharing and Storage

Samples collected will be used for the purpose of this study as stated in the protocol and stored for future use no longer than 10 years. Consent will be obtained from participants for sample storage and/or shipment of specific samples to collaborating institutions for investigations that cannot be performed locally. Any proposed plans to use samples other than for those investigations detailed in this protocol will be submitted to the relevant ethics committees prior to any testing. Material transfer agreements will be arranged and signed where appropriate/needed.

Data Handling and Record Keeping

Study data will be recorded on Case Report Forms (CRF) at the study sites and stored in a secure database. Validation checks will be built into the study database to identify missing values, inconsistencies, or invalid data. Additionally, study data will be profiled using statistical software to check for outliers and errors not detected by the database. All tasks related to data management will be carried out in accordance with the study data management plan.

Data sharing

De-identified, individual participant data from this study will be available to researchers whose proposed purpose of use is approved by the data access committee at Mahidol Oxford Tropical Medicine Research Unit. Inquiries or requests for the data may be sent to datasharing@tropmedres.ac.

Sponsorship and Insurance

The University of Oxford has a specialist insurance policy in place: - Newline Underwriting Management Ltd, at Lloyd's of London – which would operate in the event of any Participant suffering harm as a result of their involvement in the research.

Dissemination Plan

Results will be published in the open access peer-reviewed medical literature. Any data published will protect the identity of the participants. This trial will be registered in a web based protocol registration scheme. All those who have made a substantial contribution will be co-authors on publications. The sites have the right to publish their data individually and to include members of the sponsor's team who have made a significant contribution. There will also publications of pooled data

For peer review only

which will be coordinated by the MORU group. All sites will have the opportunity to contribute to these publications.

All the research findings from the programme and from relevant research outside the Programme will be analysed and integrated, and through the WHO Global Malaria Programme will be disseminated to policy makers, National Malaria Control Programmes (NMCPs) and other researchers.

SPIRIT Checklist

A completed Standard Protocol Items: Recommendations for Interventional Trials (SPIRIT) checklist for the protocol is provided in supplementary file 4.

For peer review only

REFERENCES

1. Guerra, C.A., R.W. Snow, and S.I. Hay, *A global assessment of closed forests, deforestation and malaria risk.* Ann Trop Med Parasitol, 2006. **100**(3): p. 189-204.
2. Thanh, P.V., et al., *Epidemiology of forest malaria in Central Vietnam: the hidden parasite reservoir.* Malar J, 2015. **14**: p. 86.
3. Erhart, A., et al., *Epidemiology of forest malaria in central Vietnam: a large scale cross-sectional survey.* Malar J, 2005. **4**: p. 58.
4. Sanh, N.H., et al., *Forest malaria in central Vietnam.* Am J Trop Med Hyg, 2008. **79**(5): p. 652-4.
5. Thang, N.D., et al., *Malaria in central Vietnam: analysis of risk factors by multivariate analysis and classification tree models.* Malar J, 2008. **7**: p. 28.
6. Erhart, A., et al., *Forest malaria in Vietnam: a challenge for control.* Am J Trop Med Hyg, 2004. **70**(2): p. 110-8.
7. Dysoley, L., et al., *Changing patterns of forest malaria among the mobile adult male population in Chumkiri District, Cambodia.* Acta Trop, 2008. **106**(3): p. 207-12.
8. Moore, S.J., et al., *Border malaria in China: knowledge and use of personal protection by minority populations and implications for malaria control: a questionnaire-based survey.* BMC Public Health, 2008. **8**: p. 344.
9. Gryseels, C., et al., *High Mobility and Low Use of Malaria Preventive Measures Among the Jarai Male Youth Along the Cambodia-Vietnam Border.* Am J Trop Med Hyg, 2015. **93**(4): p. 810-8.
10. Grietens, K.P., et al., *Social determinants of long lasting insecticidal hammock use among the Ra-glai ethnic minority in Vietnam: implications for forest malaria control.* PLoS One, 2012. **7**(1): p. e29991.
11. Lengeler, C., *Insecticide-treated bednets and curtains for preventing malaria.* Cochrane Database Syst Rev, 2000(2): p. CD000363.
12. Thang, N.D., et al., *Long-Lasting Insecticidal Hammocks for controlling forest malaria: a community-based trial in a rural area of central Vietnam.* PLoS One, 2009. **4**(10): p. e7369.
13. Son, D.H., et al., *The prevalence, incidence and prevention of Plasmodium falciparum infections in forest rangers in Bu Gia Map National Park, Binh Phuoc province, Vietnam: a pilot study.* Malar J, 2017. **16**(1): p. 444.
14. York, A., *Seasonal malaria chemoprevention in the Sahel.* Lancet Infect Dis, 2017. **17**(6): p. 588.
15. Snow, R.W., *Seasonal Malaria Chemoprevention: An Evolving Research Paradigm.* PLoS Med, 2016. **13**(11): p. e1002176.
16. Tripura, R., et al., *A Controlled Trial of Mass Drug Administration to Interrupt Transmission of Multidrug-Resistant Falciparum Malaria in Cambodian Villages.* Clin Infect Dis, 2018. **67**(6): p. 817-826.
17. Pryce, J. and P. Hine, *Pyronaridine-artesunate for treating uncomplicated Plasmodium falciparum malaria.* Cochrane Database Syst Rev, 2019. **1**: p. CD006404.
18. Chaumeau, V., et al., *Contribution of Asymptomatic Plasmodium Infections to the Transmission of Malaria in Kayin State, Myanmar.* J Infect Dis, 2019. **219**(9): p. 1499-1509.
19. Imwong, M., et al., *High-throughput ultrasensitive molecular techniques for quantifying low-density malaria parasitemias.* J Clin Microbiol, 2014. **52**(9): p. 3303-9.
20. Imwong, M., et al., *The epidemiology of subclinical malaria infections in South-East Asia: findings from cross-sectional surveys in Thailand-Myanmar border areas, Cambodia, and Vietnam.* Malar J, 2015. **14**: p. 381.
21. Saunders, D.L., et al., *Dihydroartemisinin-piperaquine failure in Cambodia.* N Engl J Med, 2014. **371**(5): p. 484-5.
22. Organization, W.H., *WHO Model List of Essential Medicines*. 2017.
23. Sanann, N., et al., *Forest work and its implications for malaria elimination: a qualitative study.* Malar J, 2019. **18**(1): p. 376.
24. von Seidlein, L., et al., *Novel Approaches to Control Malaria in Forested Areas of Southeast Asia.* Trends Parasitol, 2019. **35**(6): p. 388-398.

For peer review only - http://bmjopen.bmj.com/site/about/guidelines.xhtml

25. Nofal, S.D., et al., *How can interventions that target forest-goers be tailored to accelerate malaria elimination in the Greater Mekong Subregion? A systematic review of the qualitative literature.* Malar J, 2019. **18**(1): p. 32.
26. Lim, R., et al., *Drama as a community engagement strategy for malaria in rural Cambodia.* Wellcome Open Res, 2017. **2**: p. 95.
27. Haeusler, I.L., et al., *The arrhythmogenic cardiotoxicity of the quinoline and structurally related antimalarial drugs: a systematic review.* BMC Med, 2018. **16**(1): p. 200.

For peer review only

AUTHORS' CONTRIBUTIONS

- Richard J Maude, Co-Principal Investigator, designed the study, wrote the protocol.
- Rupam Tripura, contributed to overall study design, edited the protocol.
- Mom Ean, contributed to design of the field data collection, edited the protocol.
- Meas Sohka, contributed to design of the field data collection, edited the protocol.
- Thomas J Peto, contributed to overall study design, edited the protocol.
- James J Callery, contributed to overall study design, edited the protocol.
- Mallika Imwong, contributed to study design for PCR and genetic analysis, edited the protocol.
- Ranitha Vongpromek, contributed to study design for sample collection, edited the protocol.
- Joel Tarning, contributed to study design for the dosing schedule and analysis for lumefantrine levels, edited the protocol.
- Mavuto Mukaka, contributed to study design for randomization, sample size calculation and statistical analysis, edited the protocol.
- Naomia Waithira, designed the data management plan, edited the protocol.
- Oung Soviet, contributed to study design for field data collection, edited the protocol.
- Lorenz von Seidlein, contributed to overall study design, edited the protocol.
- Siv Sovannaroth, Co-Principal Investigator, designed the study, edited the protocol.

For peer review only

FUNDING STATEMENT

This work was supported by The Global Fund to Fight AIDS, Tuberculosis and Malaria grant number B9R03070 and the Wellcome Trust of Great Britain grant number 106698/Z/14/Z.

COMPETING INTERESTS STATEMENT

The authors declare no competing interests.

SUPPLEMENTARY FILES

1. Schedule of assessments and dosing schedules
2. Patient Information Sheet
3. Informed Consent Form
4. SPIRIT checklist

DATE AND VERSION

18th May 2021

Version 2

ROLE OF THE SPONSOR AND FUNDERS

The sponsor and funder had no role in the in study design; collection, management, analysis, and interpretation of data; writing of the report; or the decision to submit the report for publication and they do not have ultimate authority over any of these activities..

For peer review only

Schedule of Assessments

TEST/APPLICATION	SCREENING	D0	D1	D2	CCM*	D28-35	CCM*	D56-63	CCM*	D84-91	CCM* up to D112
Informed consent	X										
Demographics		X									
Risk history		X				X		X		X	
Travel history		X				X		X		X	
Medical and drug history		X									
Symptoms questionnaire		X				X		X		X	
Temperature		X									
Weight		X									
Randomisation and assign study ID		X									
AL/multivitamin doses to be given		X	X	X							
Plasmodium PCR		X				X		X		X	
Blood for storage (serology)		X									
Plasmodium genetic analysis (DBS)					X*		X*		X*		X*
*CCM = episode of confirmed clinical malaria between enrolment and follow-up											

For peer review only

Dosing Schedules

Artemether-lumefantrine

Artemether-lumefantrine dosing schedule										
One tablet AL contains 20 mg artemether and 120 mg lumefantrine (Coartem)										
	No. of tablets recommended at approximate timing of dosing									
	Day 1		Day 2		Day 3		Day 8		Weekly	
Weight: Kilogram	0h	8h	24h	36h	48h	60h	168h	176h	336h …	344h …
15 - <25	2	2	2	2	2	2	2	2	2	2
25 - <35	3	3	3	3	3	3	3	3	3	3
≥35	4	4	4	4	4	4	4	4	4	4
Alternative preparation: one tablet AL contains 80 mg artemether and 480 mg lumefantrine (Artefan)										
	No. of tablets recommended at approximate timing of dosing									
	Day 1		Day 2		Day 3		Day 8		Weekly	
Weight: Kilogram	0h	8h	24h	36h	48h	60h	168h	176h	336h …	344h …
≥35	1	1	1	1	1	1	1	1	1	1

Multivitamin

Multivitamin dosing schedule					
One tablet contains Vitamin-A : 5000 USP units<br>Vitamin D: 400 USP Units<br>Ascorbic acid: 75 mg<br>Thiamine Mononitrate: 2 mg<br>Riboflavin: 3 mg<br>Niacin amide: 20 mg<br>Or suitable equivalent alternative					
	No. of tablets recommended at approximate timing of dosing				
	Day 1	Day 2	Day 3	Day 8	Weekly
Weight: Kilogram	0h	24h	48h	168h	336h …
≥15	1	1	1	1	1

view only

Participant Information Sheet

In this information sheet, we will give you information about the study to help you decide whether or not you agree to take part in this study. If you have any questions or concerns, please ask the study staff to help explain until you fully understand. You may ask to take this document home to discuss with relatives, siblings, close friends, or your doctor to assist you in making a decision to take part in this study.

**Lay Title**	Study to assess efficacy of antimalarial drugs to prevent forest goers from getting malaria in Cambodia
**Study Title (official)**	An open-label individually randomised controlled trial to assess the efficacy of artemether-lumefantrine prophylaxis for malaria among forest goers in Cambodia
**Principal Investigator**	Professor R.J. Maude
**Sponsor**	University of Oxford

If you are reading this to consider enrolling your child, please know that whenever it refers to 'you' it can be taken to mean 'your child'.

What is the purpose of this research?

In Cambodia, the number of malaria cases for forest goers is still high, due to the mobile nature of their work and their habit of sleeping overnight in the forests, often without mosquito nets. Therefore, we would like to do research on how to prevent malaria for forest goers. This study plans to recruit up to 4,400 participants from 50 villages in Stung Treng and Pursat Provinces who are planning to travel to the forest within the next 72 hours.

What will happen to you if you participate in the study?

We would like to invite you to join the study because you are 16 and 65 years of age and planning to visit the forest within 72 hours and stay overnight. The study staff will explain to you about details of the study and answer any questions you may have. If you are interested in joining the study, you will be asked to sign an informed consent form and then follow study procedures. You will go through study visits as below;

Screening and baseline assessment visit

During this visit, study staff will ask you questions about your health status and medication that you used and are currently using, risk factors for malaria, recent travel history and where you live and will go through a brief physical examination. If you pass all of the study selection criteria, you can take part in this study.

All the study participants will received either artemether-lumefantrine or multivitamin given by mouth over 3 days. The decision on which drug will be decided by random chance like a flip of a coin. This means that you will have the same chance of receiving artemether-lumefantrine or multivitamin. The artemether-lumefantrine should be taken with a small amount of fatty food or drink, e.g. a small carton of milk, so it is better absorbed into the body.

For peer review only - http://bmjopen.bmj.com/site/about/guidelines.xhtml

Participant Information Sheet

Before giving you the study drugs (artemether-lumefantrine or multivitamin), the study team will collect about 3.2 milliliters (around two thirds of a teaspoon) of your blood for malaria tests, and to measure the amount of lumefantrine present and some of this will be stored for testing later for other causes of infection.

The study team will observe when you take study drug on the first day. Where possible, study team will observe when you take study drug on days 2 and 3. If not possible, the study team will contact you by telephone or in person to ensure that you take the second and third doses of study drug and to ensure you follow below procedure in case of vomiting.

If you vomit within half an hour after taking the study drug, the full dose will be repeated. If vomiting occurs between half and one hour, half of the dose will be repeated. If vomiting occurs more than one hour after taking study drug, no repeat dosing will be done. If vomiting within 1 hour occurs more than one time, no repeat dosing is allowed.

In some participants, we would like to collect GPS coordinates of where you live and work and places where the malaria infection may occur, such as forests, farms or plantations. This may include being asked to carry a device to record your location over time which can choose not to carry if you are not comfortable doing so. We will collect all available local malaria treatment records to describe how the study participants compare to the overall population who receive treatment for malaria and this will allow us to better understand local malaria epidemiology and transmission patterns. All personal information will be anonymised so that no individual can be identified from their treatment records, through interviews, or from mapping data.

Follow-up visit every 28-35 days after starting prophylaxis

You will be asked to return for a follow-up visit any time from 28 to 35 days after starting prophylaxis. During this visit, we will ask you about time you spent in the forest, where you went, why and who you travelled with and about any risk factors of getting malaria infection, and any illnesses or malaria infection occurred from the last visit. In addition, we will collect around 1.6 milliliters (around a third of a teaspoon) of your blood for malaria tests and to measure the amount of lumefantrine present. We also will collect around 2 milliliters (around half a teaspoon) of your blood to test later for other causes of infection.

If you are planning to return to the forest again within the following 28 days after this visit, you will be asked to continue your weekly prophylaxis of study drugs the same as what you received before. You will then be asked to return for a second follow-up visit 28 to 35 days later when the above procedure will be repeated. This will be repeated for a possible third visit giving a maximum total of three periods of 28 to 35 days. You will receive the same study drug throughout.

If you then plan to visit the forest again following an interval of at least 28 days after the last follow-up visit, you may be enrolled again into the study when you would receive either artemether-lumefantrine or multivitamin as described above. The choice of artemether-lumefantrine or multivitamin may be different. This may be repeated up to 1 more time thus giving a maximum of 3 courses of study drug.

At each follow-up visit, if you are not planning to return to the forest in the next 28 days, you will be asked to take 4 weeks of prophylaxis from the time you were last in the forest.

For peer review only

Participant Information Sheet

If you are not well at any time during the study participation, such as high fever, you will be assessed and treated by the healthcare workers in the local healthcare system as per routine clinical practice. We will collect your blood for malaria tests.

Are there any risks or disadvantages to me for taking part?

Risk of blood withdrawal from the arm

The risks of blood withdrawal from the arm include discomfort, occasional bleeding or bruising of the skin at the site of needle puncture, and rarely infection. If an infection of the skin occurs, you will be taken care according to standard of care until you recover without any expenses.

Risk of Artemether-lumefantrine

Artemether-lumefantrine is registered for the treatment of malaria. It is usually very well tolerated. The most common side-effects are nausea, vomiting, abdominal pain and diarrhoea. Some people experience headache and dizziness.

Risks of multivitamin

The main side-effects of multivitamin are upset stomach, unpleasant taste or headache which are mild to moderate in nature. Very rarely, there may cause an allergic reaction.

There may be any side effects from study drugs that have not been noticed before. You should inform the study staff right away if you have any problems. If you have any side effects or any unexpected problems during participation in the study, we will treat these problems fully and with no charge to you.

The study drugs may not be safe for an unborn child. If you are female with child bearing potential, it is essential that you do not become pregnant during the study participation. This means not having sex or using a proven and effective method of birth control such as birth control pills, intrauterine contraceptive devices, vaginal contraceptive devices or hormonal implants. If you become pregnant, you will be followed to determine the outcome of the pregnancy. Please tell us as quickly as possible when you become pregnant during the study.

If any new information about the safety of the study drugs becomes available, we will tell you as soon as possible.

Protection against malaria

It is not known how effective artemether-lumefantrine is to prevent malaria and that the multivitamin will provide no protection against malaria. You should therefore assume that you are not protected from catching malaria by taking these medicines

What are the benefit of taking part in this study?

The results of the study will help us to understanding if the Artemether-lumefantrine could protect forest workers from malaria infection. This also may help to predict its ability in reducing malaria transmission.

What will happen if you choose not to take part in the study, or if you change your mind after you agree?

Your participation in this study is entirely voluntary. After you join the study, you are also free to withdraw from the study at any time, without penalty or any effects on your medical treatment both now and in the future.

For peer review only - http://bmjopen.bmj.com/site/about/guidelines.xhtml

Participant Information Sheet

In addition, the study staff and the study sponsor have rights to withdraw you from this study if it's considered that it is in your best interest.

What will the blood sample be used for?

If you have confirmed malaria infection, your blood sample will be processed to analyse the DNA of the parasite. To do this test, we have to ship your blood sample to Molecular Tropical Medicine Laboratory, Bangkok, Thailand and/or Wellcome Trust Sanger Institute in Hinxton, UK or other suitable laboratory.

Total volume of blood collection will be approximately 9.6 milliliters (around two teaspoons) for the maximum of 84 days of follow up if you return for follow-up on 3 consecutive occasions and had all blood samples taken. This blood volume does not include any repeat blood test for which an additional 9.6 milliliters (around two teaspoons) may be needed if necessary.

If you consent, we would like to keep some of your blood samples for not longer than 10 years. Your blood samples will be labeled with a unique number but not with your name. Any additional testing apart from what indicated in this study will be tested only after permission by the ethics committee.

Will there be any financial cost or compensation for participating the study?

It should not cost you any money to join the study. You will be compensated for time lost from work as a result of study activities plus the cost of local transport to attend each study visit and the costs of meals on those days. *(The amounts in monetary terms will be determined by CNM according to local norms).*

Confidentiality

Your name will not be in any report or on any blood sample being collected during the study. The information we collected from you and from analysing of your blood samples will be kept confidential by the study team.

No one other than the study team, authorised personnel from the study sponsor, monitor, ethics committee and regulatory authorities are allowed direct access to personally identified study records.

If you allow all data collected from you including results from blood analyses that is stored in our database may be shared with other researchers to use in the future. The other researchers will not be given any information that could identify you.

Who can I contact if I have questions?

If you have any questions or concerns after reading this information sheet, you can discuss them with our study team. We will also available throughout the study to answer any questions or address any concerns that you may have later on.

If you have any questions, you can contact the following doctors/study staffs by telephone:

Name: *Telephone number:*

or

Name: *Telephone number:*

For peer review only - http://bmjopen.bmj.com/site/about/guidelines.xhtml

Participant Information Sheet

If you haven't been treated as specified in this information sheet or you wish to know the participant's rights, contact the secretariat office of the Ethics Committee. *<Ethics committee contact information>.*

Data protection

The University of Oxford is responsible for ensuring the safe and proper use of any personal information you provide, solely for research purposes.

For peer review only

For peer review only - http://bmjopen.bmj.com/site/about/guidelines.xhtml

Informed Consent Form

I would like to take part in the study, titled: **"**An open-label individually randomised controlled trial to assess the efficacy of artemether-lumefantrine prophylaxis for malaria among forest goers in Cambodia**".**

I have read the participant information sheet and have had the opportunity to ask questions about the study and any questions I have asked have been answered to my satisfaction.

I understand that I can withdraw myself/my child or stop taking part in the research at any time without affecting further services to which I am/my child is entitled in the future. To consent to take part in this study, I allow the study team to use my/my child's personal information obtained from this study.

If I have doubts about the study procedures or I/my child experience(s) any side effect from this study, I will be able to contact study staff at any time.

I fully understand the statements in the participant information sheet and this informed consent form, and consent to participate in this study.

And

I ☐ allow / ☐ do not allow my/my child's blood to be tested for other infectious diseases.

I ☐ allow / ☐ do not allow my/my child's blood to be stored for future studies.

I ☐ allow / ☐ do not allow my/my child's blood to be shipped abroad.

I ☐ allow / ☐ do not allow my/my child's data and results from blood analyses that is stored in the database to be shared with other researchers to use in the future.

I ☐ allow / ☐ do not allow my/my child's location to be recorded with a GPS device.

Signature of participant/parent or guardian (if child) ……………………………………

Print name of participant/parent or guardian (if child) ……………………………………

Date ……………………………………

Signature of person conducting the informed consent ………………………………

Print name of person conducting the informed consent ………………………………

Date ………………………………

THE PARTICIPANT SHOULD NOW BE GIVEN A SIGNED COPY TO KEEP

For peer review only - http://bmjopen.bmj.com/site/about/guidelines.xhtml

For peer review only

Informed Consent Form

For the participant who cannot read or sign in the consent form, the participant can thumb print in the following box.

I cannot read but study staffs have read information in this informed consent form to me and explain until I fully understand the given information. Therefore I provide my thumbprint to voluntary consent for myself taking part in this research study.

And

I ☐ allow / ☐ do not allow my/my child's blood to be tested for other infectious diseases.

I ☐ allow / ☐ do not allow my/my child's blood to be stored for future studies.

I ☐ allow / ☐ do not allow my/my child's blood to be shipped abroad.

I ☐ allow / ☐ do not allow my/my child's data and results from blood analyses that is stored in the database to be shared with other researchers to use in the future.

I ☐ allow / ☐ do not allow my/my child's location to be recorded with a GPS device.

☐

Right thumb print of the participant/parent or guardian (if child)

Signature of person conducting the informed consent ……………………………

Print name of person conducting the informed consent ……………………………

Date ……………………………

Signature of witness …………………………………………………

Print name of witness …………………………………………………

Date …………………………………………………..

THE PARTICIPANT SHOULD NOW BE GIVEN A SIGNED COPY TO KEEP

For peer review only

For peer review only - http://bmjopen.bmj.com/site/about/guidelines.xhtml

STANDARD PROTOCOL ITEMS: RECOMMENDATIONS FOR INTERVENTIONAL TRIALS

SPIRIT 2013 Checklist: Recommended items to address in a clinical trial protocol and related documents*

Section/item	Item No	Description	Addressed on page number
**Administrative information**			
Title	1	Descriptive title identifying the study design, population, interventions, and, if applicable, trial acronym	1____________
Trial registration	2a	Trial identifier and registry name. If not yet registered, name of intended registry	2____________
	2b	All items from the World Health Organization Trial Registration Data Set	Multiple_______
Protocol version	3	Date and version identifier	27___________
Funding	4	Sources and types of financial, material, and other support	27___________
Roles and responsibilities	5a	Names, affiliations, and roles of protocol contributors	1,26_________
	5b	Name and contact information for the trial sponsor	22___________
	5c	Role of study sponsor and funders, if any, in study design; collection, management, analysis, and interpretation of data; writing of the report; and the decision to submit the report for publication, including whether they will have ultimate authority over any of these activities	27___________
	5d	Composition, roles, and responsibilities of the coordinating centre, steering committee, endpoint adjudication committee, data management team, and other individuals or groups overseeing the trial, if applicable (see Item 21a for data monitoring committee)	N/A__________

For peer review only

Introduction

Background and rationale	6a	Description of research question and justification for undertaking the trial, including summary of relevant studies (published and unpublished) examining benefits and harms for each intervention	4-5__________
	6b	Explanation for choice of comparators	4____________
Objectives	7	Specific objectives or hypotheses	6____________
Trial design	8	Description of trial design including type of trial (eg, parallel group, crossover, factorial, single group), allocation ratio, and framework (eg, superiority, equivalence, noninferiority, exploratory)	6____________

Methods: Participants, interventions, and outcomes

Study setting	9	Description of study settings (eg, community clinic, academic hospital) and list of countries where data will be collected. Reference to where list of study sites can be obtained	6____________
Eligibility criteria	10	Inclusion and exclusion criteria for participants. If applicable, eligibility criteria for study centres and individuals who will perform the interventions (eg, surgeons, psychotherapists)	7-8__________
Interventions	11a	Interventions for each group with sufficient detail to allow replication, including how and when they will be administered	9-10, Appendix 2-3
	11b	Criteria for discontinuing or modifying allocated interventions for a given trial participant (eg, drug dose change in response to harms, participant request, or improving/worsening disease)	8,14-15_______
	11c	Strategies to improve adherence to intervention protocols, and any procedures for monitoring adherence (eg, drug tablet return, laboratory tests)	13___________
	11d	Relevant concomitant care and interventions that are permitted or prohibited during the trial	13___________
Outcomes	12	Primary, secondary, and other outcomes, including the specific measurement variable (eg, systolic blood pressure), analysis metric (eg, change from baseline, final value, time to event), method of aggregation (eg, median, proportion), and time point for each outcome. Explanation of the clinical relevance of chosen efficacy and harm outcomes is strongly recommended	7____________
Participant timeline	13	Time schedule of enrolment, interventions (including any run-ins and washouts), assessments, and visits for participants. A schematic diagram is highly recommended (see Figure)	Appendix 1____

For peer review only - http://bmjopen.bmj.com/site/about/guidelines.xhtml

Sample size	14	Estimated number of participants needed to achieve study objectives and how it was determined, including clinical and statistical assumptions supporting any sample size calculations	18___________
Recruitment	15	Strategies for achieving adequate participant enrolment to reach target sample size	8____________

Methods: Assignment of interventions (for controlled trials)

Allocation:

Sequence generation	16a	Method of generating the allocation sequence (eg, computer-generated random numbers), and list of any factors for stratification. To reduce predictability of a random sequence, details of any planned restriction (eg, blocking) should be provided in a separate document that is unavailable to those who enrol participants or assign interventions	9____________
Allocation concealment mechanism	16b	Mechanism of implementing the allocation sequence (eg, central telephone; sequentially numbered, opaque, sealed envelopes), describing any steps to conceal the sequence until interventions are assigned	9____________
Implementation	16c	Who will generate the allocation sequence, who will enrol participants, and who will assign participants to interventions	8-9__________
Blinding (masking)	17a	Who will be blinded after assignment to interventions (eg, trial participants, care providers, outcome assessors, data analysts), and how	9____________
	17b	If blinded, circumstances under which unblinding is permissible, and procedure for revealing a participant's allocated intervention during the trial	N/A__________

Methods: Data collection, management, and analysis

Data collection methods	18a	Plans for assessment and collection of outcome, baseline, and other trial data, including any related processes to promote data quality (eg, duplicate measurements, training of assessors) and a description of study instruments (eg, questionnaires, laboratory tests) along with their reliability and validity, if known. Reference to where data collection forms can be found, if not in the protocol	10-15_________
	18b	Plans to promote participant retention and complete follow-up, including list of any outcome data to be collected for participants who discontinue or deviate from intervention protocols	11,14________

For peer review only - http://bmjopen.bmj.com/site/about/guidelines.xhtml

Data management	19	Plans for data entry, coding, security, and storage, including any related processes to promote data quality (eg, double data entry; range checks for data values). Reference to where details of data management procedures can be found, if not in the protocol	22___________
Statistical methods	20a	Statistical methods for analysing primary and secondary outcomes. Reference to where other details of the statistical analysis plan can be found, if not in the protocol	18-19__________
	20b	Methods for any additional analyses (eg, subgroup and adjusted analyses)	18-19__________
	20c	Definition of analysis population relating to protocol non-adherence (eg, as randomised analysis), and any statistical methods to handle missing data (eg, multiple imputation)	19___________
**Methods: Monitoring**			
Data monitoring	21a	Composition of data monitoring committee (DMC); summary of its role and reporting structure; statement of whether it is independent from the sponsor and competing interests; and reference to where further details about its charter can be found, if not in the protocol. Alternatively, an explanation of why a DMC is not needed	20___________
	21b	Description of any interim analyses and stopping guidelines, including who will have access to these interim results and make the final decision to terminate the trial	N/A___________
Harms	22	Plans for collecting, assessing, reporting, and managing solicited and spontaneously reported adverse events and other unintended effects of trial interventions or trial conduct	16-18__________
Auditing	23	Frequency and procedures for auditing trial conduct, if any, and whether the process will be independent from investigators and the sponsor	20___________
**Ethics and dissemination**			
Research ethics approval	24	Plans for seeking research ethics committee/institutional review board (REC/IRB) approval	2____________
Protocol amendments	25	Plans for communicating important protocol modifications (eg, changes to eligibility criteria, outcomes, analyses) to relevant parties (eg, investigators, REC/IRBs, trial participants, trial registries, journals, regulators)	21___________

Consent or assent	26a	Who will obtain informed consent or assent from potential trial participants or authorised surrogates, and how (see Item 32)	8_____________
	26b	Additional consent provisions for collection and use of participant data and biological specimens in ancillary studies, if applicable	23___________
Confidentiality	27	How personal information about potential and enrolled participants will be collected, shared, and maintained in order to protect confidentiality before, during, and after the trial	23____________
Declaration of interests	28	Financial and other competing interests for principal investigators for the overall trial and each study site	28____________
Access to data	29	Statement of who will have access to the final trial dataset, and disclosure of contractual agreements that limit such access for investigators	20____________
Ancillary and post-trial care	30	Provisions, if any, for ancillary and post-trial care, and for compensation to those who suffer harm from trial participation	23____________
Dissemination policy	31a	Plans for investigators and sponsor to communicate trial results to participants, healthcare professionals, the public, and other relevant groups (eg, via publication, reporting in results databases, or other data sharing arrangements), including any publication restrictions	23-24___________
	31b	Authorship eligibility guidelines and any intended use of professional writers	N/A___________
	31c	Plans, if any, for granting public access to the full protocol, participant-level dataset, and statistical code	23____________
**Appendices**			
Informed consent materials	32	Model consent form and other related documentation given to participants and authorised surrogates	Supplementary_
Biological specimens	33	Plans for collection, laboratory evaluation, and storage of biological specimens for genetic or molecular analysis in the current trial and for future use in ancillary studies, if applicable	12,23__________

*It is strongly recommended that this checklist be read in conjunction with the SPIRIT 2013 Explanation & Elaboration for important clarification on the items. Amendments to the protocol should be tracked and dated. The SPIRIT checklist is copyrighted by the SPIRIT Group under the Creative Commons "Attribution-NonCommercial-NoDerivs 3.0 Unported" license.

For peer review only - http://bmjopen.bmj.com/site/about/guidelines.xhtml
